# Genotype and microbiome shape immunity in a sex-specific manner in mouse models of Alzheimer’s disease

**DOI:** 10.1016/j.bbi.2025.07.028

**Published:** 2025-07-28

**Authors:** John W. Bostick, T. Jaymie Connerly, Taren Thron, Brittany D. Needham, Matheus de Castro Fonseca, Rima Kaddurah-Daouk, Rob Knight, Sarkis K. Mazmanian

**Affiliations:** a Division of Biology and Biological Engineering, California Institute of Technology, 1200 E California Blvd, Pasadena, CA 91125, USA; b Department of Psychiatry and Behavioral Sciences, Duke University, 905 W Main St, Durham, NC 27701, USA; c Duke Institute of Brain Sciences, Duke University, 308 Research Dr, Durham, NC 27710, USA; d Department of Medicine, Duke University, 40 Duke Medicine Cir Rm 401, Davison Bldg, Durham, NC 27710, USA; e Department of Pediatrics, University of California San Diego, 9461 Gilman Dr, La Jolla, CA 92093, USA; f Shu Chien-Gene Lay Department of Bioengineering, University of California San Diego, 9500 Gilman Dr, MC 0412, La Jolla, CA 92093, USA; g Department of Computer Science & Engineering, University of California San Diego, 9500 Gilman Dr, MC 0404, La Jolla, CA 92093, USA; h Halicioğlu Data Science Institute, University of California San Diego, 3234 Matthews Ln, La Jolla, CA 92093, USA

**Keywords:** Alzheimer’s disease, Animal models, 3xTg, 5xFAD, Mice, Microbiome, Germ-free, Adaptive immunity, Inflammation, Sex-specific

## Abstract

Preclinical studies have revealed that the microbiome broadly affects immune responses and deposition and/or clearance of amyloid-beta (Aβ) in mouse models of Alzheimer’s disease (AD), but whether, and how, the microbiome shapes central and peripheral immune profiles in AD models remains unknown. We examined adaptive immune responses in two mouse models containing AD-related genetic predispositions (3xTg and 5xFAD) in the presence or absence of the microbiome to determine if it promotes dysregulated immune responses and cognition in AD. T and B cells were altered in central nervous system (CNS)-associated lymph nodes and systemic immune tissues between genetic models and wildtype mice, with earlier signs of heightened immune activity in females. Systemic immune responses were modulated by the microbiome and differed by sex. Further, the absence of a microbiome in germ-free mice resulted in increased cognitive deficits, primarily in males. These data reveal sexual dimorphism in early signs of immune activity and microbiome effects, and highlight how sex and the microbiome shape responses in mouse models of AD.

## 1. Introduction

Alzheimer’s disease (AD) is the most prevalent neurodegenerative disease, and its incidence is expected to increase further as the global population ages (2023 Alzheimer’s disease facts and figures). Considerable progress has been made in understanding cellular and molecular contributors to AD; however, much remains unknown about its development and pathological mechanisms. Approximately two-thirds of AD cases in the United States are in females, prompting the question of how sex influences susceptibility (2023 Alzheimer’s disease facts and figures). Furthermore, although many people accumulate amyloid-β (Aβ) plaques, a key pathological hallmark of AD, only a subset will develop AD. Genetics cannot completely explain these differences (2023 Alzheimer’s disease facts and figures; Andrews et al., 2023). Recent research has implicated lifestyle and environmental factors, including diet and the microbiome: the collection of microorganisms that colonize the human body (Minter et al., 2017; Minter et al., 2016; Harach et al., 2017; Seo and Holtzman, 2024; Wang et al., 2019; Walker et al., 2017). Animal models, such as transgenic rodents, provide an opportunity to explore these questions.

AD is characterized by the accumulation of Aβ plaques and tau-mediated neurofibrillary tangles, leading to neuroinflammation, impaired synaptic communication, neuronal death, and decline in cognitive function. Two widely used models of AD are the 3x transgenic (3xTg) and 5x familial Alzheimer’s disease (5xFAD) mouse models (Oddo et al., 2003; Oakley et al., 2006). These preclinical models employ overexpression of gene variants with known AD association to translate human pathophysiology into mice. Both models develop AD-associated Aβ aggregates, with a well-characterized time course. The 3xTg model, but not the 5xFAD model, also expresses the tau protein, with fibrils accumulating by 12 months (Javonillo et al., 2022). While the immune response in the central nervous system (CNS) of both animal models has been extensively characterized, less is known about immune responses in peripheral tissues outside the CNS or the effect of the microbiome on immunity.

Prior evidence from AD models has revealed inflammation associated with Aβ and tau accumulation in the CNS (Newcombe et al., 2018). Inflammatory responses by astrocytes and microglia, two cell types that interact closely with neurons and promote their function, may contribute to initial innate immune responses (Shi and Holtzman, 2018). These cells are important components of protective Aβ clearance pathways. The adaptive immune response is directed at specific molecular targets that activate T and B cell receptors in an antigen-specific manner. As pathology progresses, the adaptive immune system is activated, although the initiating events, molecular targets, and timing are not well understood. Nonetheless, T and B cell responses can be protective in AD animal models, as genetic depletion of these cells results in greater accumulation of plaques and increased cytokine production (Marsh et al., 2016). Paradoxically, eliminating B cells alone is protective against AD-like progression in mice, indicating a B cell contribution to pathogenic processes (Kim et al., 2021). Additionally, T cell responses have been shown to exacerbate tauopathy progression (Chen et al., 2023; Gate et al., 2020; Wang et al., 2024; Laurent et al., 2017). Thus, the protective and pathogenic contributions of T and B cells to AD require further study.

The intestinal microbiome influences outcomes related to AD (Wang et al., 2019; Zhang et al., 2023). In AD mouse models, germ-free (GF) conditions or antibiotic treatment reduce Aβ aggregates in the brain (Minter et al., 2017; Minter et al., 2016; Harach et al., 2017). In addition, the microbiome regulates inflammatory responses in AD (Seo and Holtzman, 2024; Wang et al., 2019). Microbes and microbe-produced factors may affect AD in several ways, but one important aspect is activation of immune cells (Bostick et al., 2022; Needham et al., 2020). Previous studies have identified that the microbiome may influence AD outcomes in a sex-specific manner and could involve hormone-microbiome-AD pathology interactions (Cuervo-Zanatta et al., 2021; Dunham et al., 2024; Carroll et al., 2010; Farkas et al., 2022; Quinn et al., 2022; Carroll et al., 2007; Kapadia et al., 2018; Dennison et al., 2021; Manji et al., 2019). However, understanding the mechanisms by which the microbiome exerts these influences on AD requires further investigation.

Herein, we investigated immune responses in mice with AD-associated genetic landscapes in the presence or absence of a microbiome by comparing GF mice to conventionally raised specific pathogen-free (SPF) mice, i.e., those with standard laboratory microbiota, with the same genotype to determine if the microbiome promotes dysregulated immune responses and cognition in AD. Given the observation of sex-specific outcomes in previous studies (Cuervo-Zanatta et al., 2021; Dunham et al., 2024; Carroll et al., 2010; Farkas et al., 2022; Quinn et al., 2022; Carroll et al., 2007; Kapadia et al., 2018; Dennison et al., 2021; Manji et al., 2019), we investigated both male and female mice. We found that adaptive immune responses were elevated in multiple tissues and varied by sex and microbiome status. Specifically, we show that the time of onset of the adaptive immune response in CNS-draining lymph nodes is sex-dependent, occurring earlier in female mice. In the 3xTg model, immune responses were dramatically elevated when males, but not females, were kept germ-free. In contrast, the 5xFAD model showed increased immune activity in females compared to males in SPF conditions. Importantly, the absence of a microbiome attenuated immune responses and improved cognitive function, primarily in female mice in both models. Our results demonstrate that the microbiome and sex interact to influence immunity and cognition in mouse models of AD.

## 2. Materials and methods

### 2.1. Mice

Wildtype C57BL/6J (The Jackson Laboratory, Cat#000664) mice were obtained from The Jackson Laboratory at 8 weeks of age. Homozygous 3xTg [B6;129-Tg(APPSwe,tauP301L)1Lfa *Psen1*^*tm1Mpm*^/Mmjax] and hemizygous 5xFAD [B6.Cg-Tg(APPSwFlLon,PSEN1*M146L*L28 6V)6799Vas/Mmjax] (The Jackson Laboratory, Cat#004807 and Cat#034848) mice were maintained in a colony in the laboratory of S.K. M at the California Institute of Technology (Caltech). 5xFAD hemizygous mice and wildtype littermates were produced by crossing transgenic and C57BL/6J mice. C57BL/6J, 3xTg, and 5xFAD mice were rederived as germ-free (GF) in the Caltech gnotobiotic facility. All transgenic mouse lines used in this study were maintained on a C57BL/6J background and were backcrossed for more than ten generations to ensure genetic consistency. Wildtype and mutant mice were not co-housed prior to GF derivation. Both strains were rederived GF through c-section. At E19 the SPF donor was euthanized and the uterus with pups surgically removed and submerged in Virkon. The uterus was placed in the transfer port of the re-derivation isolator and the port fogged with Clidox. The uterus was transferred into the isolator, then the pups were removed from the uterus and stimulated until breathing on their own. They were then placed with a GF foster dam. The pups and chamber were tested for a minimum of 4 weeks to confirm germ-free status. All isolators were tested bi-weekly with anaerobic/aerobic culture on plates and 16S PCR. After weaning, mice were housed together with littermates until experiments. All experiments were performed with both female and male mice. For behavioral experiments, investigators were not blinded to group.

Germ-free experimental mice were housed in sterilized microisolator cages and maintained on ad libitum autoclaved 5010 PicoLab Rodent Diet (LabDiet, Cat#5010) and sterilized water. Germ-free mice were maintained on water containing antibiotics during behavioral tests [ampicillin (1 mg/mL); vancomycin (0.5 mg/mL); neomycin (1 mg/mL); 1 % sucrose]. Specific pathogen-free (SPF) control mice were maintained on ad libitum irradiated 5053 PicoLab Rodent Diet (LabDiet, Cat#5053) and provided either sterilized water or sterilized water containing 1 % sucrose during behavioral tests. Animals were group housed (2–5 mice per cage) unless otherwise specified. Conditions in the animal housing facilities were maintained at 21–24 °C, 30–70 % humidity, with a cycle of 13 h light, 11 h dark. All experiments were performed with approval from the Institutional Animal Care and Use Committee (IACUC) of Caltech (Protocol IA20–1798).

### 2.2. Immune cell isolation and profiling

#### 2.2.1. Intestinal lamina propria immune cell isolation

For isolation of intestinal lamina propria cells, the small and large intestines were dissected and placed immediately into ice-cold phosphate-buffered saline (PBS). After mesenteric fat and Peyer’s patches (small intestine) were removed, the intestines were longitudinally opened, and luminal contents were washed out with cold PBS. Tissue pieces were washed for 10 min. in 1 mM dithiothreitol (DTT)/PBS at room temperature on a rocker to remove mucus, followed by a wash for 25 min. in 10 mM ethylenediaminetetraacetic acid (EDTA)/30 mM 4-(2-hydroxyethyl)-1-piperazineethanesulfonic acid (HEPES)/PBS at 37 °C on a platform shaker (180 rpm) to remove epithelium. After a 2 min. wash in complete RPMI, the tissue was digested in a six-well plate for 1.5 hr. in complete RPMI with 150 U/mL (small intestine) or 300 U/mL (large intestine) collagenase VIII (Sigma-Aldrich) and 150 μg/mL DNase (Sigma-Aldrich) in a cell culture incubator (5 % CO_2_). Tissue digests were passed through a 100 μm cell strainer and separated by centrifugation (1250×*g* for 20 min.) using a 40/80 % Percoll gradient. Immune cells were collected at the 40/80 % interface and washed with Hanks’ balanced salt solution (HBSS) wash buffer [1X HBSS without phenol red, Ca^2+^, or Mg^2+^; 10 mM HEPES buffer, DNase I (50 μg/ml), 1 mM MgCl_2_, and bovine serum albumin (BSA) Fraction V (2.5 mg/ml)] before surface staining, fixation (eBioscience Foxp3 / Transcription Factor Staining Buffer Set), and intracellular staining.

#### 2.2.2. Spleen and lymph node immune cell isolation

For the spleen and lymph nodes, the tissue was passed through a 100 μm cell strainer and washed with HBSS wash buffer. The spleen suspension was centrifuged at 500 × g for 5 min., resuspended in red blood cell lysis buffer (Sigma-Aldrich), and incubated for 8 min. at room temperature. Spleen and lymph node cell suspensions were washed with 0.5 % BSA/PBS before surface staining and fixation (eBioscience Foxp3 / Transcription Factor Staining Buffer Set).

#### 2.2.3. Cell counting and normalization

Single cell suspensions of tissues were washed and cells resuspended in 1 mL 0.5 % BSA/PBS. 10 μL of this volume was diluted in 190 μL 0.5 % BSA/PBS buffer. Flow cytometry was performed on a Beckman Coulter CytoFLEX S flow cytometer. Each sample was run for 30 s at a set flow rate of 60 μL/min., so that 30 μL was used for cell counting. To calculate cells counts, FlowJo was utilized to gate cells and exclude potential debris. The gated cell count was used to calculate the total cells for each tissue with the following formula:

$$Totalcellcount=Gatedcellcount\times \frac{startvol.}{samplevol.}\times \frac{samplevol.}{runvol.}$$

$$=Gatedcellcount\times \frac{1,000\mu L}{10\mu L}\times \frac{200\mu L}{30\mu L}$$

=Gatedcellcount×666.67

#### 2.2.4. Immune cell profiling by flow cytometry

CD16/32 antibody (eBioscience) was used to block non-specific binding to Fc receptors before surface staining at 4 °C for 30 min. Immune cells were stained with antibodies (eBioscience, BioLegend) for surface markers (1:200 dilution): CD19 (FITC), CD3e (PE), CD4 (APC; BV510), CD45.2 (BV421), CD8a (APC-e780), and TCRβ (PerCP-Cy5.5) and intracellular markers (1:100 dilution): Foxp3 (APC), GM-CSF (PE), IFNγ (FITC), IL-17A (PE), and IL-4 (PE-Cy7). Live and dead cells were discriminated by Live/Dead Fixable Violet or Aqua Dead Cell Stain Kit (Invitrogen). Cells were stimulated with 50 ng/ml phorbol-12-myristate-13-acetate (PMA) (Sigma, Cat#79346) and 500 ng/ml ionomycin (Sigma, Cat#I0634) in RPMI 1640 complete medium (supplemented with 10 % fetal bovine serum (FBS), 2 mM GlutaMAX, 55 μM 2-mercaptoethanol, 10 mM HEPES, 100 U/ml penicillin and 100 μg/ml streptomycin) for 4 h and 2 μg/ml Brefeldin A (Sigma, Cat#B6542) was added 2 h before cell collection. Mean fluorescence intensity (MFI) was calculated as the median value of the channel gated on the positive cell population. Samples with <1000 total cells were excluded from analysis. MFI was calculated for samples with >10 positive cells. Summary tables for key immune response data and representative plots for all immune measurements are provided in the supplementary material.

### 2.3. Behavior testing

Noldus EthoVision software (Wageningen, Netherlands) was used to record and analyze animal behavior. Animals were brought into the behavior testing room a half-hour prior to testing for acclimation to the room between 9 am and 10:30 am. Behavior testing occurred immediately after habituation in the mornings. Temporary housing was directly across the hall in the animal facility, so the animals experienced minimal movement on test days. All behavior and temporary housing room conditions were in accordance with IACUC guidelines (light cycle, sound level, temperature, and humidity).

#### 2.3.1. Y maze

Spatial memory was assessed by spontaneous alternation measurement in a three-arm open maze as described previously (Prieur and Jadavji, 2019). Mice were acclimated in the behavior room for thirty minutes prior to testing. Each arm was assigned a letter (A, B, or C). Mice were placed into Arm C and then allowed a period of five minutes to explore the maze. At the end of five minutes, the mouse was removed from the maze. The maze was thoroughly cleaned with 0.5 % hydrogen peroxide disinfectant and allowed to dry to remove olfactory cues. A camera mounted above the center of the maze was utilized to identify the center point of each mouse. Arm entries and re-entries were counted automatically with EthoVision software. An arm entry was counted if the center point of the mouse crossed the threshold between the arm and center. If a mouse entered an arm momentarily, returned to the center of the maze, and then entered the same arm again, this was considered as two entries to the same arm. Spontaneous alternations were classified as successive entries into three successive arms without error. Percent alternations was calculated with the following equation:

$$\%Alternations=\left(\frac{spontaneousalternations}{totalarmentries-2}\right)\;\;\times \;\;100$$

#### 2.3.2. Novel object recognition

Mice were moved into the behavior room thirty minutes prior to assessment on day one to acclimate. Mice were assessed over two days and housed overnight in the behavior room between assessment days. Mice were placed into a box with two similar objects, either two 8 x 4 in. colored blocks, or two cell culture flasks filled with sand, and allowed to explore the box for ten minutes as described previously (Leger et al., 2013). The initial objects were referred to as the “familiar objects.” Half the mice were familiarized to colored blocks and half to flasks to avoid object bias. The following day, one of the familiar objects was replaced with a “novel object” of the opposite type and the mice were allowed to explore the field and objects for an additional ten minutes. Time spent at each object was recorded with EthoVision software. The following formula was used to measure novel object recognition:

$$RecognitionIndex=\frac{timeatnovelobject}{timeatfamiliarobject+timeatnovelobject}$$

#### 2.3.3. Barnes maze

We followed the shortened Barnes Maze protocol outlined in Attar et al. (Attar et al., 2013). The procedure included a habituation period, four training periods, and a final test period. All mice were habituated for a minimum of thirty minutes in the behavior room to allow for adjustment to their surroundings. We maintained a minimum of one hour between the habituation period and training periods. Each cohort completed a habituation period and two training periods on day one. Two more training sessions were completed on day two. Mice were rested on day three and tested on day four. The Barnes Maze has eighteen equally spaced holes around the perimeter of a circular maze. The goal box was always aligned in the same position relative to visual cues in the room. Four reference objects, in addition to the natural asymmetry of the behavior room, were used during all periods of testing to orient the mice in the room. In the habituation phase, mice were guided to a dark goal box located beneath the target hole. Aversive stimuli, including bright diffuse light, fan-induced air currents, and white noise, provided encouragement for the mice to find the goal box during the trainings and test. During each training, mice were allowed 180 s to locate and enter the goal box. When the mouse entered the goal box, the aversive noise machine was turned off via remote control. Mice were allowed twenty seconds to further acclimate to the goal box before being returned to their home cage. If the mouse did not enter the goal box on their own within 180 s, they were guided to the goal box and allowed to acclimate for twenty seconds without the aversive noise. They were then returned to their home cage. On the test day, the goal box was removed, and the mice were allowed to roam for 120 s. We assessed the latency in seconds required for mice to reach the goal box during the trainings or the location of the removed goal box on test day.

### 2.4. Statistical methods

Unless otherwise noted, statistical analyses were performed with the non-parametric Mann-Whitney *U* test for behavior assays with non-Gaussian distributions and two-way ANOVA for immune response data on individual biological samples with GraphPad Prism 10.0. Two-way ANOVA analyses were corrected for multiple comparisons with Tukey’s multiple comparisons test. Using G*Power analysis based on variability in the primary immune outcomes (e.g., frequency of cytokine^+^ CD4^+^ T cells), the sample sizes in this study provided 80 % power to detect effect sizes of 0.48. Unless otherwise noted, only statistical comparisons with P < 0.05 are shown. *P < 0.05, **P < 0.01, ***P < 0.001, ****P < 0.0001, n.s. not significant.

## 3. Results

### 3.1. Adaptive immunity is enhanced in early life and differentially by sex in mouse models of AD

**Fig. 1.**
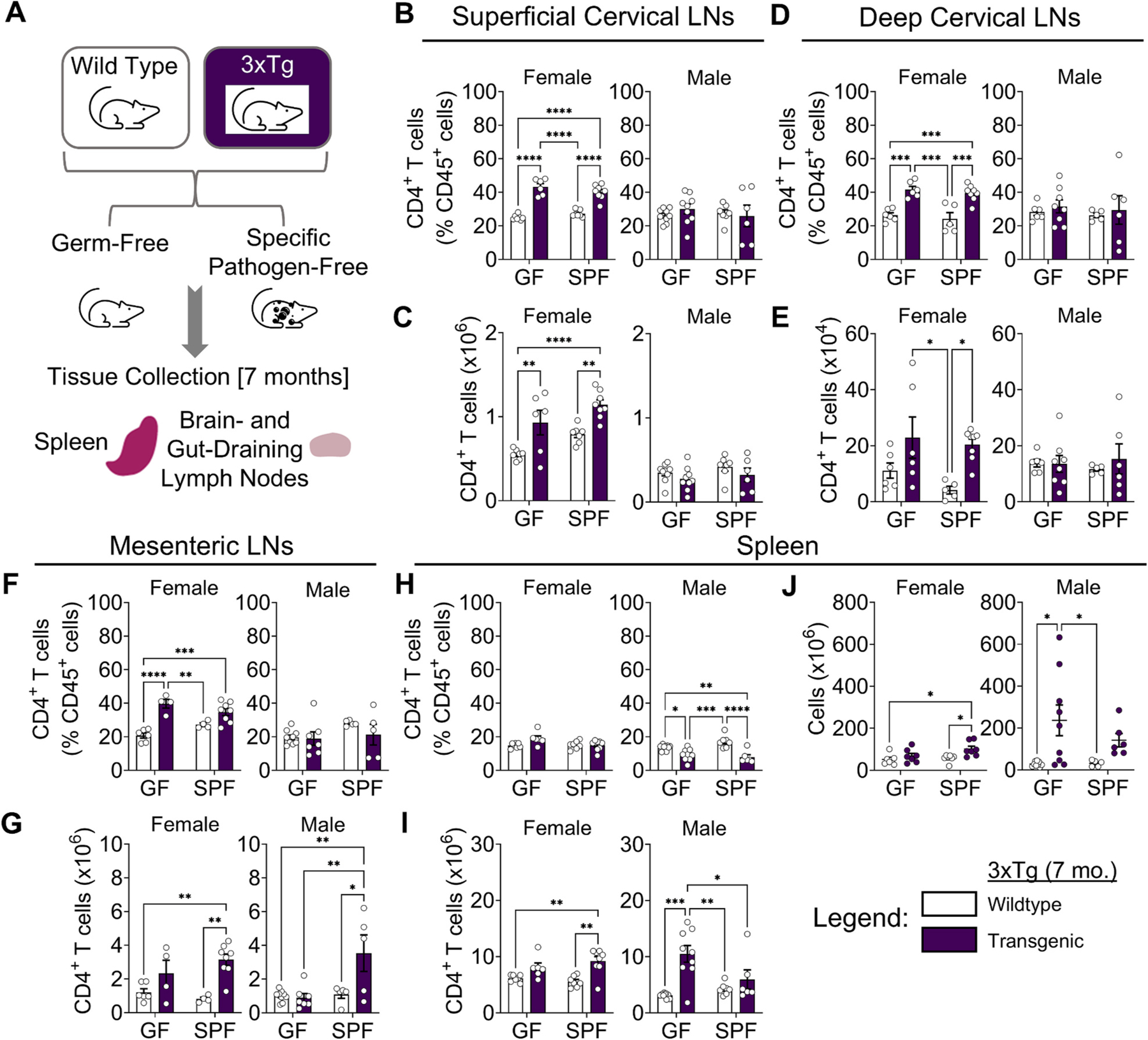
Adaptive immunity is activated early and differentially by sex in the 3xTg model of AD. (A) Illustration of the experimental design for immune response analysis. (B) Quantification of CD4^+^ T cell frequencies and (C) counts in the superficial cervical lymph nodes of female (Left) and male (Right) 3xTg mice compared to wildtype controls in germ-free (GF) and specific pathogen-free (SPF) conditions at 7 months of age. (D) Frequencies and (E) cell counts for deep cervical lymph nodes. (F) Frequencies and (G) cell counts for mesenteric lymph nodes. (H) Frequencies and (I) cell counts for spleen. (J) Total spleen cell counts. Data are pooled from 2 independent experiments (n = 4 to 9 per group); two-way ANOVA. Error bars indicate SEM. *P < 0.05, **P < 0.01, ***P < 0.001, ****P < 0.0001.

To evaluate the contribution of the microbiome to immune responses in Alzheimer’s disease (AD), we examined immune responses in two well-established mouse models (3xTg and 5xFAD) (Oddo et al., 2003; Oakley et al., 2006) in the presence or absence of the microbiome, utilizing specific pathogen-free (SPF; i.e., standard laboratory microbiota) and germ-free (GF) mice (Fig. S1; Fig. 1A). We analyzed both local (tissue-specific draining lymph nodes) and systemic (spleen) immune responses in male and female mice at ages corresponding to previously reported onset and progression of AD-related pathology: 7, 12, and 15 months for the 3xTg model and 5 and 8 months for the 5xFAD model.

In the superficial and deep cervical lymph nodes (CLNs), which are the primary draining lymph nodes for the lymphatics associated with the brain, head, and neck, we found that CD4^+^ T cell frequencies and numbers were elevated in female 3xTg mice at the earliest age measured (7 months), whether the mice were raised under GF or SPF conditions (Fig. 1B–E). This increase in CD4^+^ T cells in CNS-associated lymph nodes occurred before the onset of behavioral deficits previously reported at 12 months of age (Carroll et al., 2010; Creighton et al., 2019). T cell count increases in the CLNs were primarily driven by CD4^+^ (helper) T cells (Fig. 1C and 1E). CD8^+^ (cytotoxic) T cell counts were decreased in the 3xTg CLNs, with the exception of the deep CLNs of female 3xTg mice (Fig. S2A and B). Notably, there were no significant increases in CD4^+^ or CD8^+^ T cell frequencies in the CLNs of male 3xTg mice at the same age, indicating sexual dimorphism in immune responses in the 3xTg model at this early age (Fig. 1B–E; Fig. S2A and B). This parallels previously described differences in the onset of Aβ plaque accumulation in the brains of 3xTg mice (Javonillo et al., 2022; Carroll et al., 2010; Dennison et al., 2021), with female mice showing plaques in the hippocampus and cortex and impaired cognitive function at earlier ages than males (Javonillo et al., 2022; Brigas et al., 2021). We found decreased B cell numbers in the superficial CLNs of GF and SPF female 3xTg mice (Fig. S3A), indicating differential rates of accumulation or egress between T and B cells in the lymph nodes. No significant changes in total B cell numbers were observed in the deep cervical lymph nodes between SPF and GF mice of either sex or genotype (Fig. S3B).

In the mesenteric lymph nodes (MLNs), which drain the small and large intestines, we observed elevated CD4^+^ T and B cell numbers in both SPF female and male 3xTg mice at 7 months of age, but not in GF mice, indicating increased adaptive immune response in the intestines that is microbiome dependent (Figs. 1G and S3C). CD4^+^ T cell frequencies in the MLNs increased in both GF and SPF female, but not male, mice (Fig. 1F). CD8^+^ T cell numbers in the MLNs were unaffected by sex, genotype, or microbiome status (Fig. S2C).

We next assessed systemic immune responses by examining the spleen. CD4^+^ T cell frequencies were decreased in male, but not female, 3xTg mice under both microbiome conditions (Fig. 1H). However, total CD4^+^ T cell numbers were elevated in both female and male 3xTg mice, with the greatest increase in GF male mice (Fig. 1I). CD8^+^ T cell counts in the spleen were unaffected by sex, genotype, or microbiome status (Fig. S2D). The increased CD4^+^ T cell numbers tracked with an increased overall number of cells in the spleen, consistent with previous observations of increased immune cell numbers in the spleen in this model (Fig. 1J) (Marchese et al., 2014; Yang et al., 2015). Although not statistically significant, the spleen cell counts trended higher in GF compared to SPF male 3xTg mice (Fig. 1J). The greater increase in spleen cellularity in GF male mice was unexpected and suggests a sex-dependent role for the microbiome in systemic immune responses in 3xTg mice.

**Fig. 2.**
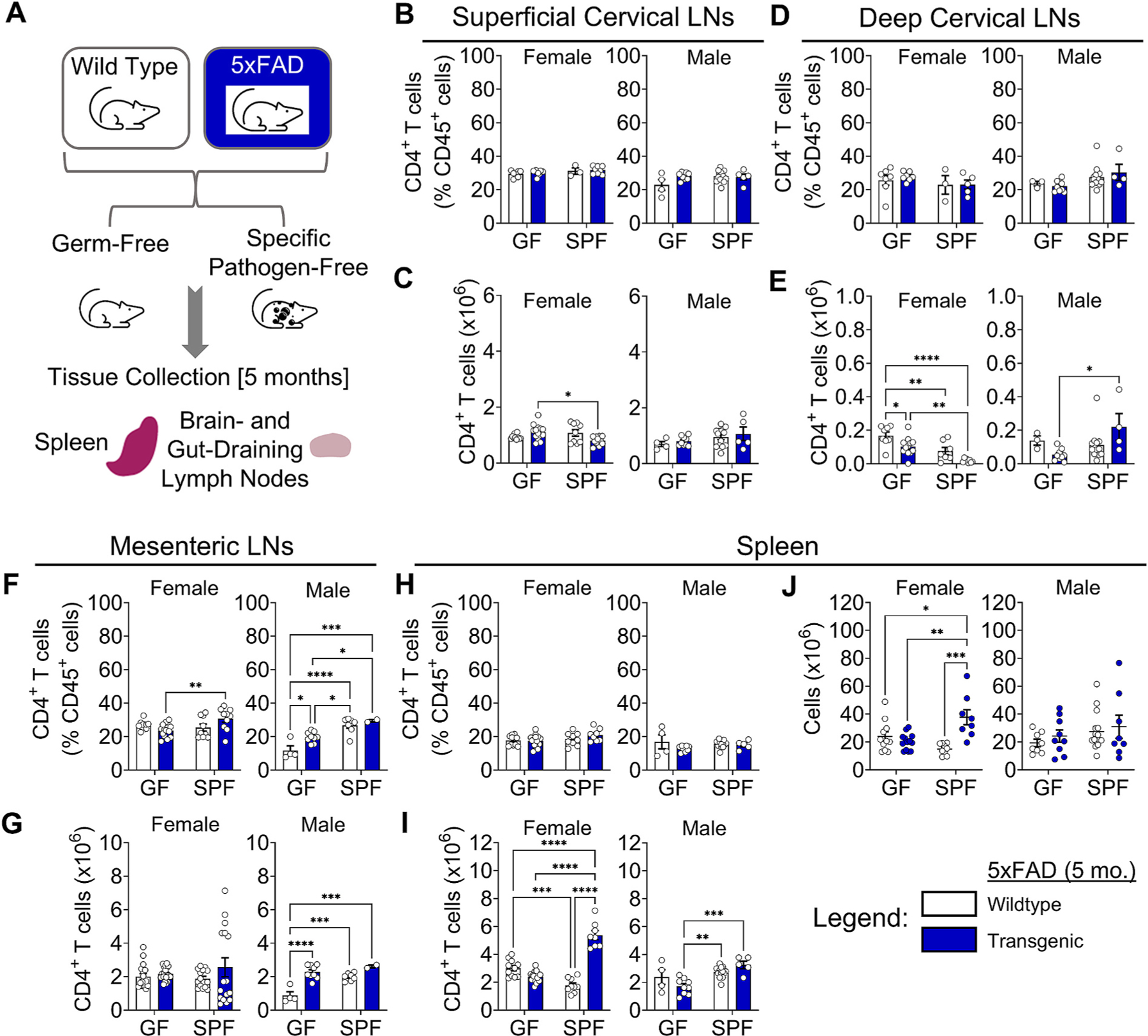
Adaptive immunity in the spleen is altered differentially by sex in the 5xFAD model of AD. (A) Illustration of the experimental design for immune response analysis. (B) Quantification of CD4^+^ T cell frequencies and (C) counts in the superficial cervical lymph nodes of female (Left) and male (Right) 5xFAD mice compared to wildtype controls in germ-free (GF) and specific pathogen-free (SPF) conditions at 5 months of age. (D) Frequencies and (E) cell counts for deep cervical lymph nodes. (F) Frequencies and (G) cell counts for mesenteric lymph nodes. (H) Frequencies and (I) cell counts for spleen. (J) Total spleen cell counts. Data are pooled from 2 independent experiments (n = 4 to 16 per group); two-way ANOVA. Error bars indicate SEM. *P < 0.05, **P < 0.01, ***P < 0.001, ****P < 0.0001.

In contrast to the 3xTg mouse model, we observed no changes in CD4^+^ or CD8^+^ T cell frequencies in the CLNs in female or male 5xFAD mice in either GF or SPF conditions at the earliest age measured (5 months) (Fig. 2A, B and D; Fig. S2E and F). However, decreases in total CD4^+^ T cell (Fig. 2E) and B cell (Fig. S3F) numbers were observed in the deep CLNs, while cell numbers in the superficial CLNs remained unchanged (Fig. 2C; Fig. S3E).

In the MLNs of 5xFAD mice at 5 months of age, a slight increase in CD4^+^ T cell frequency was observed between GF and SPF conditions in transgenic mice (Fig. 2F), although total CD4^+^ and CD8^+^ T cell numbers were largely unchanged (Fig. 2G; Fig. S2G). B cell numbers were elevated in female 5xFAD mice under GF compared to SPF conditions (Fig. S3G). Together, these data indicated increased adaptive immune response in the intestines that is microbiome dependent.

In the spleen, 5xFAD mice showed no changes in CD4^+^ T cell frequencies (Fig. 2H), but significant increases in CD4^+^ (Fig. 2I), CD8^+^ (Fig. S2H), T cell, and B cell (Fig. S3H) numbers, an effect which was most pronounced in SPF female mice. This corresponded to an increase in cell numbers in the spleens of SPF 5xFAD female mice (Fig. 2J).

Together, these results show elevated adaptive immune responses both in locally draining lymph nodes (CNS and intestine) and systemically (spleen) at the earliest ages examined in the 3xTg model. There was little change in adaptive immune responses in 5xFAD mice at 5 months, except in the MLNs and spleen. In 3xTg mice, CD4^+^ T cell numbers in CLNs were elevated in females, but not males, regardless of microbiome status. In contrast to the CNS-associated T and B cell responses that were independent of the microbiome, systemic T and B cell responses in 3xTg mice were modulated by the microbiome and differed by sex, with increased immune cell numbers in GF males. In 5xFAD mice, increases in T and B cell numbers were restricted to the spleen and MLNs and dependent on the microbiome, suggesting less pronounced immune responses than in 3xTg mice. These data reveal sexual dimorphism in both immunity and the effects of the microbiome in these mouse models. It is not clear whether the elevated CD4^+^ T cells and B cells play protective or pathogenic roles.

### 3.2. The immune response in 3xTg mice is characterized by increased cytokine responses in males, but attenuated cytokine responses in females

**Fig. 3.**
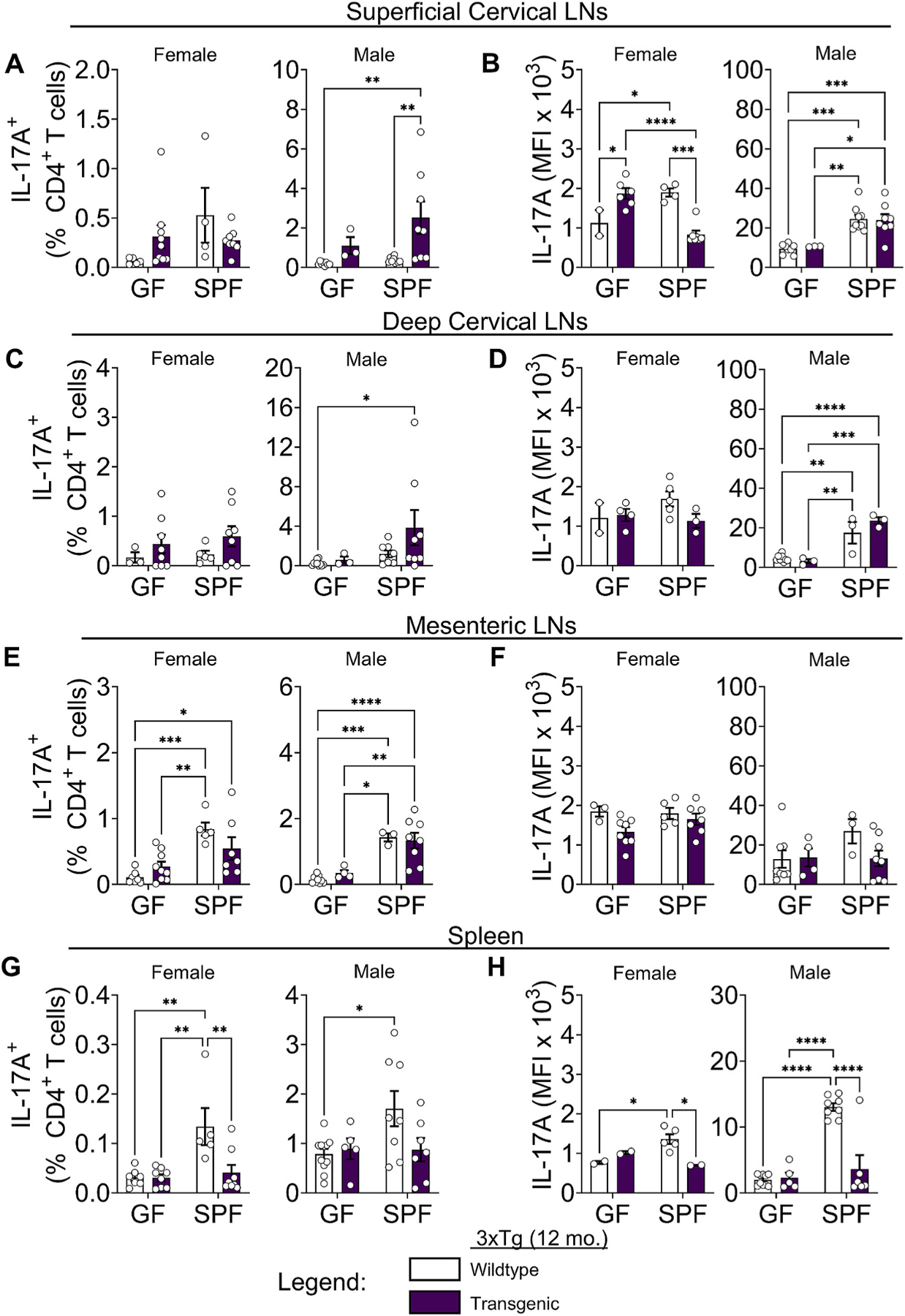
The immune response in 3xTg mice is characterized by increased cytokine responses in males, but attenuated cytokine responses in females. (A) Quantification of IL-17A^+^ T cell frequencies and (B) IL-17A mean fluorescence intensity (MFI) in the superficial cervical lymph nodes of female (Left) and male (Right) 3xTg mice compared to wildtype controls in germ-free (GF) and specific pathogen-free (SPF) conditions at 12 months of age. (C) Frequencies and (D) MFI for deep cervical lymph nodes. (E) Frequencies and (F) MFI for mesenteric lymph nodes. (G) Frequencies and (H) MFI for spleen. Data are pooled from 2 independent experiments (n = 3 to 10 per group); two-way ANOVA. Cells were stimulated using phorbol 12-myristate 13-acetate (PMA) and ionomycin in the presence of Brefeldin A prior to intracellular cytokine staining. Error bars indicate SEM. *P < 0.05, **P < 0.01, ***P < 0.001, ****P < 0.0001.

To characterize the quality of T cell responses, we examined cytokine production and profiled effector and regulatory T cells in both the 3xTg and 5xFAD models. The cytokine interleukin (IL)-17A has been implicated in the pathogenesis of neurodegenerative diseases including stroke, multiple sclerosis, AD, and Parkinson’s disease (Mills, 2023). IL-17A is expressed at barrier and mucosal surfaces, including the skin, gastrointestinal and urogenital tracts, and is induced by specific bacterial taxa of the microbiome that colonize environmentally exposed surfaces of the body (Mills, 2023; Pandiyan et al., 2019). The functional role of IL-17A in the CNS is under active investigation, but data suggest that this cytokine promotes neuroinflammation (Brigas et al., 2021; Mills, 2023; McGinley et al., 2020; Benakis et al., 2016). In 12-month-old 3xTg mice, we observed elevated IL-17A^+^ CD4^+^ T cells in the CNS-associated superficial and deep CLNs (Fig. 3A and C). The increase in IL-17A^+^ CD4^+^ T cells was statistically significant in male mice, despite unchanged overall CD4^+^ T cell frequencies in the CLNs (Fig. S4C and D). Both IL-17A^+^ T cell frequencies (Fig. 3A, C, E and G), and IL-17A mean fluorescence intensity (MFI; Fig. 3B, D, F and H) — a measurement of average cytokine production per cell — trended higher in SPF compared to GF conditions, consistent with IL-17 induction by the microbiome. Notably, IL-17A MFI in female mice was lower compared to male mice (Fig. 3B, D, F and H), indicating low IL-17A production by T cells in female mice. In peripheral lymphoid tissues outside of the CNS, such as the spleen and MLNs, there were decreased frequencies of IL-17A-producing T cells and lower MFI in both male and female SPF 3xTg compared to SPF wildtype mice (Fig. 3E–H). Overall, 3xTg mice had elevated, but not statistically significant, increases in the frequency of IL-17A-producing T cells compared to wildtype controls (Fig. 3A, C, E and G), despite showing elevated CD4^+^ T cell frequencies in the lymph nodes that were microbiome dependent (Fig. S4A–D).

Interferon gamma (IFNγ) is a signature cytokine in neuroinflammatory environments (Becher et al., 2017). Similar to IL-17A, IFNγ-producing T cells and IFNγ MFI were increased in male, but not female 3xTg mice, particularly in the superficial and deep CLNs (Fig. S5A–D). IFNγ-producing T cells were elevated in MLNs of male 3xTg mice, although not statistically significantly (Fig. S5E). In 3xTg female mice, IFNγ MFI decreased significantly between GF and SPF conditions (Fig. S5B, D, F, and H). This was not observed in male mice, indicating a sex-dependent microbiome effect on IFNγ MFI. Notably, in SPF female mice, IFNγ MFI decreased comparing wildtype to transgenic mice, suggesting a sex-dependent regulation of cytokine production (Fig. S5B, D, F, and H). In the spleen, we observed decreased frequencies of IFNγ-producing T cells and lower MFI in both male and female SPF 3xTg compared to SPF wildtype mice (Fig. S5G and H). These results are congruent with the IL-17A results and show an increase in IFNγ^+^ T cell frequencies in the CLNs of male 3xTg mice, but a decrease in IFNγ^+^ T cell frequencies and cytokine production in the spleens of female and male 3xTg SPF mice, highlighting differences between local and systemic immune responses and effects of the microbiome.

In inflamed environments, regulatory T cell numbers increase with expanding cytokine-producing effector T cells and act to limit immune responses (Smigiel et al., 2014; Sakaguchi et al., 2008). A major subset of CD4^+^ regulatory T cells is marked by the forkhead box transcription factor P3 (Foxp3). Foxp3^+^ T cell frequencies were not significantly changed between transgenic and WT mice but were reduced in SPF compared to GF 3xTg mice in most tissues and both sexes (Fig. S6A–D). This indicates a potentially unbalanced regulatory response by Foxp3^+^ T cells in 3xTg mice.

### 3.3. The immune response in 5xFAD mice is characterized by increased IL-17A-producing T cells

**Fig. 4.**
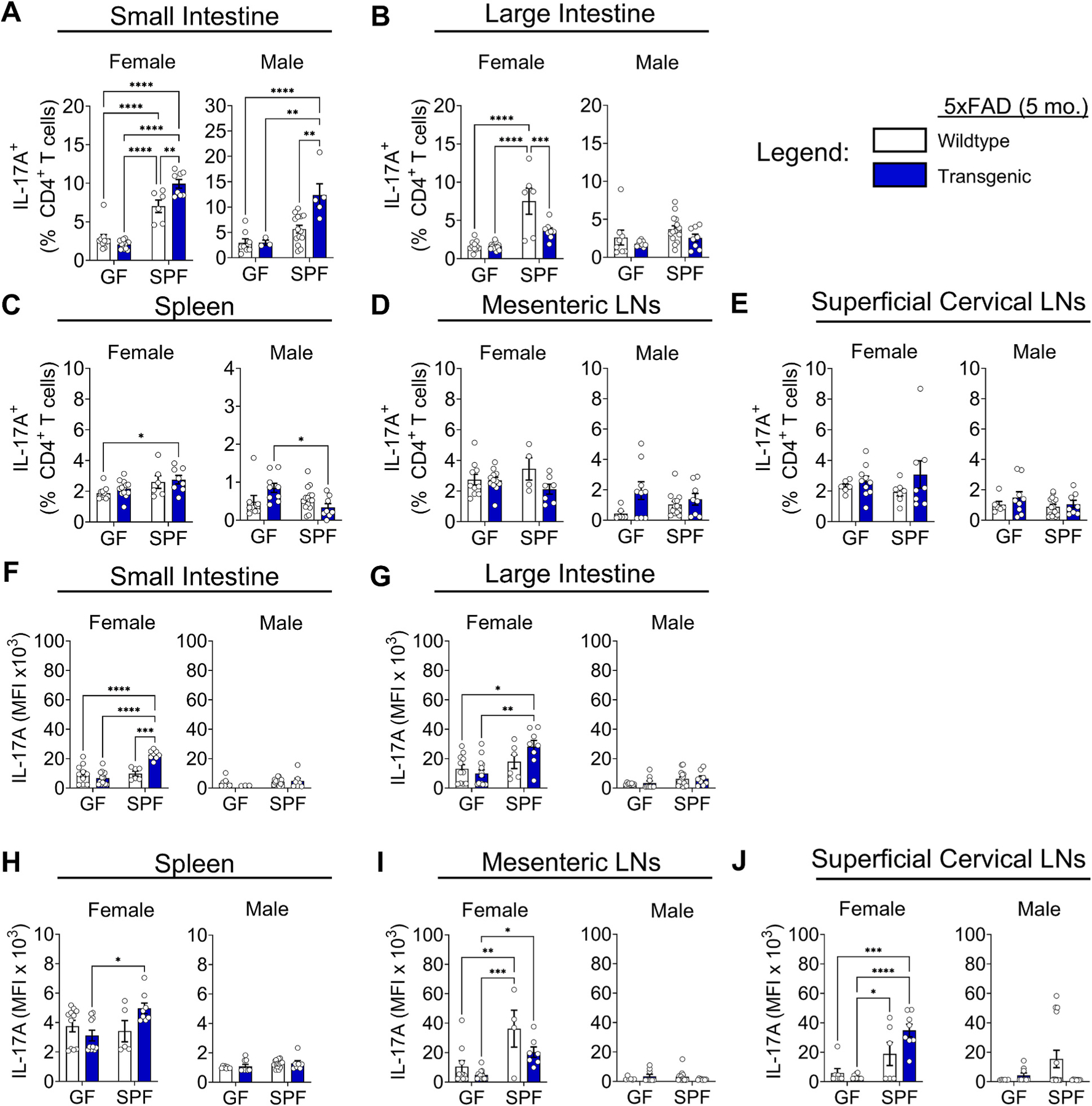
The immune response in 5xFAD mice is characterized by increased IL-17A-producing T cells. (A) Quantification of IL-17A^+^ T cell frequencies in the small intestines of female (Left) and male (Right) 5xFAD mice compared to wildtype controls in germ-free (GF) and specific pathogen-free (SPF) conditions at 5 months of age. (B) Large intestine. (C) Spleen. (D) Mesenteric lymph nodes. (E) Superficial cervical lymph nodes. (F) Quantification of IL-17A mean fluorescence intensity (MFI) expressed by CD4^+^ T cells in the small intestine. (G) Large intestine. (H) Spleen. (I) Mesenteric lymph nodes. (J) Superficial cervical lymph nodes. Data are pooled from 2 independent experiments (n = 4 to 14 per group); two-way ANOVA. Cells were stimulated using PMA (phorbol 12-myristate 13-acetate) and ionomycin in the presence of Brefeldin A prior to intracellular cytokine staining. Error bars indicate SEM. *P < 0.05, **P < 0.01, ***P < 0.001, ****P < 0.0001.

Consistent with the role of the microbiome in promoting IL-17A production (Mills, 2023; Pandiyan et al., 2019), we found that IL-17A^+^ T cells and IL-17A MFI in the intestines of 5xFAD mice were increased by the presence of the microbiome (i.e., SPF conditions) (Fig. 4A, B, F and G). Importantly, and unexpectedly, we found IL-17A-producing CD4^+^ T cells were further increased in the small intestines of SPF 5xFAD transgenic female and male mice compared to wildtype controls at 5 months of age (Fig. 4A). Previous reports indicated reduced IL-17A production in the gut-associated immune cell populations of 5xFAD transgenic mice, specifically in the mesenteric lymph nodes and Peyer’s patches (Saksida et al., 2017). Indeed, we observed lower IL-17A^+^ T cell frequencies in the large intestine, spleen, and MLNs of SPF transgenic mice (Fig. 4B–D) and lower IL-17A MFI in the MLNs (Fig. 4H and I). We found no changes in IL-17A^+^ T cell frequencies or counts in the superficial CLNs (Figs. 4E and S7A), however, we observed an increase in IL-17A MFI in SPF transgenic female mice (Fig. 4J). The increase in the IL-17A^+^ T cell populations in the small intestine tissue (lamina propria) (Fig. 4A) vs. the large intestine and MLNs (Fig. 4B and D) indicates the environment of the small intestine is specifically conducive to either IL-17A^+^ T cell differentiation, proliferation, and/or retention. In accordance with the observed increase in spleen cell counts (see Fig. 2J), the total number of IL-17A^+^ T cells dramatically increased in the spleens of SPF female 5xFAD mice (Fig. S7B). We observed a moderate increase in IL-17A^+^ T cell numbers in the MLNs of GF male transgenic mice (Fig. S7C). The observed increase of IL-17A^+^ T cells in the 5xFAD transgenic mice suggests increased cytokine production that is modulated by gut bacteria (McGinley et al., 2020; Benakis et al., 2016) and may be induced by an alteration in microbiome localization or composition in the intestines, or both.

We observed largely unchanged IFNγ-producing T cell frequencies in 5xFAD compared to wildtype control mice in most tissues (Fig. S8A–E). However, IFNγ^+^ T cell frequencies increased in the spleen and superficial CLNs of female SPF 5xFAD mice (Fig. S8C and E), consistent with a previous study that noted increased transcription of the IFNγ-induced chemokine Cxcl10 in the brains of female 5xFAD mice at 5 months of age (Manji et al., 2019). IFNγ MFI remained unchanged in most tissues (Fig. S8F–G), apart from an increase in the MLNs and superficial CLNs of SPF female mice (Fig. S8I and J).

We examined two additional cytokines associated with activated immune responses, granulocyte–macrophage colony-stimulating factor (GM-CSF) and interleukin (IL)-4. GM-CSF produced by lymphocytes activates myeloid cells, such as macrophages and microglia, and participates in the communication between the two subsets of immune cells (Becher et al., 2017). IL-4 is a key cytokine in the induction of Type 2 helper T cells (Th2) that mediate allergy, asthma, and wound repair, and in addition, acts as an anti-inflammatory cytokine that antagonizes Type 1 (IFNγ) immune responses (Li-Weber and Krammer, 2003). Although only statistically significant in the small intestine, we observed an overall trend of increased GM-CSF^+^ T cell frequencies in GF 5xFAD transgenic mice compared to GF wildtype controls, but decreased frequencies in SPF mice (Fig. S9A–E). GM-CSF MFI was significantly increased in the small intestine, MLNs, and superficial CLNs of SPF compared to GF female mice, but not in other tissues or male mice (Fig. S9F–J). We generally observed low and highly variable frequencies of IL-4^+^ T cells in all tissues examined (Fig. S10A–E). Remarkably, IL-4 MFI was increased in all tissues examined in SPF transgenic compared to SPF wildtype female and male mice, but not in GF mice, possibly indicating a regulatory response to Type 1 immunity (Fig. S10F–J).

We observed increased Foxp3^+^ regulatory T cell frequencies and counts in the spleens, MLNs and deep CLNs of SPF female, but not male, 5xFAD mice (Fig. S11A, B, and D). Male mice showed lower proportions of Foxp3^+^ T cells in the MLNs, superficial and deep CLNs, and large, but not small, intestine, comparing SPF to GF conditions (Fig. S11B–F). Together, these data indicate increased immune responses that are balanced by the induction of regulatory T cells in female, but not male, 5xFAD mice.

### 3.4. Longitudinal immune responses differ between 3xTg mice and 5xFAD mice

**Fig. 5.**
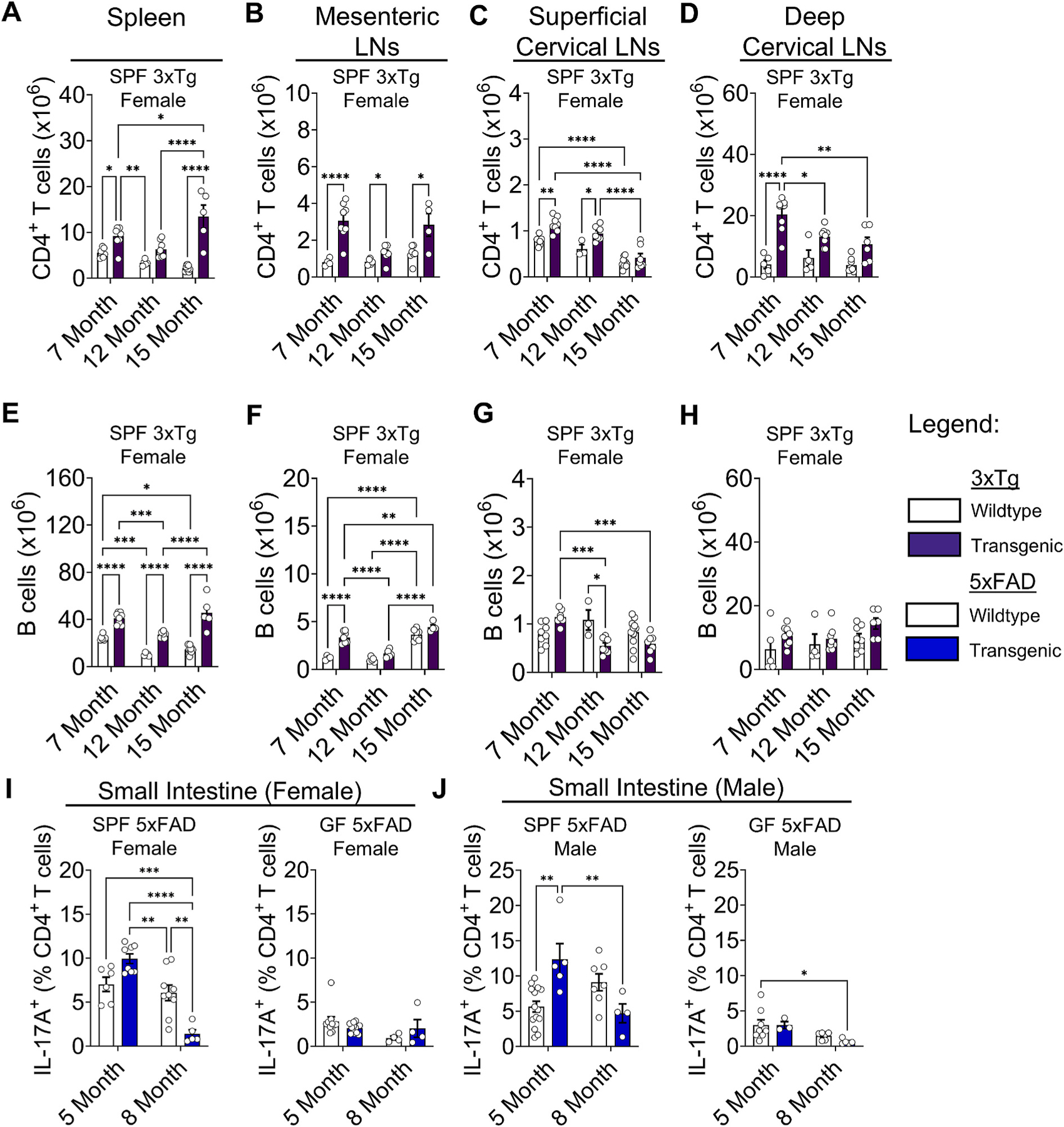
Longitudinal immune responses differ between 3xTg mice and 5xFAD mice. (A) Quantification of CD4^+^ T cell counts in the spleens of female 3xTg mice - compared to wildtype controls in specific pathogen-free (SPF) conditions at 7-, 12-, and 15-month ages. (B) Mesenteric lymph nodes. (C) Superficial cervical lymph nodes. (D) Deep cervical lymph nodes. Data are pooled from 5 independent experiments (n = 3 to 8 per group); two-way ANOVA. (E) Quantification of B cell counts in the spleens of female 3xTg mice. (F) Mesenteric lymph nodes. (G) Superficial cervical lymph nodes. (H) Deep cervical lymph nodes. Data are pooled from 5 independent experiments (n = 3 to 11 per group); two-way ANOVA. For clarity, only significant comparisons of the same mouse group between ages or between transgenic and control mice at the same age are shown. (I) Quantification of IL-17A^+^ T cell frequencies in the small intestines of female 5xFAD mice compared to wildtype controls in SPF (Left) and GF (Right) conditions at 5- and 8-month ages. (J) Small intestines of male 5xFAD mice. Data are pooled from 5 independent experiments (n = 4 to 14 per group); two-way ANOVA. Cells were stimulated using PMA and ionomycin in the presence of Brefeldin A prior to intracellular cytokine staining. Error bars indicate SEM. *P < 0.05, **P < 0.01, ***P < 0.001, ****P < 0.0001.

The most significant risk factor for AD is aging (2023 Alzheimer’s disease facts and figures). To determine how immune responses progressively change in mouse models, we examined mice at multiple ages. We found that systemic immune responses increased with age in 3xTg mice, especially in SPF mice, with increased T and B cell numbers in the spleen. However, local immune responses in the lymph nodes remained the same or declined with age. The CD4^+^ T cell counts that we observed at 7 months of age in SPF female 3xTg animals remained elevated in the spleens and MLNs but declined by half in the CLNs from 7 to 15 months of age (Fig. 5A–D). In contrast to SPF female mice, CD4^+^ T cell numbers remained the same or declined in GF female mice (Fig. S12A–D). Both GF and SPF male mice showed progressive increases in CD4^+^ T cells in the spleen, with minimal change in other tissues as animals aged (Fig. S12E–L). These data indicate that changes in systemic spleen CD4^+^ T cell responses with age are influenced by microbiome status to a greater extent in female than male mice. B cell numbers mirrored CD4^+^ T cell numbers in female (Fig. 5E–H, S13A–D) and male (Fig. S13E–L) 3xTg mice.

In SPF 5xFAD mice, IL-17A^+^ T cells were attenuated in the small intestine from 5 to 8 months in both female and male mice (Fig. 5I and J). However, B cell numbers remained elevated in the spleens of SPF female, but not SPF male, mice from 5 to 8 months of age, indicating sustained systemic B cells in SPF but not GF mice (Fig. S14A and B). In the MLNs and CLNs, B cell numbers largely remained the same under SPF and GF conditions, with some variability (Fig. S14D, F, and H), and GF male mice showed a decline in B cell counts with age in the MLNs (Fig. S14D). We found an age-dependent increase in B cells that was independent of disease in the superficial and deep CLNs and MLNs of SPF female mice (Fig. S14C, E, and G). The differences in immune responses between 3xTg and 5xFAD mice as animals age, especially in GF conditions, suggest differing mechanisms of immune activation and resolution. Overall, these data reveal that increased T and B cell numbers and CD4^+^ T cell cytokine production develop early in some models of AD and can be durable or can resolve as animals age depending on variables such as sex and microbiome status.

### 3.5. Both 3xTg and 5xFAD mice show altered learning and cognition in the absence of a microbiome

**Fig. 6.**
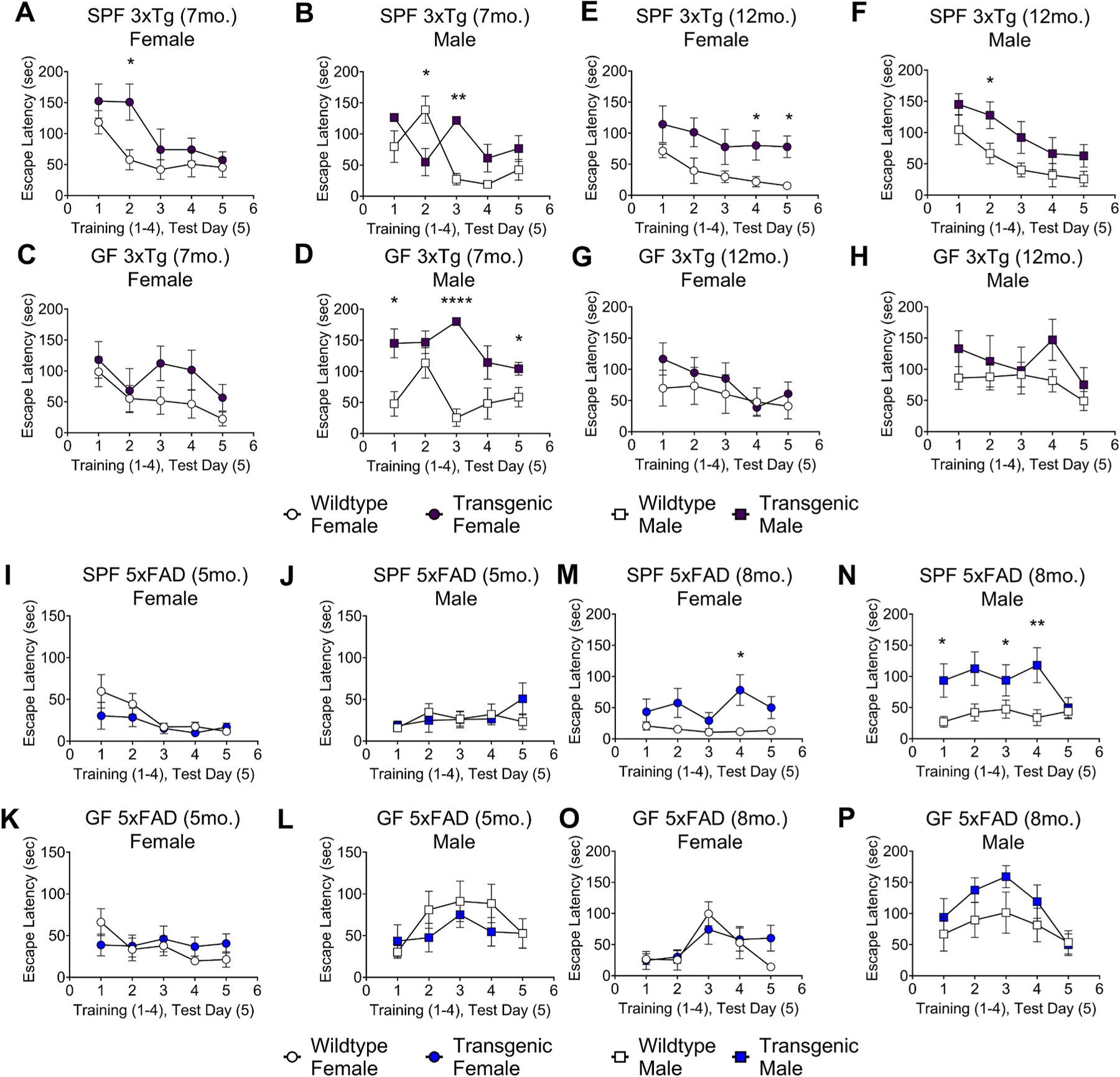
Both 3xTg and 5xFAD mice show modestly altered learning and cognition. (A) Training (1–4) and test (5) day mean escape latency in Barnes Maze for 7-month-old female 3xTg mice compared to wildtype controls in specific pathogen-free (SPF) conditions. (B) Male mice in SPF conditions. (C) Female mice in germ-free (GF) conditions. (D) Male mice in GF conditions. Data are pooled from 2 independent experiments (n = 6 to 11 per group); Mann-Whitney *U* test comparing transgenic to wildtype mice. (E) 12-month-old female 3xTg mice in SPF conditions. (F) Male mice in SPF conditions. (G) Female mice in GF conditions. (H) Male mice in GF conditions. Data are pooled from 2 independent experiments (n = 5 to 11 per group). (I) 5-month-old female 5xFAD mice in SPF conditions. (J) Male mice in SPF conditions. (K) Female mice in GF conditions. (L) Male mice in GF conditions. Data are pooled from 2 independent experiments (n = 8 to 14 per group); Mann-Whitney *U* test comparing transgenic to wildtype mice. (M) 8-month-old female 5xFAD mice in SPF conditions. (N) Male mice in SPF conditions. (O) Female mice in GF conditions. (P) Male mice in GF conditions. Data are pooled from 2 independent experiments (n = 5 to 13 per group); Mann-Whitney *U* test comparing transgenic to wildtype mice. Error bars indicate SEM. *P < 0.05, **P < 0.01, ****P < 0.0001.

We next sought to determine whether differences in immune response correlate with cognitive outcomes. It has been previously reported that AD mouse models display reduced amyloid plaques and improved cognitive function under GF conditions or after antibiotic treatment, compared to mice with an intact microbiome (Minter et al., 2017; Minter et al., 2016; Harach et al., 2017). However, reported behavioral outcomes in these two preclinical models have varied widely with regard to presence of a phenotype, age, behavioral paradigm tested, and examination of the influence of the gut microbiota (Dennison et al., 2021; Creighton et al., 2019). To obtain a broad view of cognitive performance in both models across aging, SPF and GF mice were evaluated via the modified Barnes maze, Y maze, and novel object recognition tests. Overall, phenotypes were subtle, consistent with several previous studies (Dennison et al., 2021; Creighton et al., 2019; Stover et al., 2015). However, we observed the most robust differences in cognitive function between transgenic and wildtype mice in the modified Barnes maze, with differences in both training learning and test performance (Figs. 6 and S15).

Cognitive deficits, as measured by increased latencies to escape the maze, were more apparent in SPF mice comparing wildtype to AD models. Under GF conditions, both wildtype and transgenic mice showed altered performance, suggesting that the microbiome can influence behavioral outcomes independent of genotype. We observed cognitive deficits in both sexes in SPF 3xTg mice at 7 and 12 months of age (Fig. 6A, B, E, and F) and SPF 5xFAD mice at 8 months of age (Fig. 6M and N). Female mice showed slightly reduced or unchanged performance under GF conditions (Fig. 6C, G, K, and O), whereas 7-month-old GF 3xTg males showed significantly decreased cognitive performance (Fig. 6D). In addition to comparing wildtype vs. transgenic mice, we also assessed Barnes maze performance between GF and SPF conditions (Fig. S15A–P). As already noted, 7-month-old GF 3xTg males showed poorer performance than their SPF counterparts (Fig. S15D). In the 5xFAD experiment, wildtype males showed poorer performance in GF conditions, but the differences were only statistically significant at 5 months of age (Fig. S15J and N). Female wildtype mice that were controls for the 5xFAD group also showed slightly worse cognitive behavior under GF vs. SPF conditions at 8 months of age (Fig. S15M). Overall, the genotype effect appears stronger than the microbiome effect, particularly in 3xTg mice, though the interesting worsening of outcomes in GF compared to SPF mice in multiple groups may be a function of unknown developmental or metabolic effects that require further investigation.

Spatial memory testing with the Y maze did not show significant cognitive decline in male or female 3xTg mice at 7 or 12 months of age in either GF or SPF conditions, with the exception of 7-month-old GF 3xTg males (Fig. S16A–C). Seven and 12-month-old SPF female 3xTg mice performed better than wildtype mice (Fig. S16A and B), consistent with a previous report (Stover et al., 2015). 5xFAD mice showed no deficits compared to wildtype mice in Y maze at 5 or 8 months of age (Fig. S16D and E). We did not observe any deficits in novel object recognition in male or female 3xTg or 5xFAD mice (Fig. S17), except for SPF 3xTg females at 7 months of age (Fig. S17A) and GF 5xFAD males at 8 months of age (Fig. S17E). Together, these data highlight deficits in spatial and working memory in the 3xTg and 5xFAD mouse models, with negligible differences in recognition memory. Our results support the notion that age, immune state, sex, and the microbiome impact outcomes of cognitive performance, with the most significant effects by microbiome being on the interaction between sex and immune responses.

## 4. Discussion

The microbiome is an important contributor to immunity, and it has recently been implicated in neurodegenerative disorders such as AD (Morais et al., 2021; De Luca and Shoenfeld, 2018; Fan and Pedersen, 2021). Whether and how gut bacteria actively contribute to AD pathophysiology remains an open question. In this study, we examined immune and cognitive profiles in two transgenic mouse models of AD in the presence or absence of gut bacteria to understand how the microbiome shapes immune responses associated with AD. We found that the microbiome marginally influences immune cell proportions in the CNS-draining lymph nodes in both mouse models, but strongly modulates systemic immune responses, the cytokine response in local lymphoid tissues, and cognitive deficits. Notably, the immune state in these mice was modulated by sex, and this sex effect differed between local and systemic lymphoid tissues and between mice with an intact and absent microbiome.

In the 3xTg and 5xFAD mouse models, we found two contrasting immune environments: (1) in 3xTg mice, increased T and B cell numbers and cytokine production were evident throughout secondary lymphoid tissues, including those associated with the intestine and CNS lymphatics, as well as systemically; (2) in 5xFAD mice, changes in immune cell numbers and cytokine production were more limited and, while somewhat systemic, largely localized to intestinal tissue and CNS-draining lymph nodes. In both models, responses were driven by activated adaptive immunity, with elevated CD4^+^ T cells and B cells. However, as evident from the grossly elevated spleen cell counts in 3xTg mice compared to the more mildly elevated spleen cell counts of 5xFAD mice, 3xTg mice had a more pronounced immune response. The most significant genetic difference between the models is expression of tau protein, in addition to amyloid precursor protein and presenilin, in 3xTg mice, which results in tau aggregation (tauopathy) as well as Aβ deposition (Javonillo et al., 2022). Ectopic expression of human tau in mice has been shown to increase adaptive immune responses and brain atrophy in another AD model and may be a factor in the widespread immune responses we observed in 3xTg mice (Chen et al., 2023; Gate et al., 2020; Wang et al., 2024; Laurent et al., 2017).

Genotype and microbiome interactions are complex and require careful experimental setup and controls. One complication is the coprophagic activity of co-housed mice. In this study, mice from both models were housed by genotype (i.e., wildtype or mutant) after weaning, preventing microbiome exchange between genotypes in adulthood. However, in SPF 5xFAD mice, microbial exchange could occur between wildtype and mutant mice before weaning due to the hemizygous breeding strategy. This was not the case for the SPF 3xTg mice, where wildtype and mutant mice were derived from homozygous breeding. Of note, we observed differences in IL-17A production between the SPF wildtype controls of each model. This may be due to the differences in breeding strategy outlined above that could lead to divergence of the microbiomes, which can influence IL-17A production. Finally, because wildtype and mutant embryos were derived separately into germ-free colonies, there was no co-housing or coprophagic exchange between genotypes prior to germ-free rearing, minimizing early microbial influences across genotypes.

Previous studies have shown that adaptive immunity participates in both protective and pathogenic processes in AD mouse models (Marsh et al., 2016; Kim et al., 2021; Gate et al., 2020; Wang et al., 2024; Laurent et al., 2017; Marchese et al., 2014; Chen and Holtzman, 2022; Dinkins et al., 2015). Complete abrogation of T and B cell responses in the 5xFAD model by genetic knockout of *Rag2 and Il2rg* results in increased amyloid aggregation and decreased brain expression of genes related to immunoglobulin antibody production and associated signaling, indicating a protective role for the adaptive immune system (Marsh et al., 2016). Paradoxically, the loss of B cells alone results in reduced amyloid aggregation and improved cognitive function in 3xTg mice (Kim et al., 2021). Studies indicate that B cell-produced autoantibodies are present in both 3xTg and 5xFAD mouse models (Marchese et al., 2014; Dinkins et al., 2015). Chronic inflammatory states may contribute to pathogenic mechanisms that promote cellular stress, death, and the progression of AD. T and B cells, among other immune cells, have been shown to promote chronic inflammation through antibody production, activation of additional immune cells, and inflammatory cytokine production (Kim et al., 2021; Chen and Holtzman, 2022; Sabatino et al., 2019; Häusser-Kinzel and Weber, 2019). We observed that adaptive immune responses remained elevated with age in 3xTg mice, in contrast to the attenuation of immune responses in 5xFAD mice. This indicates a chronic elevation of immune responses in 3xTg mice, which may contribute to more severe pathological outcomes.

Here, we find that immune cell proportions in the brain-draining lymph nodes in both the 3xTg and 5xFAD models are influenced by sex, with female mice showing earlier activation of adaptive immunity. This corresponds with a previously reported early rise in Aβ in the brains of female 3xTg and 5xFAD mice (Javonillo et al., 2022; Forner et al., 2021). The cause of this accelerated pathology in female mice is not well understood. Previous studies suggested that pathology in the 3xTg and 5xFAD models is influenced by sex hormones, as alterations of sex hormones during early development and adulthood in 3xTg mice, or modulation of estrogen receptor activity in 5xFAD mice, can modify Aβ accumulation (Carroll et al., 2010; Farkas et al., 2022; Quinn et al., 2022; Carroll et al., 2007). Notably, an early study in humans identified that estrogen-based hormone therapy reduces the risk of AD in women (Paganini-Hill and Henderson, 1994).

We observed elevated T and B cell counts and cytokine production in the draining lymph nodes of both the brain and intestine, indicating that immune responses may be activated and/or maintained by both brain and peripheral amyloid or tau pathology. There is significantly more (25x) Aβ peptide in the circulation of 3xTg compared to 5xFAD mice, which might contribute to the increased immune cell numbers in the spleen and liver of 3xTg mice observed in this study and others (Marchese et al., 2014; Yang et al., 2015; Cho et al., 2016; Botella Lucena et al., 2022). The liver, kidneys, spleen, and small intestine are important sites for the clearance of circulating Aβ and may act as hubs for activation of systemic immunity (Ghiso et al., 2004; Yu et al., 2022). Previous work demonstrated reduced amyloid concentration in the brains of GF or antibiotic-treated mice (Minter et al., 2017; Minter et al., 2016; Harach et al., 2017); therefore, our expectation was to find reduced systemic inflammation. Remarkably, while 3xTg female mice showed attenuated systemic immune responses under GF conditions, 3xTg male mice had elevated T and B cell numbers. A previous study noted that male 3xTg mice develop a more robust autoimmune-like response, with serum antibodies against cell nuclei (Kapadia et al., 2018). In 5xFAD mice, we observed reduced T and B cell frequencies in GF compared to SPF conditions. These findings suggest a sex- and microbiome-mediated effect on systemic immune responses which requires further study.

Increased immune cell numbers and CD4^+^ T cell cytokine production corresponded with cognitive deficits in 3xTg and 5xFAD mice, an effect most pronounced in 3xTg female mice at early ages and in SPF conditions. Of note, GF 3xTg male mice had significant cognitive deficits, which corresponded with an increased systemic inflammatory response, suggesting that severe inflammatory responses may be associated with impaired cognitive function, although the mechanism is not clear. Previous studies showed reduced Aβ and improved cognition in GF or antibiotic-treated mice; however, the AD models used in those studies did not overexpress human tau (Minter et al., 2017; Minter et al., 2016; Harach et al., 2017). Modulating tau or immunomodulatory interventions in GF 3xTg male mice might help to disentangle the interplay between the genotype, microbiome, immune responses, and cognition.

Limitations of this work include the scope of the immune response survey, restricted mainly to secondary lymphoid tissues and the adaptive immune response, and variability in the results of cognitive testing. Innate populations were excluded from the current study to focus on adaptive immune differences across genotype, sex, and microbial status. Future studies should include innate immune profiling both in the brain and periphery (e.g., microglia, monocytes, dendritic cells). Although sex differences were noted in the immune responses, sex hormone levels were not examined, which would be beneficial in follow on studies. Discrepancies in reported cognitive deficits exist across studies (Carroll et al., 2007; Dennison et al., 2021), perhaps due to technical differences between testing conditions, different microbiomes between animal facilities, and other variables. Differences in anxiety levels should be examined through additional testing (e.g., open field). In future studies, metagenomic sequencing of the microbiome may help determine causes of variability (Zuffa et al., 2025). For immune response evaluation, we chose secondary lymphoid tissues because they represent hubs of immune activation. Local tissue responses may provide additional insight into the mechanisms of immune activation and resolution. Amyloid beta (Aβ) and tau analyses were not performed in this study but could be included in future to assess brain pathology differences between groups, which is a feature of these animal models.

Germ-free conditions revealed both attenuated and exacerbated immune alterations depending on the AD model and sex, which may have translational implications. The association of tau overexpression with elevated systemic immune responses in GF 3xTg male mice and poor cognitive performance highlights the relevance of microbiota-immune crosstalk in conjunction with tau pathology and disease outcomes. Further investigation of the potential connection of tau pathology, immune responses, and the microbiome in clinical studies is warranted. Given the observation that estrogen-based hormone therapy reduces the risk of AD in women (Paganini-Hill and Henderson, 1994), these studies should also evaluate sex and hormone levels as covariates.

In conclusion, we found that the microbiome influences both immune and cognitive outcomes in a sex-dependent manner in two mouse models of AD. Increased adaptive immune responses were observed in both female and male mice and were attenuated in germ-free conditions. Unexpectedly, GF male 3xTg mice maintained elevated adaptive immune and cytokine responses that correlated with cognitive deficits, indicating a protective role for the microbiome in male mice in this model. These data demonstrate a sex and microbiome interaction in the immune response and cognitive performance of AD mouse models. Future studies may focus on microbiome-influenced mechanisms that intersect with sex hormone signaling and immune responses to determine their effects on AD-like outcomes in mice, and perhaps in humans.

## Supplementary Material

MMC2

Supp.figures

Appendix A. Supplementary data

Supplementary data to this article can be found online at https://doi.org/10.1016/j.bbi.2025.07.028.

## Data Availability

Data will be made available on request.
