# Supplementary material for: Genotype and microbiome shape immunity in a sex-specific manner in mouse models of Alzheimer’s disease
Source: Brain Behav Immun. Author manuscript; Available in PMC 2025 Oct 2. (PMC12490345; doi:10.1016/j.bbi.2025.07.028)

Genotype and microbiome shape immunity in a sex-specific manner in mouse models of Alzheimer's disease

John W. Bostick, T. Jaymie Connerly, Taren Thron, Brittany D. Needham, Matheus de Castro Fonseca, Rima Kaddurah-Daouk, Rob Knight, and Sarkis K. Mazmanian

Table 1.1 – $CD4^+$ T cells (% of $CD45^+$) [5xFAD 5 Months]

Table 1.2 – $CD4^+$ T cell Counts [5xFAD 5 Months]

Table 1.3 - T cell Counts [5xFAD 5 Months]

Table 1.4 - B cell Counts [5xFAD 5 Months]

Table 1.5 – $Foxp3^+$ T cells (% $CD4^+$ T cells) [5xFAD 5 Months]

Table 1.6 – $IFN\gamma^+$ T cells (% $CD4^+$ T cells) [5xFAD 5 Months]

Table 1.7 – IFNγ MFI [5xFAD 5 Months]

Table 1.8 – IL-$17A^+$ T cells (% $CD4^+$ T cells) [5xFAD 5 Months]

Table 1.9 – IL-17A MFI [5xFAD 5 Months]

Table 1.10 – IL-4^+ T cells (% $CD4^+$ T cells) [5xFAD 5 Months]

Table 1.11 – IL-4 MFI [5xFAD 5 Months]

Table 1.12 – GM-CSF^+ T cells (% $CD4^+$ T cells) [5xFAD 5 Months]

Table 1.13 – GM-CSF MFI [5xFAD 5 Months]

Table 2.1 – $CD4^+$ T cells (% of $CD45^+$) [3xTg 7 Months]

Table 2.2 – $CD4^+$ T cell Counts [3xTg 7 Months]

Table 2.3 – T cell Counts [3xTg 7 Months]

Table 2.4 – B cell Counts [3xTg 7 Months]

Table 2.5 – $Foxp3^+$ T cells (% $CD4^+$ T cells) [3xTg 12 Months]

Table 2.6 – $IFN\gamma^+$ T cells (% $CD4^+$ T cells) [3xTg 12 Months]

Table 2.7 – IFNγ MFI [3xTg 12 Months]

Table 2.8 – IL-$17A^+$ T cells (% $CD4^+$ T cells) [3xTg 12 Months]

Table 2.9 – IL-17A MFI [3xTg 12 Months]

Table 1.1 – CD4$^+$ T cells (% of CD45$^+$) [5xFAD 5 Months]

Group Comparison	MLN	SP	SC	DC
**Females:**				
GF:WT vs. SPF:WT	— (0.9404)	— (0.9608)	— (0.6799)	— (0.9231)
GF:5xFAD vs. SPF:5xFAD	↓ (0.0062)	— (0.1466)	— (0.4925)	↑ (0.5862)
GF:5xFAD vs. GF:WT	— (0.4065)	— (0.9989)	— (0.9489)	— (0.9214)
SPF:5xFAD vs. SPF:WT	↑ (0.1078)	— (0.4974)	— (0.9812)	— (>0.9999)
GF:WT vs. SPF:5xFAD	— (0.2591)	— (0.2229)	— (0.2232)	— (0.8932)
GF:5xFAD vs. SPF:WT	— (0.8145)	— (0.9138)	— (0.8934)	↑ (0.6877)
**Males:**				
GF:WT vs. SPF:WT	↓↓ (<0.0001)	— (0.9296)	— (0.0856)	— (0.7443)
GF:5xFAD vs. SPF:5xFAD	↓ (0.0341)	— (0.6642)	— (0.9867)	↓ (0.1283)
GF:5xFAD vs. GF:WT	↑↑ (0.0227)	↓ (0.2217)	↑ (0.0932)	— (0.9814)
SPF:5xFAD vs. SPF:WT	— (0.8502)	— (0.9794)	— (0.9947)	— (0.8392)
GF:WT vs. SPF:5xFAD	↓↓ (0.0005)	— (0.847)	— (0.2496)	↓ (0.4696)
GF:5xFAD vs. SPF:WT	↓ (0.0155)	— (0.2219)	— (0.999)	— (0.1894)

Change from mean: — No change; ↑ (≥20%); ↑↑ (≥50%); ↑↑↑ (≥100%); ↓ (≤20%); ↓↓ (≤50%); ↓↓↓ (≤100%); no data (n.d.)
ANOVA: (adjusted p-value)
GF Germ-free; DC Deep cervical lymph nodes; LI Large Intestine; MLN Mesenteric lymph nodes; SC Superficial cervical lymph nodes; SI Small intestine; SP Spleen; SPF Specific pathogen-free; WT wild-type

Table 1.2 – $CD4^+$ T cell Counts [5xFAD 5 Months]

Group Comparison	MLN	SP	SC	DC
**Females:**				
GF:WT vs. SPF:WT	— (0.9903)	↑↑ (0.0003)	— (0.5824)	↑↑↑ (0.0064)
GF:5xFAD vs. SPF:5xFAD	— (0.7553)	↓↓ (<0.0001)	↑ (0.0141)	↑↑↑ (0.0043)
GF:5xFAD vs. GF:WT	— (0.9803)	— (0.0805)	— (0.3395)	↓ (0.0362)
SPF:5xFAD vs. SPF:WT	↑ (0.3992)	↑↑↑ (<0.0001)	↓ (0.0578)	↓↓ (0.1367)
GF:WT vs. SPF:5xFAD	↓ (0.5542)	↓ (<0.0001)	— (0.5005)	↑↑↑ (<0.0001)
GF:5xFAD vs. SPF:WT	— (0.9021)	↑ (0.0848)	— (0.9941)	↑ (0.6732)
**Males:**				
GF:WT vs. SPF:WT	↓↓ (0.0005)	— (0.7032)	↓ (0.5113)	↑ (0.9641)
GF:5xFAD vs. SPF:5xFAD	— (0.637)	↓ (0.0002)	↓ (0.4631)	↓↓ (0.0185)
GF:5xFAD vs. GF:WT	↑↑↑ (<0.0001)	↓ (0.2291)	— (0.9459)	↓↓ (0.4672)
SPF:5xFAD vs. SPF:WT	↑ (0.1639)	— (0.3175)	— (0.8976)	↑↑ (0.1596)
GF:WT vs. SPF:5xFAD	↓↓ (0.0001)	↓ (0.1285)	↓ (0.3317)	↓ (0.6166)
GF:5xFAD vs. SPF:WT	— (0.3886)	↓ (0.0014)	— (0.7034)	↓↓ (0.4151)

Change from mean: — No change; ↑ (≥20%); ↑↑ (≥50%); ↑↑↑ (≥100%); ↓ (≤20%); ↓↓ (≤50%); ↓↓↓ (≤100%); no data (n.d.)
ANOVA: (adjusted p-value)
GF Germ-free; DC Deep cervical lymph nodes; LI Large Intestine; MLN Mesenteric lymph nodes; SC Superficial cervical lymph nodes; SI Small intestine; SP Spleen; SPF Specific pathogen-free; WT wild-type

Table 1.3 - T cell Counts [5xFAD 5 Months]

Group Comparison	MLN	SP	SC	DC	LI	SI
**Females:**						
GF:WT vs. SPF:WT	— (0.4283)	↑↑ (<0.0001)	— (0.3908)	↑↑↑ (0.0001)	↑↑↑ (<0.0001)	— (0.7401)
GF:5xFAD vs. SPF:5xFAD	↑↑ (<0.0001)	↓↓ (<0.0001)	↑ (0.0382)	↑↑↑ (0.0007)	↑↑ (0.0722)	— (0.9729)
GF:5xFAD vs. GF:WT	↑ (0.0547)	↓ (0.0034)	— (0.5575)	↓ (0.0008)	↓ (0.0038)	↓ (0.1968)
SPF:5xFAD vs. SPF:WT	↓ (0.0038)	↑↑↑ (<0.0001)	↓ (0.0279)	↓↓ (0.0589)	— (0.963)	— (0.9767)
GF:WT vs. SPF:5xFAD	↑ (0.1191)	↓ (0.0001)	— (0.5113)	↑↑↑ (<0.0001)	↑↑↑ (<0.0001)	↑ (0.4448)
GF:5xFAD vs. SPF:WT	— (0.8158)	↑ (0.3144)	— (0.9713)	↑ (0.5527)	↑↑ (0.0309)	— (0.8372)
**Males:**						
GF:WT vs. SPF:WT	↓↓ (0.0003)	— (0.5155)	↓ (0.5756)	↑ (0.9209)	↑↑↑ (<0.0001)	— (0.9569)
GF:5xFAD vs. SPF:5xFAD	— (0.9677)	↓↓ (<0.0001)	↓ (0.5882)	↓↓ (0.0037)	— (0.9931)	↓ (0.8316)
GF:5xFAD vs. GF:WT	↑↑↑ (<0.0001)	↓ (0.2122)	— (0.9555)	↓↓ (0.2844)	↓ (0.0163)	↓↓ (0.1913)
SPF:5xFAD vs. SPF:WT	— (0.8467)	↑ (0.1643)	— (0.9468)	↑↑ (0.0645)	↑ (0.4059)	↓ (0.6716)
GF:WT vs. SPF:5xFAD	↓↓ (0.0012)	↓ (0.0392)	↓ (0.4418)	↓ (0.4874)	↑↑ (0.0105)	↑↑ (0.4972)
GF:5xFAD vs. SPF:WT	— (0.9339)	↓ (0.0004)	— (0.7541)	↓↓ (0.263)	↑↑ (0.2284)	↓↓ (0.2666)

Change from mean: — No change; ↑ (≥20%); ↑↑ (≥50%); ↑↑↑ (≥100%); ↓ (≤20%); ↓↓ (≤50%); ↓↓↓ (≤100%); no data (n.d.)
ANOVA: (adjusted p-value)
GF Germ-free; DC Deep cervical lymph nodes; LI Large Intestine; MLN Mesenteric lymph nodes; SC Superficial cervical lymph nodes; SI Small intestine; SP Spleen; SPF Specific pathogen-free; WT wild-type

Table 1.4 - B cell Counts [5xFAD 5 Months]

Group Comparison	MLN	SP	SC	DC	LI	SI
**Females:**						
GF:WT vs. SPF:WT	— (0.8824)	↑↑↑ (<0.0001)	— (0.5784)	↑↑ (0.0855)	↑↑↑ (0.0004)	↑↑ (0.017)
GF:5xFAD vs. SPF:5xFAD	↑↑↑ (<0.0001)	↓↓ (<0.0001)	— (0.9493)	↑↑↑ (0.0982)	↑↑ (0.3007)	↑↑ (0.0538)
GF:5xFAD vs. GF:WT	↑↑ (0.0028)	— (0.1075)	— (>0.9999)	↓ (0.0338)	↓ (0.0685)	↓ (0.1133)
SPF:5xFAD vs. SPF:WT	↓↓ (0.0266)	↑↑↑ (<0.0001)	— (0.8774)	↓↓ (0.147)	↑ (0.9433)	↓ (0.5534)
GF:WT vs. SPF:5xFAD	↑↑↑ (0.0023)	↓ (<0.0001)	— (0.9417)	↑↑↑ (0.0002)	↑↑↑ (0.0011)	↑↑↑ (0.0002)
GF:5xFAD vs. SPF:WT	↑↑ (0.0005)	↑↑ (0.0018)	— (0.5726)	— (0.9998)	↑↑ (0.1242)	↑ (0.6898)
**Males:**						
GF:WT vs. SPF:WT	↑↑↑ (0.0661)	— (0.499)	— (0.9721)	↑↑ (0.0484)	↑↑ (0.0013)	↑ (0.681)
GF:5xFAD vs. SPF:5xFAD	↑↑↑ (0.0186)	↓ (0.1822)	↓ (0.4273)	↓ (0.2132)	— (0.8247)	— (0.9967)
GF:5xFAD vs. GF:WT	↑ (0.1639)	— (0.9146)	↓ (0.488)	↓↓ (0.015)	↓ (0.2086)	↓↓ (0.3128)
SPF:5xFAD vs. SPF:WT	↑ (0.9548)	— (0.4856)	— (0.966)	↑ (0.525)	↑↑ (0.0367)	↓ (0.7599)
GF:WT vs. SPF:5xFAD	↑↑ (0.4763)	↓ (0.1182)	— (>0.9999)	↑ (0.5782)	— (0.6903)	↑↑↑ (0.285)
GF:5xFAD vs. SPF:WT	↑↑↑ (<0.0001)	— (0.769)	— (0.483)	↓ (0.7716)	↑ (0.2291)	↓ (0.7318)

Change from mean: — No change; ↑ (≥20%); ↑↑ (≥50%); ↑↑↑ (≥100%); ↓ (≤20%); ↓↓ (≤50%); ↓↓↓ (≤100%); no data (n.d.)
ANOVA: (adjusted p-value)
GF Germ-free; DC Deep cervical lymph nodes; LI Large Intestine; MLN Mesenteric lymph nodes; SC Superficial cervical lymph nodes; SI Small intestine; SP Spleen; SPF Specific pathogen-free; WT wild-type

Table 1.5 – Foxp3$^+$ T cells (% CD4$^+$ T cells) [5xFAD 5 Months]

Group Comparison	MLN	SP	SC	DC	LI	SI
**Females:**						
GF:WT vs. SPF:WT	↑ (0.366)	↑ (0.2484)	↑ (0.5031)	↑ (0.9299)	— (0.989)	↑ (0.005)
GF:5xFAD vs. SPF:5xFAD	↓ (0.2921)	↓ (0.0409)	— (0.7699)	↓ (0.6806)	— (0.6319)	— (0.9601)
GF:5xFAD vs. GF:WT	— (0.9906)	— (0.9996)	— (0.6662)	↑ (0.8526)	— (0.7572)	— (0.4558)
SPF:5xFAD vs. SPF:WT	↑↑ (0.0194)	↑↑ (0.0018)	↑ (0.5962)	↑↑↑ (0.2707)	— (>0.9999)	— (0.2403)
GF:WT vs. SPF:5xFAD	↓ (0.2112)	↓ (0.0671)	— (0.9969)	↓ (0.3189)	— (0.9917)	— (0.3101)
GF:5xFAD vs. SPF:WT	↑↑ (0.2439)	↑ (0.2524)	— (0.9729)	↑↑ (0.6544)	— (0.6659)	↑ (0.0671)
**Males:**						
GF:WT vs. SPF:WT	↑↑↑ (<0.0001)	↑ (0.0437)	↑↑ (0.0005)	↑↑ (0.0077)	— (0.2553)	— (0.9804)
GF:5xFAD vs. SPF:5xFAD	↑↑↑ (<0.0001)	↓ (0.2998)	↑ (0.0186)	↑↑↑ (0.0024)	↑ (0.0085)	↓ (0.285)
GF:5xFAD vs. GF:WT	↓ (0.004)	↓↓ (0.0004)	— (0.4854)	— (0.9707)	— (0.7851)	— (0.4697)
SPF:5xFAD vs. SPF:WT	— (0.9924)	— (0.9935)	— (0.9934)	— (0.9114)	— (0.8247)	— (0.765)
GF:WT vs. SPF:5xFAD	↑↑↑ (<0.0001)	↑↑ (0.0565)	↑↑ (0.001)	↑↑↑ (0.0037)	↑ (0.0955)	— (0.9335)
GF:5xFAD vs. SPF:WT	↑↑↑ (<0.0001)	↓ (0.1115)	↑ (0.0112)	↑↑ (0.0049)	↑ (0.0223)	— (0.5733)

Change from mean: — No change; ↑ (≥20%); ↑↑ (≥50%); ↑↑↑ (≥100%); ↓ (≤20%); ↓↓ (≤50%); ↓↓↓ (≤100%); no data (n.d.)
ANOVA: (adjusted p-value)
GF Germ-free; DC Deep cervical lymph nodes; LI Large Intestine; MLN Mesenteric lymph nodes; SC Superficial cervical lymph nodes; SI Small intestine; SP Spleen; SPF Specific pathogen-free; WT wild-type

Table 1.6 – IFNγ⁺ T cells (% CD4⁺ T cells) [5xFAD 5 Months]

Group Comparison	MLN	SP	SC	LI	SI
**Females:**					
GF:WT vs. SPF:WT	— (0.9146)	↓ (0.3977)	— (0.9932)	— (0.8605)	↑↑ (0.238)
GF:5xFAD vs. SPF:5xFAD	— (>0.9999)	↓ (0.0051)	↓ (0.0279)	— (0.9289)	↑↑↑ (0.002)
GF:5xFAD vs. GF:WT	— (0.7728)	↑ (0.819)	— (0.9886)	— (0.6781)	↑ (0.3565)
SPF:5xFAD vs. SPF:WT	— (>0.9999)	↑↑ (0.1678)	↑↑ (0.0838)	— (0.9746)	— (0.9977)
GF:WT vs. SPF:5xFAD	— (0.8784)	↓↓ (0.001)	↓ (0.0688)	— (0.9897)	↑↑ (0.1142)
GF:5xFAD vs. SPF:WT	— (0.9994)	↓ (0.7877)	— (>0.9999)	— (0.9995)	↑↑↑ (0.0094)
**Males:**					
GF:WT vs. SPF:WT	↑ (0.6675)	↓↓ (0.0638)	↑ (0.3028)	↓↓ (0.0254)	↓↓ (0.0399)
GF:5xFAD vs. SPF:5xFAD	— (0.981)	↓↓ (0.2221)	— (0.7719)	↑↑↑ (0.1752)	↑↑↑ (0.1959)
GF:5xFAD vs. GF:WT	↓ (0.2327)	— (0.9993)	— (0.6661)	↑↑↑ (0.0916)	↑↑↑ (0.1979)
SPF:5xFAD vs. SPF:WT	— (0.9282)	— (0.9927)	↑ (0.3656)	↓↓ (0.0596)	↓↓ (0.045)
GF:WT vs. SPF:5xFAD	↑↑ (0.4208)	↓↓ (0.1982)	— (0.9944)	— (0.9887)	— (>0.9999)
GF:5xFAD vs. SPF:WT	↓ (0.7172)	↓↓ (0.0701)	— (0.93)	— (0.9899)	— (0.9972)

Change from mean: — No change; ↑ (≥20%); ↑↑ (≥50%); ↑↑↑ (≥100%); ↓ (≤20%); ↓↓ (≤50%); ↓↓↓ (≤100%); no data (n.d.)
ANOVA: (adjusted p-value)
GF Germ-free; DC Deep cervical lymph nodes; LI Large Intestine; MLN Mesenteric lymph nodes; SC Superficial cervical lymph nodes; SI Small intestine; SP Spleen; SPF Specific pathogen-free; WT wild-type

Table 1.7 – IFNγ MFI [5xFAD 5 Months]

Group Comparison	MLN	SP	SC	LI	SI
**Females:**					
GF:WT vs. SPF:WT	— (0.3474)	— (>0.9999)	— (0.1849)	— (0.0269)	— (0.9903)
GF:5xFAD vs. SPF:5xFAD	↓↓ (<0.0001)	— (0.5227)	↓↓ (<0.0001)	— (0.9991)	— (0.1905)
GF:5xFAD vs. GF:WT	— (0.9632)	— (0.8478)	— (0.9663)	— (0.9964)	— (0.995)
SPF:5xFAD vs. SPF:WT	↑↑↑ (<0.0001)	— (0.9532)	↑↑ (<0.0001)	— (0.0669)	— (0.6204)
GF:WT vs. SPF:5xFAD	↓↓ (<0.0001)	— (0.9293)	↓↓ (<0.0001)	— (>0.9999)	— (0.3172)
GF:5xFAD vs. SPF:WT	— (0.1589)	— (0.9072)	— (0.0728)	— (0.031)	— (0.9555)
**Males:**					
GF:WT vs. SPF:WT	— (0.4112)	↓ (0.1291)	— (0.198)	↓ (0.144)	— (0.2896)
GF:5xFAD vs. SPF:5xFAD	↓ (0.151)	↓ (0.3227)	— (0.9979)	↓ (0.25)	— (0.9851)
GF:5xFAD vs. GF:WT	— (0.5985)	— (0.9974)	— (0.6962)	— (0.999)	— (0.9225)
SPF:5xFAD vs. SPF:WT	— (0.8372)	— (0.9995)	— (0.6792)	— (0.9841)	— (0.6212)
GF:WT vs. SPF:5xFAD	— (0.8815)	↓ (0.2584)	— (0.8024)	↓ (0.3697)	— (0.9907)
GF:5xFAD vs. SPF:WT	↓ (0.0119)	↓ (0.168)	— (0.7744)	↓ (0.0693)	— (0.9321)

Change from mean: — No change; ↑ (≥20%); ↑↑ (≥50%); ↑↑↑ (≥100%); ↓ (≤20%); ↓↓ (≤50%); ↓↓↓ (≤100%); no data (n.d.)
ANOVA: (adjusted p-value)
GF Germ-free; DC Deep cervical lymph nodes; LI Large Intestine; MLN Mesenteric lymph nodes; SC Superficial cervical lymph nodes; SI Small intestine; SP Spleen; SPF Specific pathogen-free; WT wild-type

Table 1.8 – IL-17A$^+$ T cells (% CD4$^+$ T cells) [5xFAD 5 Months]

Group Comparison	MLN	SP	SC	LI	SI
**Females:**					
GF:WT vs. SPF:WT	↓ (0.62)	↓ (0.1478)	— (0.9698)	↓↓ (<0.0001)	↓↓ (<0.0001)
GF:5xFAD vs. SPF:5xFAD	↑ (0.5962)	↓ (0.2159)	— (0.921)	↓↓ (0.0601)	↓↓ (<0.0001)
GF:5xFAD vs. GF:WT	— (0.9996)	— (0.6786)	— (0.9675)	— (>0.9999)	↓ (0.5568)
SPF:5xFAD vs. SPF:WT	↓ (0.1542)	— (0.9704)	↑↑ (0.4428)	↓↓ (0.001)	↑ (0.0026)
GF:WT vs. SPF:5xFAD	↑ (0.5687)	↓ (0.0312)	↓ (0.7549)	↓↓ (0.088)	↓↓ (<0.0001)
GF:5xFAD vs. SPF:WT	↓ (0.555)	— (0.5594)	↑ (0.7601)	↓↓ (<0.0001)	↓↓ (<0.0001)
**Males:**					
GF:WT vs. SPF:WT	↓↓ (0.6975)	— (0.953)	— (0.9848)	↓ (0.4807)	↓ (0.2177)
GF:5xFAD vs. SPF:5xFAD	↑ (0.7291)	↑↑↑ (0.0254)	↑ (0.6673)	↓ (0.795)	↓↓ (0.0014)
GF:5xFAD vs. GF:WT	↑↑↑ (0.0693)	↑↑ (0.1612)	↑ (0.6598)	↓ (0.7661)	— (>0.9999)
SPF:5xFAD vs. SPF:WT	↑ (0.9023)	↓ (0.4729)	— (0.9561)	↓ (0.446)	↑↑↑ (0.0013)
GF:WT vs. SPF:5xFAD	↓↓ (0.4116)	↑ (0.8453)	— (0.9996)	— (>0.9999)	↓↓ (<0.0001)
GF:5xFAD vs. SPF:WT	↑↑ (0.2602)	↑↑ (0.2384)	↑↑ (0.2708)	↓↓ (0.059)	↓ (0.5256)

Change from mean: — No change; ↑ (≥20%); ↑↑ (≥50%); ↑↑↑ (≥100%); ↓ (≤20%); ↓↓ (≤50%); ↓↓↓ (≤100%); no data (n.d.)
ANOVA: (adjusted p-value)
GF Germ-free; DC Deep cervical lymph nodes; LI Large Intestine; MLN Mesenteric lymph nodes; SC Superficial cervical lymph nodes; SI Small intestine; SP Spleen; SPF Specific pathogen-free; WT wild-type

Table 1.9 – IL-17A MFI [5xFAD 5 Months]

Group Comparison	MLN	SP	SC	LI	SI
**Females:**					
GF:WT vs. SPF:WT	↓↓ (0.0046)	— (0.9538)	↓↓ (0.1716)	↓ (0.7855)	— (0.9995)
GF:5xFAD vs. SPF:5xFAD	↓↓ (0.0455)	↓ (0.0185)	↓↓ (<0.0001)	↓↓ (0.0017)	↓↓ (<0.0001)
GF:5xFAD vs. GF:WT	↓↓ (0.6866)	— (0.6361)	↓ (0.9533)	↓ (0.8754)	↓ (0.4821)
SPF:5xFAD vs. SPF:WT	↓ (0.1584)	↑ (0.1483)	↑↑ (0.0556)	↑↑ (0.269)	↑↑↑ (0.0001)
GF:WT vs. SPF:5xFAD	↓ (0.3387)	↓ (0.18)	↓↓ (0.0001)	↓↓ (0.0159)	↓↓ (<0.0001)
GF:5xFAD vs. SPF:WT	↓↓ (0.0004)	— (0.971)	↓↓ (0.0451)	↓ (0.3947)	↓ (0.5434)
**Males:**					
GF:WT vs. SPF:WT	↓ (0.8399)	↓ (0.2502)	↓↓ (0.2203)	↓↓ (0.2859)	↓ (0.9066)
GF:5xFAD vs. SPF:5xFAD	↑↑↑ (0.5184)	— (0.6364)	↑↑↑ (0.9733)	↓ (0.492)	↓↓ (0.5104)
GF:5xFAD vs. GF:WT	↑↑ (0.7561)	— (0.9543)	↑↑↑ (0.9798)	↑ (0.9914)	↓↓ (0.8439)
SPF:5xFAD vs. SPF:WT	↓↓ (0.6025)	— (>0.9999)	↓↓ (0.1498)	— (0.9997)	— (0.995)
GF:WT vs. SPF:5xFAD	↑ (0.9923)	↓ (0.367)	— (>0.9999)	↓↓ (0.3557)	↓ (0.8579)
GF:5xFAD vs. SPF:WT	— (0.9938)	— (0.5277)	↓↓ (0.3057)	↓ (0.4222)	↓↓ (0.5446)

Change from mean: — No change; ↑ (≥20%); ↑↑ (≥50%); ↑↑↑ (≥100%); ↓ (≤20%); ↓↓ (≤50%); ↓↓↓ (≤100%); no data (n.d.)
ANOVA: (adjusted p-value)
GF Germ-free; DC Deep cervical lymph nodes; LI Large Intestine; MLN Mesenteric lymph nodes; SC Superficial cervical lymph nodes; SI Small intestine; SP Spleen; SPF Specific pathogen-free; WT wild-type

Table 1.10 – IL-4⁺ T cells (% CD4⁺ T cells) [5xFAD 5 Months]

Group Comparison	MLN	SP	SC	LI	SI
**Females:**					
GF:WT vs. SPF:WT	↓↓ (0.0846)	↑↑↑ (0.8324)	↑↑↑ (0.1672)	↑↑ (0.6359)	↑↑ (0.9805)
GF:5xFAD vs. SPF:5xFAD	↑ (0.8847)	↓↓ (0.0044)	↓ (0.7341)	↑↑↑ (0.6654)	— (0.9684)
GF:5xFAD vs. GF:WT	↑↑ (0.5298)	— (0.9988)	↓ (0.7582)	↓ (0.7596)	↑↑↑ (0.2289)
SPF:5xFAD vs. SPF:WT	↓↓ (0.2654)	↑↑↑ (0.002)	↑↑↑ (0.182)	↓ (0.9223)	↑↑↑ (0.4732)
GF:WT vs. SPF:5xFAD	↓ (0.9693)	↓↓ (0.0043)	— (0.9985)	↑↑↑ (0.2454)	↓↓ (0.5843)
GF:5xFAD vs. SPF:WT	↓ (0.5071)	↑↑↑ (0.7541)	↑↑↑ (0.5305)	↑ (0.9764)	↑↑↑ (0.2047)
**Males:**					
GF:WT vs. SPF:WT	↓↓ (0.6531)	— (>0.9999)	↑ (0.6886)	↓↓ (0.2915)	↑ (0.9153)
GF:5xFAD vs. SPF:5xFAD	↑↑↑ (0.0188)	↑↑↑ (0.0823)	— (0.996)	↑↑↑ (0.0002)	— (>0.9999)
GF:5xFAD vs. GF:WT	↑↑↑ (0.0029)	↑↑↑ (0.1505)	— (0.9975)	↑↑↑ (0.0006)	↓↓ (0.6444)
SPF:5xFAD vs. SPF:WT	— (0.9988)	↓ (0.979)	↑ (0.646)	↓↓ (0.1592)	↓ (0.7861)
GF:WT vs. SPF:5xFAD	↓↓ (0.7832)	↑ (0.9915)	— (>0.9999)	↑ (0.996)	↑↑↑ (0.5353)
GF:5xFAD vs. SPF:WT	↑↑↑ (0.0108)	↑↑↑ (0.0882)	↑↑ (0.7149)	↑↑ (0.0129)	↓ (0.8518)

Change from mean: — No change; ↑ (≥20%); ↑↑ (≥50%); ↑↑↑ (≥100%); ↓ (≤20%); ↓↓ (≤50%); ↓↓↓ (≤100%); no data (n.d.)
ANOVA: (adjusted p-value)
GF Germ-free; DC Deep cervical lymph nodes; LI Large Intestine; MLN Mesenteric lymph nodes; SC Superficial cervical lymph nodes; SI Small intestine; SP Spleen; SPF Specific pathogen-free; WT wild-type

Table 1.11 – IL-4 MFI [5xFAD 5 Months]

Group Comparison	MLN	SP	SC	LI	SI
**Females:**					
GF:WT vs. SPF:WT	— (0.9385)	— (0.9539)	— (0.0542)	— (0.8077)	— (0.9289)
GF:5xFAD vs. SPF:5xFAD	↓ (<0.0001)	↓ (0.0006)	↓ (<0.0001)	↓ (<0.0001)	↓ (<0.0001)
GF:5xFAD vs. GF:WT	— (0.9998)	— (0.9905)	— (0.45)	— (0.9895)	— (0.7241)
SPF:5xFAD vs. SPF:WT	↑↑ (<0.0001)	↑↑ (0.0027)	↑ (0.0002)	↑↑ (<0.0001)	↑↑ (<0.0001)
GF:WT vs. SPF:5xFAD	↓ (<0.0001)	↓ (0.0023)	↓ (<0.0001)	↓ (<0.0001)	↓ (<0.0001)
GF:5xFAD vs. SPF:WT	— (0.9496)	— (0.9911)	— (0.4163)	— (0.9055)	— (0.9924)
**Males:**					
GF:WT vs. SPF:WT	↑ (0.3919)	↓ (0.5092)	— (>0.9999)	— (>0.9999)	↓ (0.735)
GF:5xFAD vs. SPF:5xFAD	↓↓ (<0.0001)	↓↓ (<0.0001)	↓↓ (<0.0001)	↓↓ (<0.0001)	↓↓ (0.0007)
GF:5xFAD vs. GF:WT	↓↓ (0.0548)	↓↓ (0.5681)	↓↓ (0.1895)	↓↓ (0.3381)	↓↓ (0.3329)
SPF:5xFAD vs. SPF:WT	↑↑↑ (0.0003)	↑↑ (0.0174)	↑↑ (0.0045)	↑↑↑ (0.0002)	↑↑ (0.0345)
GF:WT vs. SPF:5xFAD	↓ (0.0847)	↓↓ (0.0033)	↓ (0.0184)	↓↓ (0.0025)	↓↓ (0.0164)
GF:5xFAD vs. SPF:WT	↓ (0.5061)	↓↓ (0.0251)	↓↓ (0.1242)	↓↓ (0.069)	↓↓ (0.0406)

Change from mean: — No change; ↑ (≥20%); ↑↑ (≥50%); ↑↑↑ (≥100%); ↓ (≤20%); ↓↓ (≤50%); ↓↓↓ (≤100%); no data (n.d.)
ANOVA: (adjusted p-value)
GF Germ-free; DC Deep cervical lymph nodes; LI Large Intestine; MLN Mesenteric lymph nodes; SC Superficial cervical lymph nodes; SI Small intestine; SP Spleen; SPF Specific pathogen-free; WT wild-type

Table 1.12 – GM-CSF⁺ T cells (% CD4⁺ T cells) [5xFAD 5 Months]

Group Comparison	MLN	SP	SC	LI	SI
**Females:**					
GF:WT vs. SPF:WT	— (0.9431)	↓ (0.9371)	↓ (0.7237)	↑↑ (0.4387)	↓ (0.5414)
GF:5xFAD vs. SPF:5xFAD	↑↑↑ (0.2303)	↑↑↑ (0.1435)	↑↑↑ (0.1562)	↑↑ (0.1013)	↑↑↑ (<0.0001)
GF:5xFAD vs. GF:WT	— (0.9952)	↑↑ (0.262)	↑↑↑ (0.4734)	↑ (0.5284)	↑↑ (0.0138)
SPF:5xFAD vs. SPF:WT	↓↓ (0.2573)	↓ (0.8467)	↓↓ (0.3917)	↑ (0.928)	↓↓ (0.0106)
GF:WT vs. SPF:5xFAD	↑↑↑ (0.3308)	— (0.9909)	↑↑↑ (0.8819)	↑ (0.6779)	↑↑↑ (0.0514)
GF:5xFAD vs. SPF:WT	— (0.9814)	↑ (0.8126)	— (0.9997)	↑↑↑ (0.0762)	↑ (0.5928)
**Males:**					
GF:WT vs. SPF:WT	↓ (0.7113)	↑↑↑ (0.5284)	— (0.981)	↑ (0.9913)	↓ (0.9822)
GF:5xFAD vs. SPF:5xFAD	↑ (0.9044)	↑↑↑ (0.3663)	↑↑↑ (0.0352)	↓ (0.7669)	↑↑↑ (0.0827)
GF:5xFAD vs. GF:WT	↑ (0.9686)	↑ (0.9803)	↑↑↑ (0.2149)	— (0.9964)	↑↑↑ (0.0899)
SPF:5xFAD vs. SPF:WT	↓ (0.5236)	— (>0.9999)	↓↓ (0.6317)	↑↑ (0.6736)	↓ (0.9034)
GF:WT vs. SPF:5xFAD	— (0.9983)	↑↑↑ (0.6146)	↑↑ (0.9209)	↓ (0.8825)	↑↑ (0.9859)
GF:5xFAD vs. SPF:WT	↓ (0.9096)	↑↑↑ (0.2624)	↑↑ (0.1931)	— (>0.9999)	↑↑↑ (0.1013)

Change from mean: — No change; ↑ (≥20%); ↑↑ (≥50%); ↑↑↑ (≥100%); ↓ (≤20%); ↓↓ (≤50%); ↓↓↓ (≤100%); no data (n.d.)
ANOVA: (adjusted p-value)
GF Germ-free; DC Deep cervical lymph nodes; LI Large Intestine; MLN Mesenteric lymph nodes; SC Superficial cervical lymph nodes; SI Small intestine; SP Spleen; SPF Specific pathogen-free; WT wild-type

Table 1.13 – GM-CSF MFI [5xFAD 5 Months]

Group Comparison	MLN	SP	SC	LI	SI
**Females:**					
GF:WT vs. SPF:WT	— (0.9796)	— (0.9765)	— (0.9995)	↑ (0.6084)	↑ (0.5666)
GF:5xFAD vs. SPF:5xFAD	↓ (0.0395)	— (0.9622)	↓ (0.0179)	↓ (0.8444)	↓ (0.0418)
GF:5xFAD vs. GF:WT	— (0.7953)	— (0.9577)	— (0.8569)	— (0.826)	— (0.6901)
SPF:5xFAD vs. SPF:WT	↑ (0.1826)	— (0.9798)	↑↑ (0.1637)	↑↑ (0.6424)	↑↑ (0.0497)
GF:WT vs. SPF:5xFAD	↓ (0.2196)	— (0.8059)	↓ (0.1096)	— (0.9995)	↓ (0.3186)
GF:5xFAD vs. SPF:WT	— (0.9846)	— (>0.9999)	— (0.9486)	↑ (0.938)	— (0.97)
**Males:**					
GF:WT vs. SPF:WT	↓ (0.1095)	— (0.9246)	↓↓ (0.2276)	↓ (0.4618)	— (0.9654)
GF:5xFAD vs. SPF:5xFAD	↑↑ (0.0125)	↑ (0.5209)	↑↑ (0.2019)	— (0.933)	↑ (0.7431)
GF:5xFAD vs. GF:WT	↑↑ (0.0149)	↑ (0.6874)	↑↑↑ (0.1034)	— (0.9988)	— (0.9742)
SPF:5xFAD vs. SPF:WT	↓ (0.1044)	— (0.9884)	↓ (0.4253)	↓ (0.1325)	↓ (0.6715)
GF:WT vs. SPF:5xFAD	— (0.9996)	— (0.9935)	↓ (0.96)	— (0.9866)	— (0.88)
GF:5xFAD vs. SPF:WT	— (0.621)	↑ (0.2373)	— (0.8866)	↓ (0.2929)	— (0.9997)

Change from mean: — No change; ↑ (≥20%); ↑↑ (≥50%); ↑↑↑ (≥100%); ↓ (≤20%); ↓↓ (≤50%); ↓↓↓ (≤100%); no data (n.d.)
ANOVA: (adjusted p-value)
GF Germ-free; DC Deep cervical lymph nodes; LI Large Intestine; MLN Mesenteric lymph nodes; SC Superficial cervical lymph nodes; SI Small intestine; SP Spleen; SPF Specific pathogen-free; WT wild-type

Table 2.1 – CD4$^+$ T cells (% of CD45$^+$) [3xTg 7 Months]

Group Comparison	MLN	SP	SC	DC
**Females:**				
GF:WT vs. SPF:WT	↓ (0.1962)	— (0.9972)	— (0.6441)	— (0.9081)
GF:3xTg vs. SPF:3xTg	— (0.3619)	↑ (0.0634)	— (0.3712)	— (0.8905)
GF:3xTg vs. GF:WT	↑↑ (<0.0001)	↑ (0.0666)	↑↑ (<0.0001)	↑↑ (0.0004)
SPF:3xTg vs. SPF:WT	↑ (0.0864)	— (0.9988)	↑ (<0.0001)	↑↑ (0.0003)
GF:WT vs. SPF:3xTg	↓ (0.0002)	— (>0.9999)	↓ (<0.0001)	↓ (0.001)
GF:3xTg vs. SPF:WT	↑ (0.0089)	↑ (0.0978)	↑↑ (<0.0001)	↑↑ (0.0001)
**Males:**				
GF:WT vs. SPF:WT	↓ (0.3012)	↓ (0.1523)	— (0.9917)	— (0.9933)
GF:3xTg vs. SPF:3xTg	— (0.9642)	— (0.842)	— (0.8199)	— (0.9883)
GF:3xTg vs. GF:WT	— (0.9983)	↓ (0.0237)	— (0.8071)	— (0.9563)
SPF:3xTg vs. SPF:WT	↓ (0.5843)	↓↓ (<0.0001)	— (0.9879)	— (0.9723)
GF:WT vs. SPF:3xTg	— (0.9855)	↑↑ (0.0071)	— (0.9999)	— (0.998)
GF:3xTg vs. SPF:WT	↓ (0.2751)	↓ (0.0001)	— (0.9451)	↑ (0.8705)

Change from mean: — No change; ↑ (≥20%); ↑↑ (≥50%); ↑↑↑ (≥100%); ↓ (≤20%); ↓↓ (≤50%); ↓↓↓ (≤100%); no data (n.d.)
ANOVA: (adjusted p-value)
GF Germ-free; DC Deep cervical lymph nodes; LI Large Intestine; MLN Mesenteric lymph nodes; SC Superficial cervical lymph nodes; SI Small intestine; SP Spleen; SPF Specific pathogen-free; WT wild-type

Table 2.2 – CD4$^+$ T cell Counts [3xTg 7 Months]

Group Comparison	MLN	SP	SC	DC
**Females:**				
GF:WT vs. SPF:WT	↑ (0.9066)	— (0.9092)	↓ (0.1299)	↑↑↑ (0.6635)
GF:3xTg vs. SPF:3xTg	↓ (0.4802)	— (0.5712)	— (0.2124)	— (0.9672)
GF:3xTg vs. GF:WT	↑↑ (0.2604)	↑ (0.1767)	↑↑ (0.0097)	↑↑↑ (0.2027)
SPF:3xTg vs. SPF:WT	↑↑↑ (0.0029)	↑↑ (0.0013)	↑ (0.0097)	↑↑↑ (0.0428)
GF:WT vs. SPF:3xTg	↓↓ (0.0049)	↓ (0.0068)	↓↓ (<0.0001)	↓ (0.3334)
GF:3xTg vs. SPF:WT	↑↑↑ (0.123)	↑ (0.0504)	— (0.5786)	↑↑↑ (0.0256)
**Males:**				
GF:WT vs. SPF:WT	— (0.9989)	↓ (0.8764)	— (0.7691)	— (0.9694)
GF:3xTg vs. SPF:3xTg	↓↓ (0.0031)	↑↑ (0.0407)	— (0.9167)	— (0.9769)
GF:3xTg vs. GF:WT	— (0.9978)	↑↑↑ (0.0001)	↓ (0.6883)	— (0.9999)
SPF:3xTg vs. SPF:WT	↑↑↑ (0.0118)	↑ (0.7518)	↓ (0.6298)	↑ (0.8767)
GF:WT vs. SPF:3xTg	↓↓ (0.0029)	↓ (0.3095)	— (0.9854)	— (0.9889)
GF:3xTg vs. SPF:WT	— (0.9907)	↑↑↑ (0.002)	↓ (0.212)	— (0.9762)

Change from mean: — No change; ↑ (≥20%); ↑↑ (≥50%); ↑↑↑ (≥100%); ↓ (≤20%); ↓↓ (≤50%); ↓↓↓ (≤100%); no data (n.d.)
ANOVA: (adjusted p-value)
GF Germ-free; DC Deep cervical lymph nodes; LI Large Intestine; MLN Mesenteric lymph nodes; SC Superficial cervical lymph nodes; SI Small intestine; SP Spleen; SPF Specific pathogen-free; WT wild-type

Table 2.3 – T cell Counts [3xTg 7 Months]

Group Comparison	MLN	SP	SC	DC
**Females:**				
GF:WT vs. SPF:WT	↑↑ (0.7518)	— (0.9163)	↓ (0.1173)	↑↑↑ (0.3193)
GF:3xTg vs. SPF:3xTg	↓ (0.4379)	— (0.6671)	↓ (0.1795)	↓↓ (0.0227)
GF:3xTg vs. GF:WT	↑ (0.7715)	— (0.607)	↑ (0.3155)	— (0.9472)
SPF:3xTg vs. SPF:WT	↑↑↑ (0.0099)	↑ (0.019)	— (0.3569)	↑↑↑ (0.0016)
GF:WT vs. SPF:3xTg	↓ (0.0438)	↓ (0.0807)	↓ (0.0022)	↓ (0.0766)
GF:3xTg vs. SPF:WT	↑↑↑ (0.3093)	↑ (0.2705)	— (0.9618)	↑↑ (0.6081)
**Males:**				
GF:WT vs. SPF:WT	— (0.9997)	— (0.949)	— (0.6402)	— (0.9061)
GF:3xTg vs. SPF:3xTg	↓↓ (0.005)	↑↑ (0.2512)	— (0.9739)	— (0.997)
GF:3xTg vs. GF:WT	↓ (0.9252)	↑↑↑ (0.0142)	↓ (0.1437)	↓ (0.5391)
SPF:3xTg vs. SPF:WT	↑↑↑ (0.0352)	— (0.96)	↓ (0.0739)	— (0.984)
GF:WT vs. SPF:3xTg	↓↓ (0.0118)	↓ (0.7309)	↑ (0.417)	↑ (0.7052)
GF:3xTg vs. SPF:WT	↓ (0.9234)	↑↑ (0.0772)	↓ (0.0149)	— (0.9399)

Change from mean: — No change; ↑ (≥20%); ↑↑ (≥50%); ↑↑↑ (≥100%); ↓ (≤20%); ↓↓ (≤50%); ↓↓↓ (≤100%); no data (n.d.)
ANOVA: (adjusted p-value)
GF Germ-free; DC Deep cervical lymph nodes; LI Large Intestine; MLN Mesenteric lymph nodes; SC Superficial cervical lymph nodes; SI Small intestine; SP Spleen; SPF Specific pathogen-free; WT wild-type

Table 2.4 – B cell Counts [3xTg 7 Months]

Group Comparison	MLN	SP	SC	DC
**Females:**				
GF:WT vs. SPF:WT	↑↑↑ (0.0358)	— (0.217)	↓ (0.1436)	↑↑ (0.5367)
GF:3xTg vs. SPF:3xTg	↓↓ (0.0183)	↓ (<0.0001)	↓ (0.0581)	↓ (0.6906)
GF:3xTg vs. GF:WT	↓ (0.1825)	— (0.7587)	↓ (0.0063)	↓ (0.6261)
SPF:3xTg vs. SPF:WT	↑↑↑ (0.0026)	↑↑ (<0.0001)	↓ (0.0108)	↑↑ (0.595)
GF:WT vs. SPF:3xTg	— (0.6227)	↓ (<0.0001)	— (0.6723)	— (0.9974)
GF:3xTg vs. SPF:WT	↑ (0.8577)	— (0.7877)	↓↓ (<0.0001)	— (0.9969)
**Males:**				
GF:WT vs. SPF:WT	↑↑ (0.6873)	— (>0.9999)	— (0.9958)	— (>0.9999)
GF:3xTg vs. SPF:3xTg	↓↓ (<0.0001)	↑ (0.0947)	↓ (0.0895)	↓ (0.3266)
GF:3xTg vs. GF:WT	— (>0.9999)	↑↑↑ (<0.0001)	↓ (0.6655)	— (0.9874)
SPF:3xTg vs. SPF:WT	↑↑↑ (<0.0001)	↑↑↑ (0.0003)	↑ (0.6591)	↑ (0.6066)
GF:WT vs. SPF:3xTg	↓↓ (<0.0001)	↓↓ (0.0001)	↓ (0.4883)	↓ (0.5579)
GF:3xTg vs. SPF:WT	↑↑ (0.7458)	↑↑↑ (<0.0001)	↓ (0.5722)	— (0.9871)

Change from mean: — No change; ↑ (≥20%); ↑↑ (≥50%); ↑↑↑ (≥100%); ↓ (≤20%); ↓↓ (≤50%); ↓↓↓ (≤100%); no data (n.d.)
ANOVA: (adjusted p-value)
GF Germ-free; DC Deep cervical lymph nodes; LI Large Intestine; MLN Mesenteric lymph nodes; SC Superficial cervical lymph nodes; SI Small intestine; SP Spleen; SPF Specific pathogen-free; WT wild-type

Table 2.5 – Foxp3^{+} T cells (% CD4^{+} T cells) [3xTg 12 Months]

Group Comparison	MLN	SP	SC	DC
**Females:**				
GF:WT vs. SPF:WT	↑↑ (0.2773)	— (0.9996)	↑↑ (0.2551)	↑ (0.7687)
GF:3xTg vs. SPF:3xTg	↑↑↑ (0.0007)	↑↑ (0.002)	↑↑ (0.0077)	↑ (0.3048)
GF:3xTg vs. GF:WT	— (0.9926)	↑ (0.2206)	— (0.7668)	— (0.9205)
SPF:3xTg vs. SPF:WT	↓↓ (0.2829)	↓ (0.3365)	— (0.9996)	— (0.975)
GF:WT vs. SPF:3xTg	↑↑↑ (0.0026)	↑ (0.2144)	↑↑ (0.1733)	↑ (0.8984)
GF:3xTg vs. SPF:WT	↑↑ (0.1559)	↑ (0.2525)	↑↑ (0.0293)	↑↑ (0.2245)
**Males:**				
GF:WT vs. SPF:WT	↑↑↑ (0.2291)	— (0.983)	— (0.921)	↑ (0.5114)
GF:3xTg vs. SPF:3xTg	↑↑↑ (0.0356)	↑↑↑ (<0.0001)	↑ (0.3177)	↑↑ (0.0201)
GF:3xTg vs. GF:WT	↑ (0.4307)	↑↑ (0.0102)	↑ (0.0634)	↑↑ (0.0065)
SPF:3xTg vs. SPF:WT	↑ (0.9181)	↓ (0.1662)	↑ (0.2674)	↑↑ (0.3181)
GF:WT vs. SPF:3xTg	↑↑ (0.2919)	↑↑ (0.0613)	— (0.6308)	— (0.9692)
GF:3xTg vs. SPF:WT	↑↑↑ (0.0371)	↑↑ (0.0077)	↑↑ (0.0212)	↑↑↑ (0.0006)

Change from mean: — No change; ↑ (≥20%); ↑↑ (≥50%); ↑↑↑ (≥100%); ↓ (≤20%); ↓↓ (≤50%); ↓↓↓ (≤100%); no data (n.d.)
ANOVA: (adjusted p-value)
GF Germ-free; DC Deep cervical lymph nodes; LI Large Intestine; MLN Mesenteric lymph nodes; SC Superficial cervical lymph nodes; SI Small intestine; SP Spleen; SPF Specific pathogen-free; WT wild-type

Table 2.6 – IFNγ⁺ T cells (% CD4⁺ T cells) [3xTg 12 Months]

Group Comparison	MLN	SP	SC	DC
**Females:**				
GF:WT vs. SPF:WT	— (0.9762)	↓ (0.1221)	— (0.9556)	— (>0.9999)
GF:3xTg vs. SPF:3xTg	— (>0.9999)	↑↑↑ (0.0027)	— (0.8784)	— (0.9785)
GF:3xTg vs. GF:WT	↑ (0.8876)	— (0.9959)	↑ (0.8537)	↑ (0.858)
SPF:3xTg vs. SPF:WT	— (0.998)	↓↓ (<0.0001)	— (0.9743)	— (0.9195)
GF:WT vs. SPF:3xTg	— (0.9177)	↑↑↑ (0.0064)	— (0.9988)	— (0.9584)
GF:3xTg vs. SPF:WT	— (0.9961)	↓ (0.1588)	— (0.9981)	↑ (0.7615)
**Males:**				
GF:WT vs. SPF:WT	— (>0.9999)	↓↓ (0.0419)	↓ (0.9653)	— (>0.9999)
GF:3xTg vs. SPF:3xTg	— (0.9775)	↓ (0.8548)	↓ (0.3177)	↓ (0.1722)
GF:3xTg vs. GF:WT	↑↑↑ (0.0893)	↑↑ (0.6405)	↑↑↑ (0.0969)	↑↑↑ (0.103)
SPF:3xTg vs. SPF:WT	↑↑↑ (0.2546)	— (0.9795)	↑↑↑ (<0.0001)	↑↑↑ (<0.0001)
GF:WT vs. SPF:3xTg	↓↓ (0.0775)	↓ (0.1256)	↓↓ (<0.0001)	↓↓ (<0.0001)
GF:3xTg vs. SPF:WT	↑↑↑ (0.2155)	↓ (0.6483)	↑↑↑ (0.1713)	↑↑↑ (0.1255)

Change from mean: — No change; ↑ (≥20%); ↑↑ (≥50%); ↑↑↑ (≥100%); ↓ (≤20%); ↓↓ (≤50%); ↓↓↓ (≤100%); no data (n.d.)
ANOVA: (adjusted p-value)
GF Germ-free; DC Deep cervical lymph nodes; LI Large Intestine; MLN Mesenteric lymph nodes; SC Superficial cervical lymph nodes; SI Small intestine; SP Spleen; SPF Specific pathogen-free; WT wild-type

Table 2.7 – IFNγ MFI [3xTg 12 Months]

Group Comparison	MLN	SP	SC	DC
**Females:**				
GF:WT vs. SPF:WT	↓ (0.0496)	— (0.3142)	— (0.0594)	↓↓ (0.0014)
GF:3xTg vs. SPF:3xTg	↑ (0.046)	↑↑ (0.0002)	↑ (0.0051)	↑↑ (0.0229)
GF:3xTg vs. GF:WT	— (0.7935)	— (0.9976)	— (0.9673)	↑↑ (0.0774)
SPF:3xTg vs. SPF:WT	↓ (0.0011)	↓↓ (<0.0001)	↓ (<0.0001)	↓↓ (0.0001)
GF:WT vs. SPF:3xTg	↑ (0.3126)	↑↑ (0.0005)	↑ (0.049)	— (0.9788)
GF:3xTg vs. SPF:WT	— (0.1925)	— (0.3747)	— (0.0746)	↓ (0.0309)
**Males:**				
GF:WT vs. SPF:WT	↑↑ (0.1876)	↓ (0.0001)	— (0.5808)	— (0.2016)
GF:3xTg vs. SPF:3xTg	↑↑ (0.285)	— (0.7805)	— (0.6919)	— (0.8703)
GF:3xTg vs. GF:WT	— (0.9836)	— (>0.9999)	— (0.8423)	— (0.9525)
SPF:3xTg vs. SPF:WT	↑ (0.8871)	↓ (0.0064)	— (0.3642)	— (0.9753)
GF:WT vs. SPF:3xTg	↑ (0.2707)	— (0.7004)	— (0.9838)	— (0.3917)
GF:3xTg vs. SPF:WT	↑↑ (0.1825)	↓ (0.0012)	— (0.9994)	— (0.6914)

Change from mean: — No change; ↑ (≥20%); ↑↑ (≥50%); ↑↑↑ (≥100%); ↓ (≤20%); ↓↓ (≤50%); ↓↓↓ (≤100%); no data (n.d.)
ANOVA: (adjusted p-value)
GF Germ-free; DC Deep cervical lymph nodes; LI Large Intestine; MLN Mesenteric lymph nodes; SC Superficial cervical lymph nodes; SI Small intestine; SP Spleen; SPF Specific pathogen-free; WT wild-type

Table 2.8 – IL-17A$^+$ T cells (% CD4$^+$ T cells) [3xTg 12 Months]

Group Comparison	MLN	SP	SC	DC
**Females:**				
GF:WT vs. SPF:WT	↓↓ (0.0009)	↓↓ (0.0026)	↓↓ (0.1327)	↓ (0.9984)
GF:3xTg vs. SPF:3xTg	↓ (0.2435)	↓ (0.9645)	— (0.9938)	↓ (0.9131)
GF:3xTg vs. GF:WT	↑↑↑ (0.6584)	— (>0.9999)	↑↑↑ (0.4784)	↑↑↑ (0.8342)
SPF:3xTg vs. SPF:WT	↓ (0.3045)	↓↓ (0.0053)	↓ (0.5256)	↑↑↑ (0.5367)
GF:WT vs. SPF:3xTg	↓↓ (0.0361)	↓ (0.9717)	↓↓ (0.6294)	↓↓ (0.5591)
GF:3xTg vs. SPF:WT	↓↓ (0.0071)	↓↓ (0.0019)	↓ (0.6361)	↑↑ (0.8589)
**Males:**				
GF:WT vs. SPF:WT	↓↓ (0.0008)	↓↓ (0.0266)	↓ (0.9928)	↓↓ (0.8846)
GF:3xTg vs. SPF:3xTg	↓↓ (0.0048)	— (>0.9999)	↓↓ (0.3737)	↓↓ (0.3167)
GF:3xTg vs. GF:WT	↑↑↑ (0.8465)	— (0.9902)	↑↑↑ (0.72)	↑↑↑ (0.9974)
SPF:3xTg vs. SPF:WT	— (0.985)	↓ (0.0908)	↑↑↑ (0.0095)	↑↑↑ (0.2374)
GF:WT vs. SPF:3xTg	↓↓ (<0.0001)	— (0.9927)	↓↓ (0.0064)	↓↓ (0.0475)
GF:3xTg vs. SPF:WT	↓↓ (0.0134)	↓ (0.1562)	↑↑↑ (0.8188)	↓↓ (0.9879)

Change from mean: — No change; ↑ (≥20%); ↑↑ (≥50%); ↑↑↑ (≥100%); ↓ (≤20%); ↓↓ (≤50%); ↓↓↓ (≤100%); no data (n.d.)
ANOVA: (adjusted p-value)
GF Germ-free; DC Deep cervical lymph nodes; LI Large Intestine; MLN Mesenteric lymph nodes; SC Superficial cervical lymph nodes; SI Small intestine; SP Spleen; SPF Specific pathogen-free; WT wild-type

Table 2.9 – IL-17A MFI [3xTg 12 Months]

Group Comparison	MLN	SP	SC	DC
**Females:**				
GF:WT vs. SPF:WT	— (0.9972)	↓ (0.0448)	↓ (0.0387)	↓ (0.4814)
GF:3xTg vs. SPF:3xTg	— (0.2874)	↑ (0.4775)	↑↑↑ (<0.0001)	— (0.9592)
GF:3xTg vs. GF:WT	↓ (0.1466)	↑ (0.6517)	↑↑ (0.0344)	— (0.9965)
SPF:3xTg vs. SPF:WT	— (0.8843)	↓ (0.0274)	↓↓ (0.0002)	↓ (0.2633)
GF:WT vs. SPF:3xTg	— (0.8414)	— (0.987)	↑ (0.6184)	— (0.9964)
GF:3xTg vs. SPF:WT	↓ (0.1077)	↓ (0.2825)	— (0.9987)	↓ (0.4315)
**Males:**				
GF:WT vs. SPF:WT	↓↓ (0.2883)	↓↓ (<0.0001)	↓↓ (0.0001)	↓↓ (0.0025)
GF:3xTg vs. SPF:3xTg	— (0.9999)	↓ (0.8354)	↓↓ (0.0125)	↓↓ (0.0002)
GF:3xTg vs. GF:WT	— (0.9994)	— (0.9963)	— (0.9921)	↓ (0.9469)
SPF:3xTg vs. SPF:WT	↓↓ (0.3057)	↓↓ (<0.0001)	— (0.998)	↑ (0.3206)
GF:WT vs. SPF:3xTg	— (>0.9999)	↓ (0.6242)	↓↓ (0.0003)	↓↓ (<0.0001)
GF:3xTg vs. SPF:WT	↓ (0.4386)	↓↓ (<0.0001)	↓↓ (0.0081)	↓↓ (0.0046)

Change from mean: — No change; ↑ (≥20%); ↑↑ (≥50%); ↑↑↑ (≥100%); ↓ (≤20%); ↓↓ (≤50%); ↓↓↓ (≤100%); no data (n.d.)
ANOVA: (adjusted p-value)
GF Germ-free; DC Deep cervical lymph nodes; LI Large Intestine; MLN Mesenteric lymph nodes; SC Superficial cervical lymph nodes; SI Small intestine; SP Spleen; SPF Specific pathogen-free; WT wild-type

Representative Flow Cytometry Plots

Genotype and microbiome shape immunity in a sex-specific manner in mouse models of Alzheimer's disease

John W. Bostick, T. Jaymie Connerly, Taren Thron, Brittany D. Needham, Matheus de Castro Fonseca, Rima Kaddurah-Daouk, Rob Knight, and Sarkis K. Mazmanian

Abbreviations:

5M	5 months
7M	7 months
8M	8 months
12M	12 months
15M	15 months
GF	Germ- free
DC	Deep cervical lymph nodes
LI	Large Intestine
MLN	Mesenteric lymph nodes
SC	Superficial cervical lymph nodes
SI	Small intestine
SP	Spleen
SPF	Specific pathogen- free

Representative Flow Cytometry Plots for Fig. 1, S2: CD4⁺ and CD8⁺ T cells [3xTg]

3xTg 7M Females: SP
$CD45^+CD3^+TCR\beta^+$

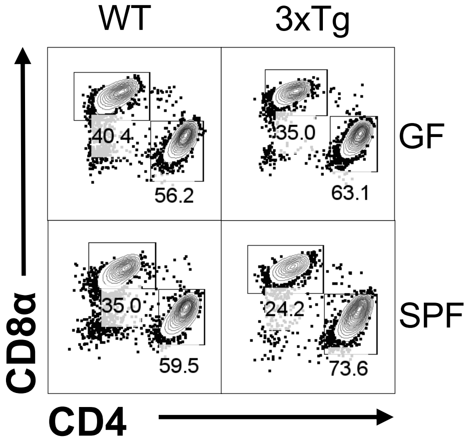

3xTg 7M Females: MLN
$CD45^+CD3^+TCR\beta^+$

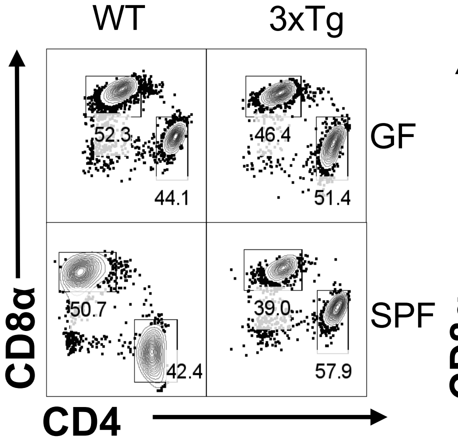

3xTg 7M Females: SC
$CD45^+CD3^+TCR\beta^+$

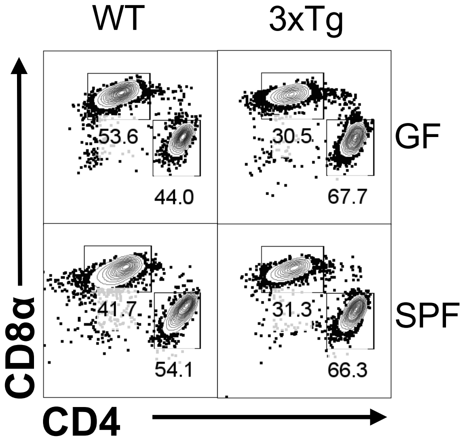

3xTg 7M Females: DC
$CD45^+CD3^+TCR\beta^+$

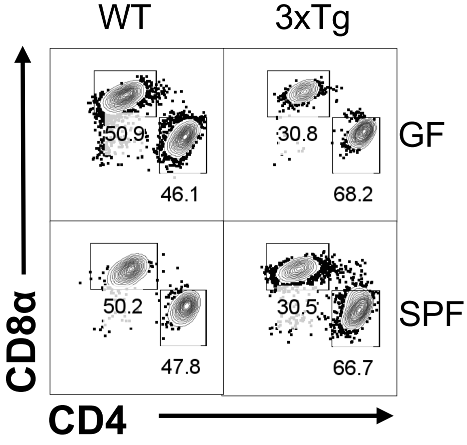

3xTg 7M Males: SP
$CD45^+CD3^+TCR\beta^+$

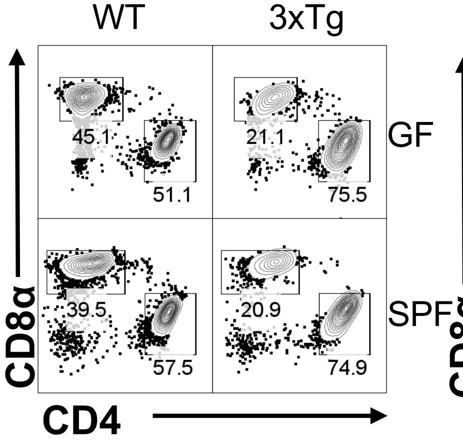

3xTg 7M Males: MLN
$CD45^+CD3^+TCR\beta^+$

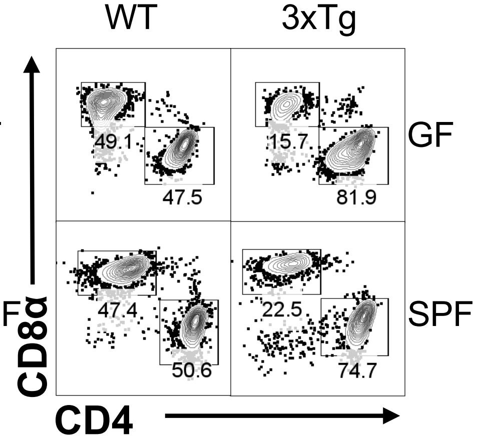

3xTg 7M Males: SC
$CD45^+CD3^+TCR\beta^+$

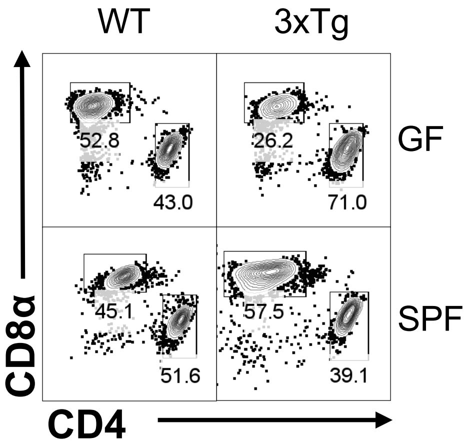

3xTg 7M Males: DC
$CD45^+CD3^+TCR\beta^+$

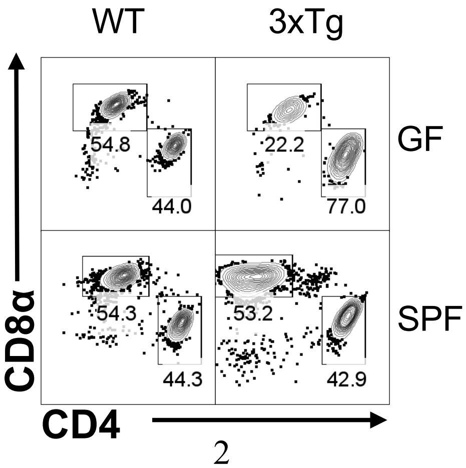

Representative Flow Cytometry Plots for Fig. 2, S2: CD4^{+} and CD8^{+} T cells [5xFAD]

5xFAD 5M Females: SP
CD45^{+}CD3^{+}TCRβ^{+}

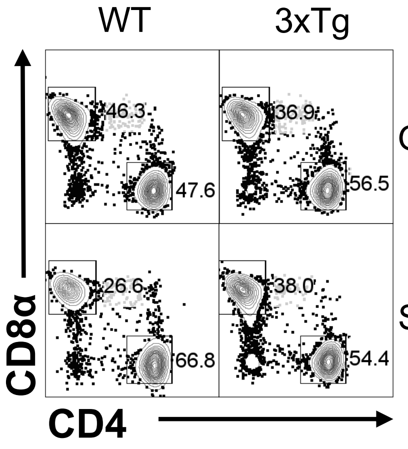

5xFAD 5M Females: MLN
CD45^{+}CD3^{+}TCRβ^{+}

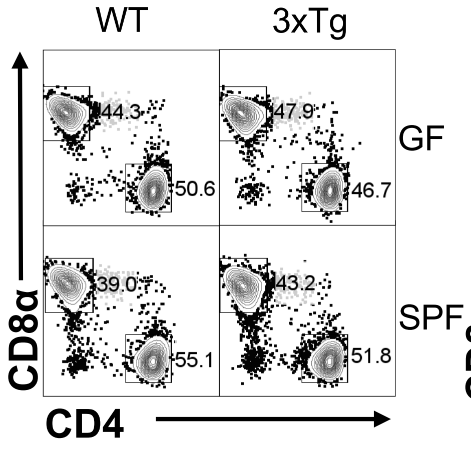

5xFAD 5M Females: SC
CD45^{+}CD3^{+}TCRβ^{+}

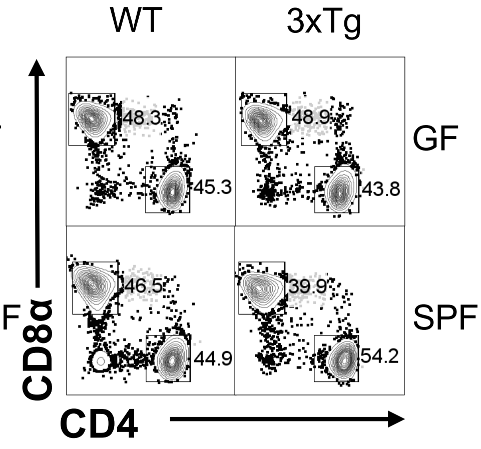

5xFAD 5M Females: DC
CD45^{+}CD3^{+}TCRβ^{+}

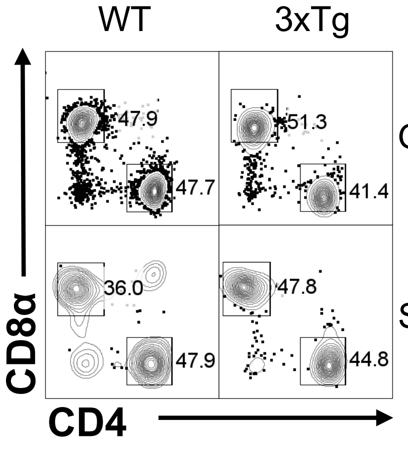

5xFAD 5M Males: SP
CD45^{+}CD3^{+}TCRβ^{+}

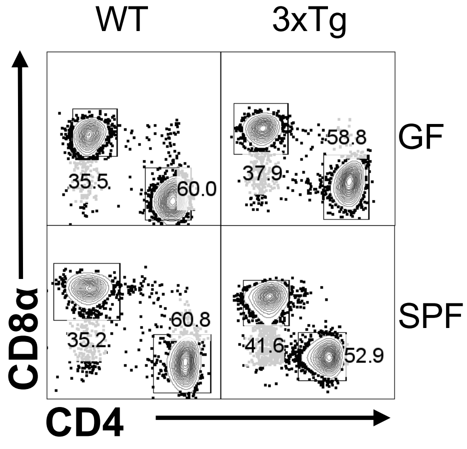

5xFAD 5M Males: MLN
CD45^{+}CD3^{+}TCRβ^{+}

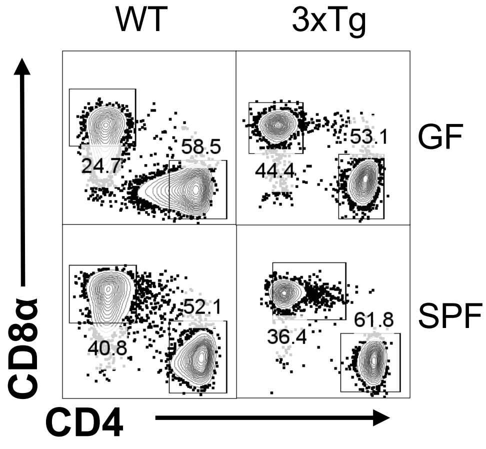

5xFAD 5M Males: SC
CD45^{+}CD3^{+}TCRβ^{+}

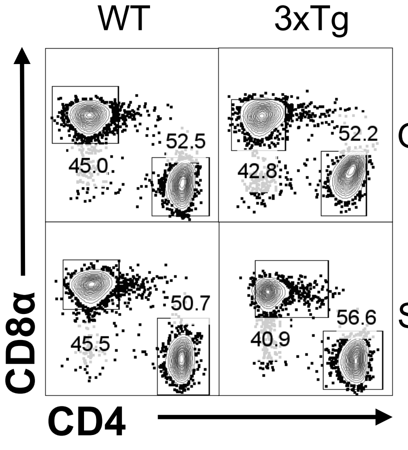

5xFAD 5M Males: DC
CD45^{+}CD3^{+}TCRβ^{+}

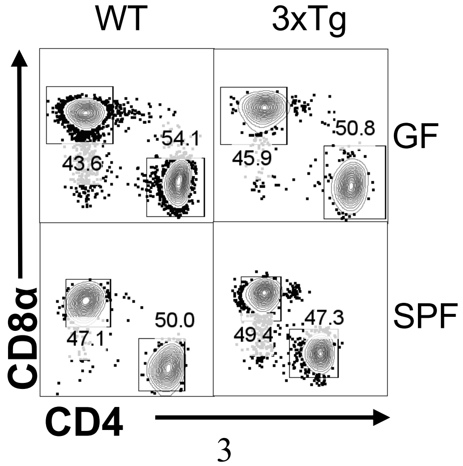

Representative Flow Cytometry Plots for Fig. 3, S4: IFNγ⁺/IL-17A⁺ T cells [3xTg]

3xTg 12M Females: SP
CD45⁺CD4⁺TCRβ⁺Foxp3⁻

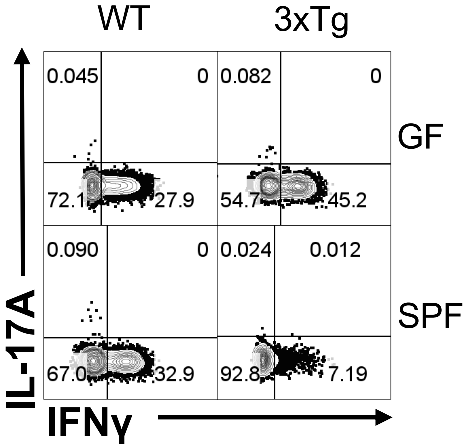

3xTg 12M Females: MLN
CD45⁺CD4⁺TCRβ⁺Foxp3⁻

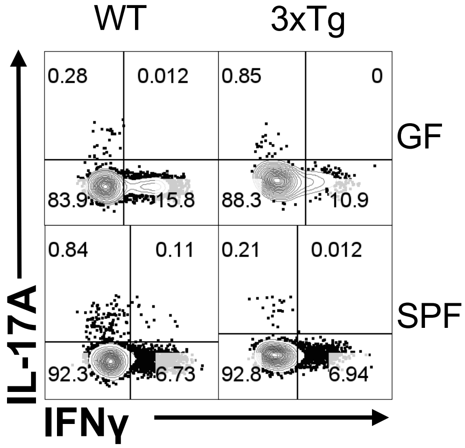

3xTg 12M Females: SC
CD45⁺CD4⁺TCRβ⁺Foxp3⁻

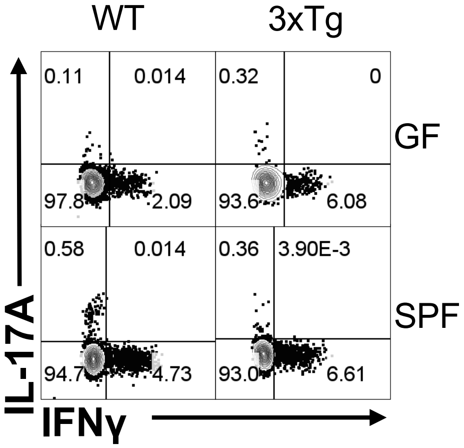

3xTg 12M Females: DC
CD45⁺CD4⁺TCRβ⁺Foxp3⁻

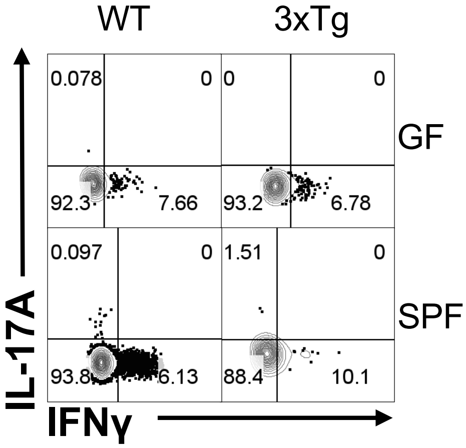

3xTg 12M Males: SP
CD45⁺CD4⁺TCRβ⁺Foxp3⁻

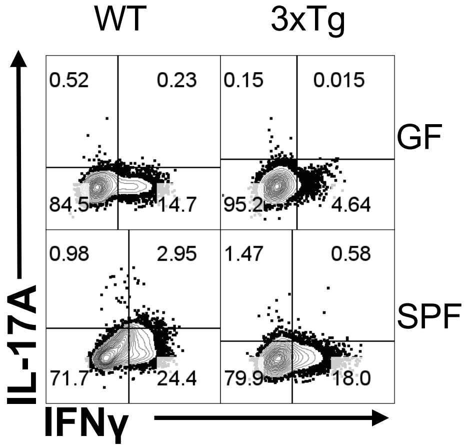

3xTg 12M Males: MLN
CD45⁺CD4⁺TCRβ⁺Foxp3⁻

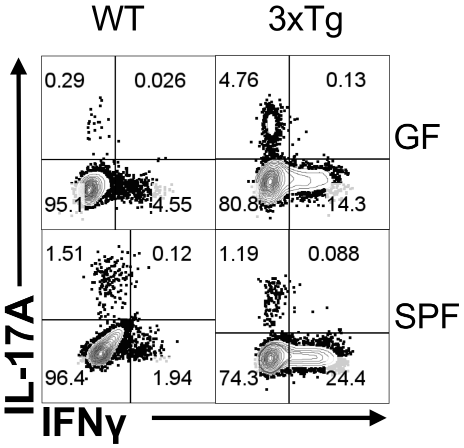

3xTg 7M Males: SC
CD45⁺CD4⁺TCRβ⁺Foxp3⁻

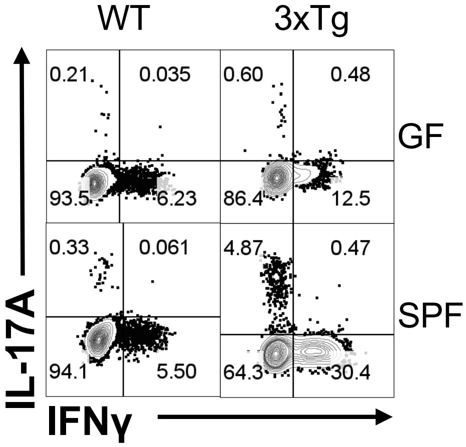

3xTg 7M Males: DC
CD45⁺CD4⁺TCRβ⁺Foxp3⁻

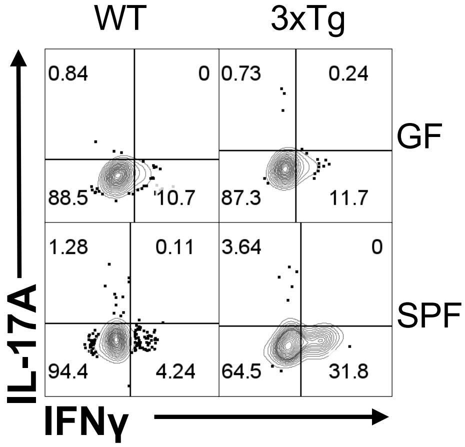

Representative Flow Cytometry Plots for Fig. 3: IL-17A MFI [3xTg]

3xTg 12M Females: SP
CD45$^+$CD4$^+$TCRβ$^+$Foxp3$^-$

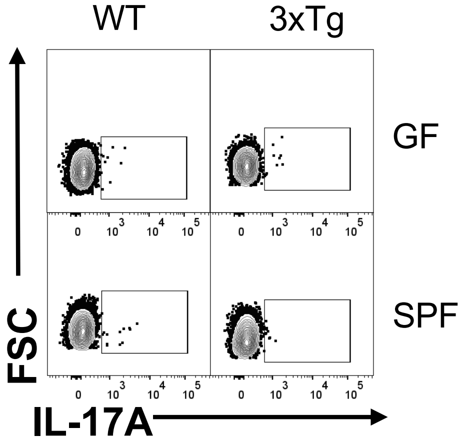

3xTg 12M Females: MLN
CD45$^+$CD4$^+$TCRβ$^+$Foxp3$^-$

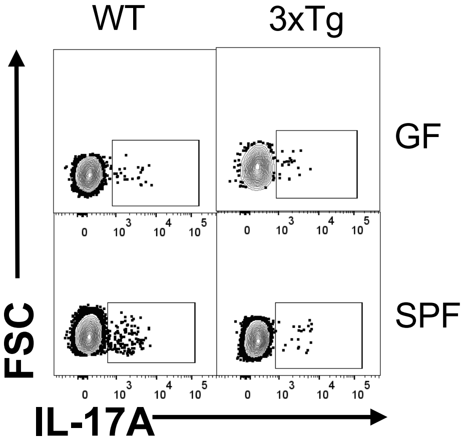

3xTg 12M Females: SC
CD45$^+$CD4$^+$TCRβ$^+$Foxp3$^-$

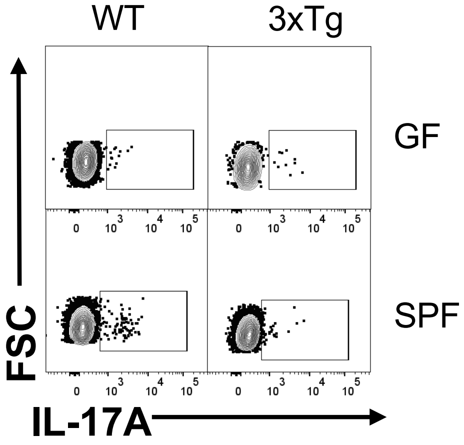

3xTg 12M Females: DC
CD45$^+$CD4$^+$TCRβ$^+$Foxp3$^-$

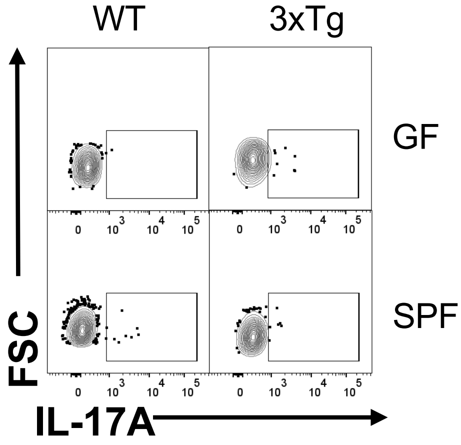

3xTg 12M Males: SP
CD45$^+$CD4$^+$TCRβ$^+$Foxp3$^-$

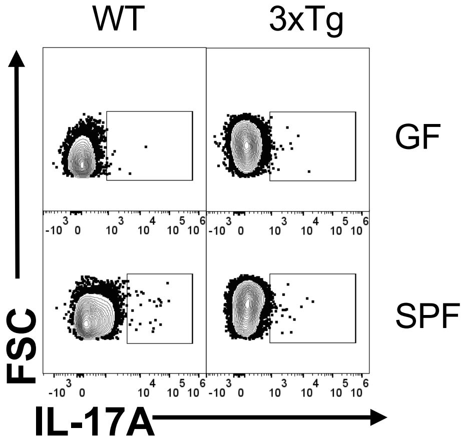

3xTg 12M Males: MLN
CD45$^+$CD4$^+$TCRβ$^+$Foxp3$^-$

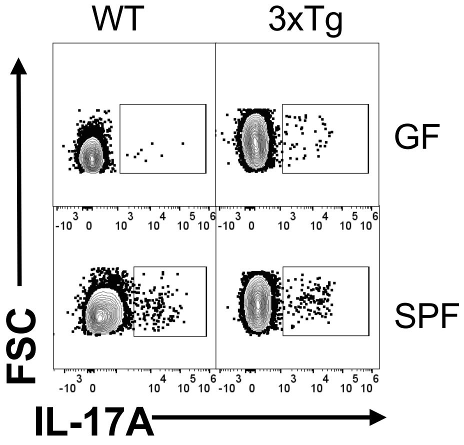

3xTg 7M Males: SC
CD45$^+$CD4$^+$TCRβ$^+$Foxp3$^-$

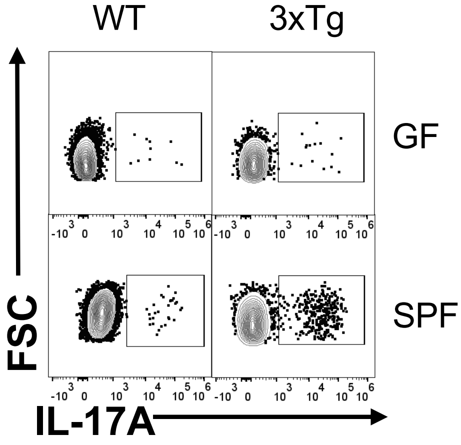

3xTg 7M Males: DC
CD45$^+$CD4$^+$TCRβ$^+$Foxp3$^-$

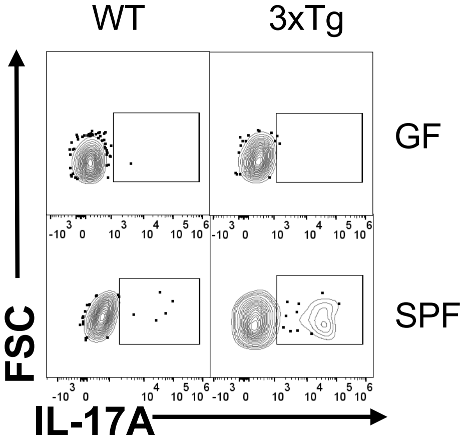

Representative Flow Cytometry Plots for
Fig. 4, S7: IL-17A$^+$ T cells and MFI [5xFAD Females]

5xFAD 5M Females: SI
CD45$^+$CD4$^+$TCRβ$^+$Foxp3$^-$

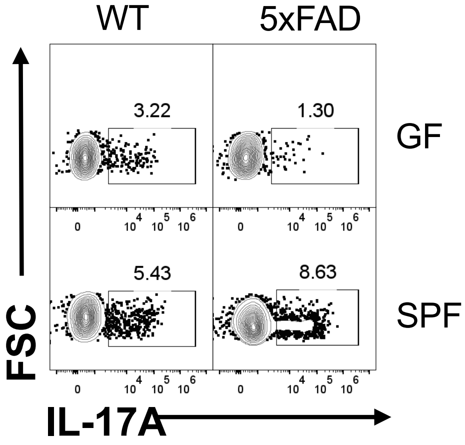

5xFAD 5M Females: LI
CD45$^+$CD4$^+$TCRβ$^+$Foxp3$^-$

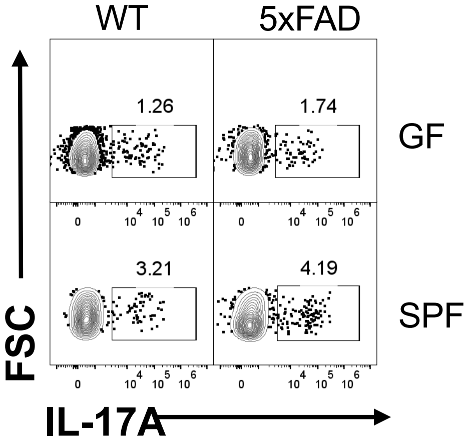

5xFAD 5M Females: SP
CD45$^+$CD4$^+$TCRβ$^+$Foxp3$^-$

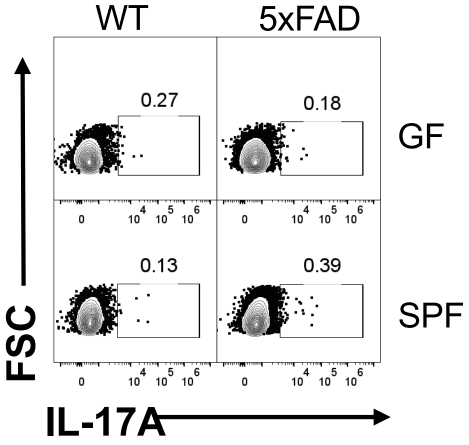

5xFAD 5M Females: MLN
CD45$^+$CD4$^+$TCRβ$^+$Foxp3$^-$

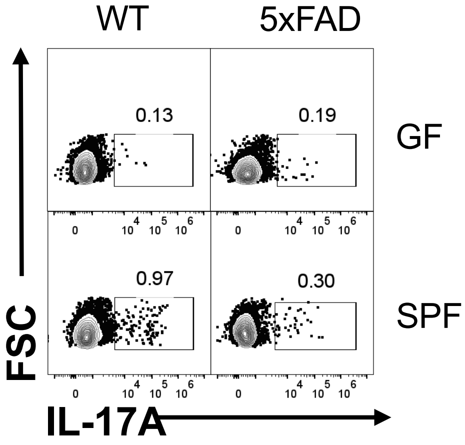

5xFAD 5M Females: SC
CD45$^+$CD4$^+$TCRβ$^+$Foxp3$^-$

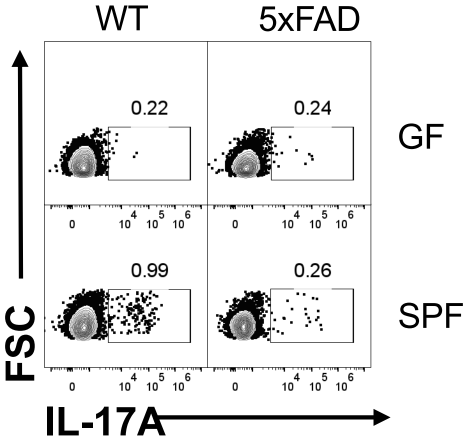

Representative Flow Cytometry Plots for Fig. 4, S7: IL-17A$^+$ T cells and MFI [5xFAD Males]

5xFAD 5M Males: SI
CD45$^+$CD4$^+$TCRβ$^+$Foxp3$^-$

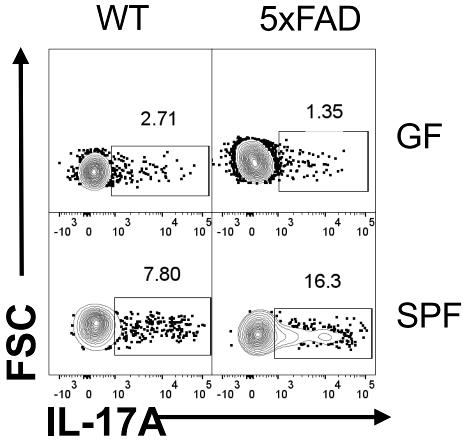

5xFAD 5M Males: LI
CD45$^+$CD4$^+$TCRβ$^+$Foxp3$^-$

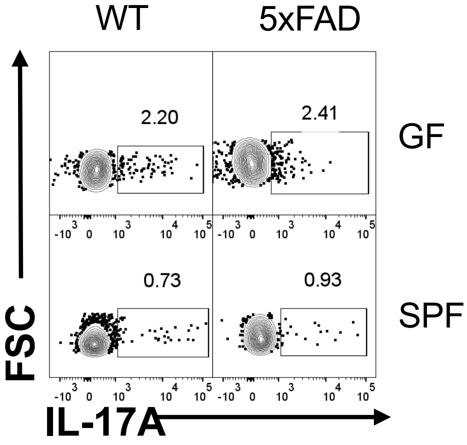

5xFAD 5M Males: SP
CD45$^+$CD4$^+$TCRβ$^+$Foxp3$^-$

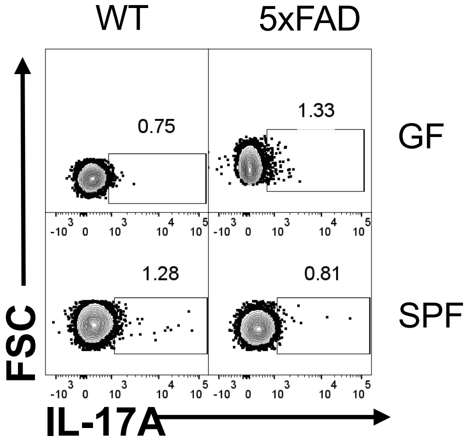

5xFAD 5M Males: MLN
CD45$^+$CD4$^+$TCRβ$^+$Foxp3$^-$

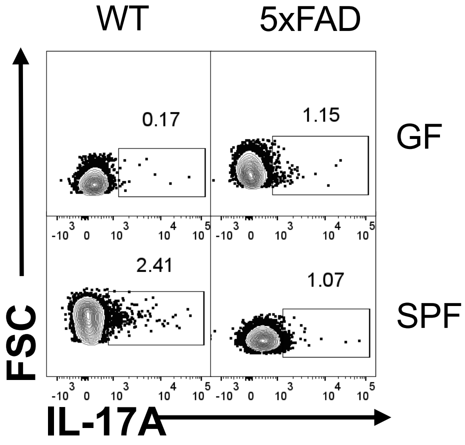

5xFAD 5M Males: SC
CD45$^+$CD4$^+$TCRβ$^+$Foxp3$^-$

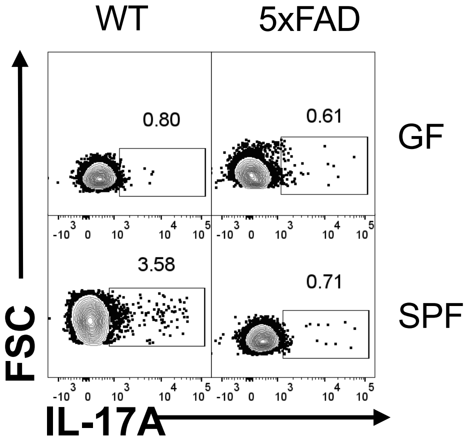

Representative Flow Cytometry Plots for Fig. 5, S5, S12: CD4$^+$ and CD8$^+$ T cells [3xTg SPF Females]

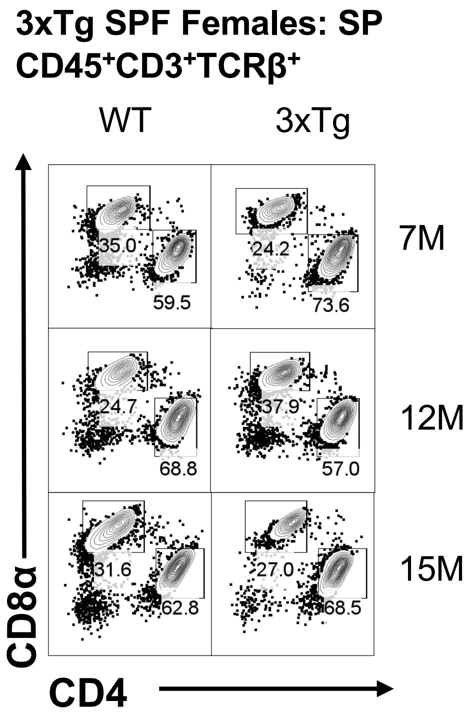

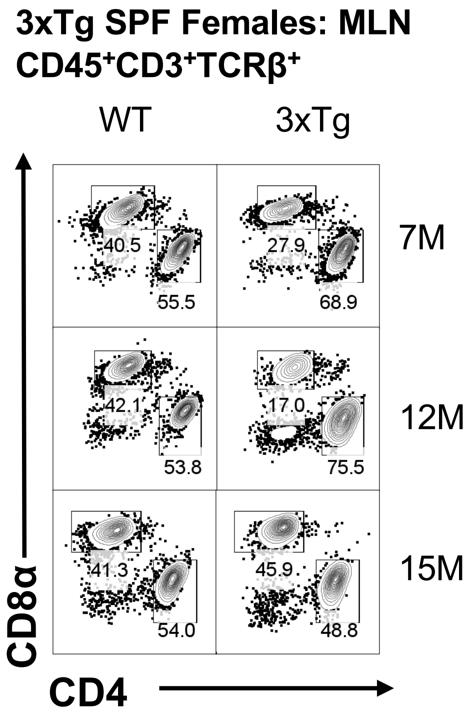

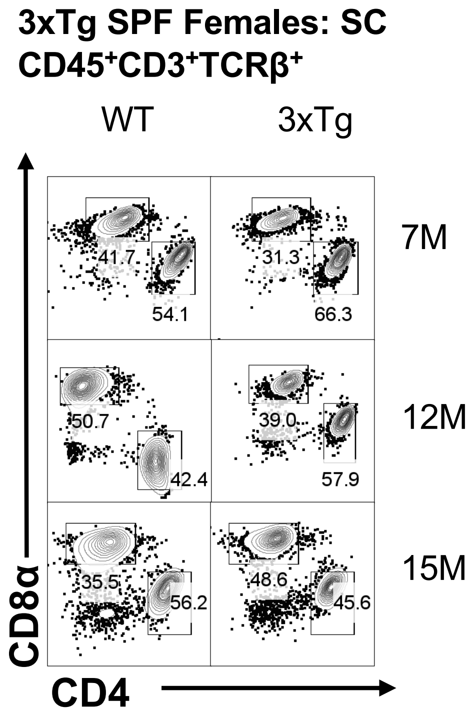

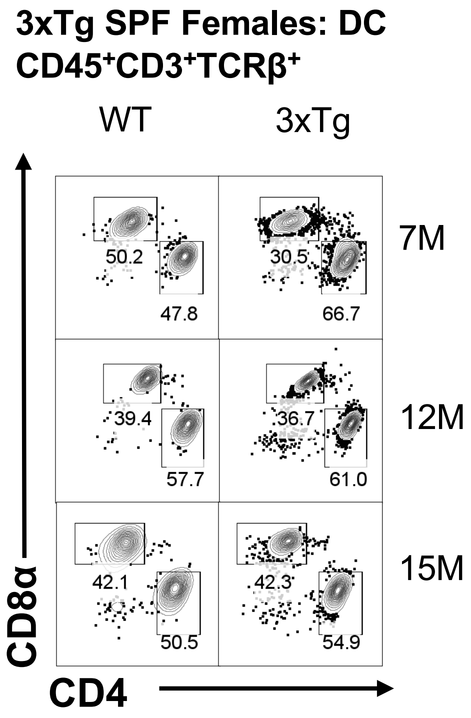

Representative Flow Cytometry Plots for Fig. 5, S13, S14: T and B cells [3xTg SPF Females]

3xTg SPF Females: SP
CD45⁺

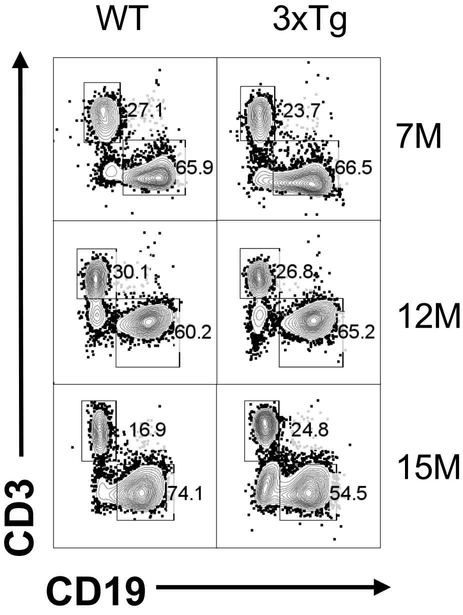

3xTg SPF Females: MLN
CD45⁺

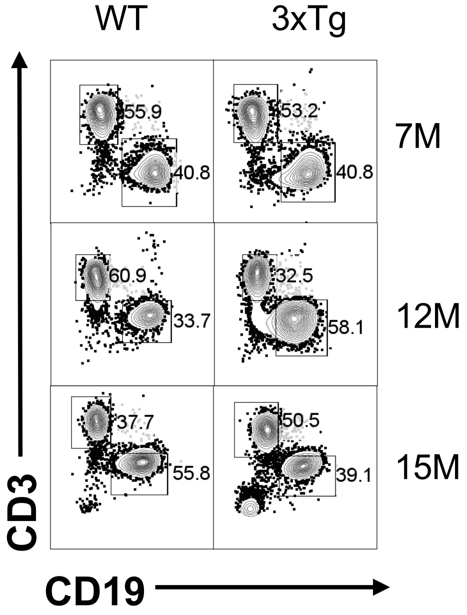

3xTg SPF Females: SC
CD45⁺

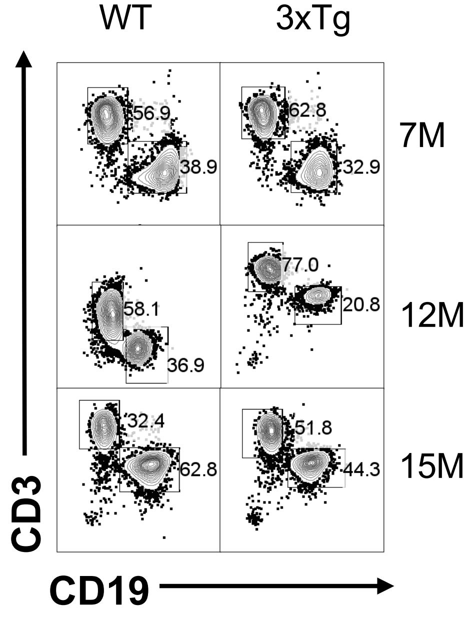

3xTg SPF Females: DC
CD45⁺

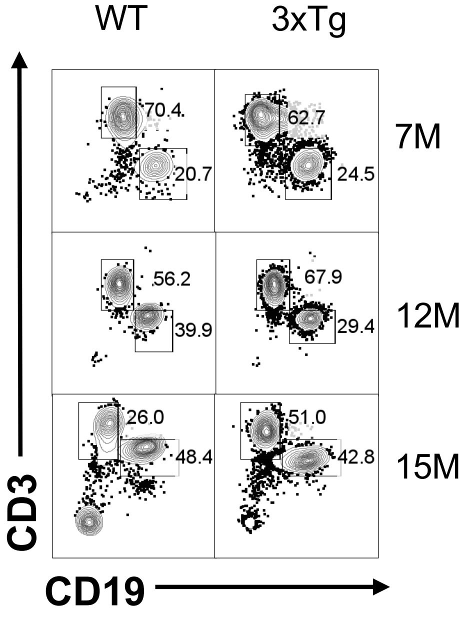

Representative Flow Cytometry Plots for Fig. 5: IL-17A+ T cells and MFI [5xFAD Females]

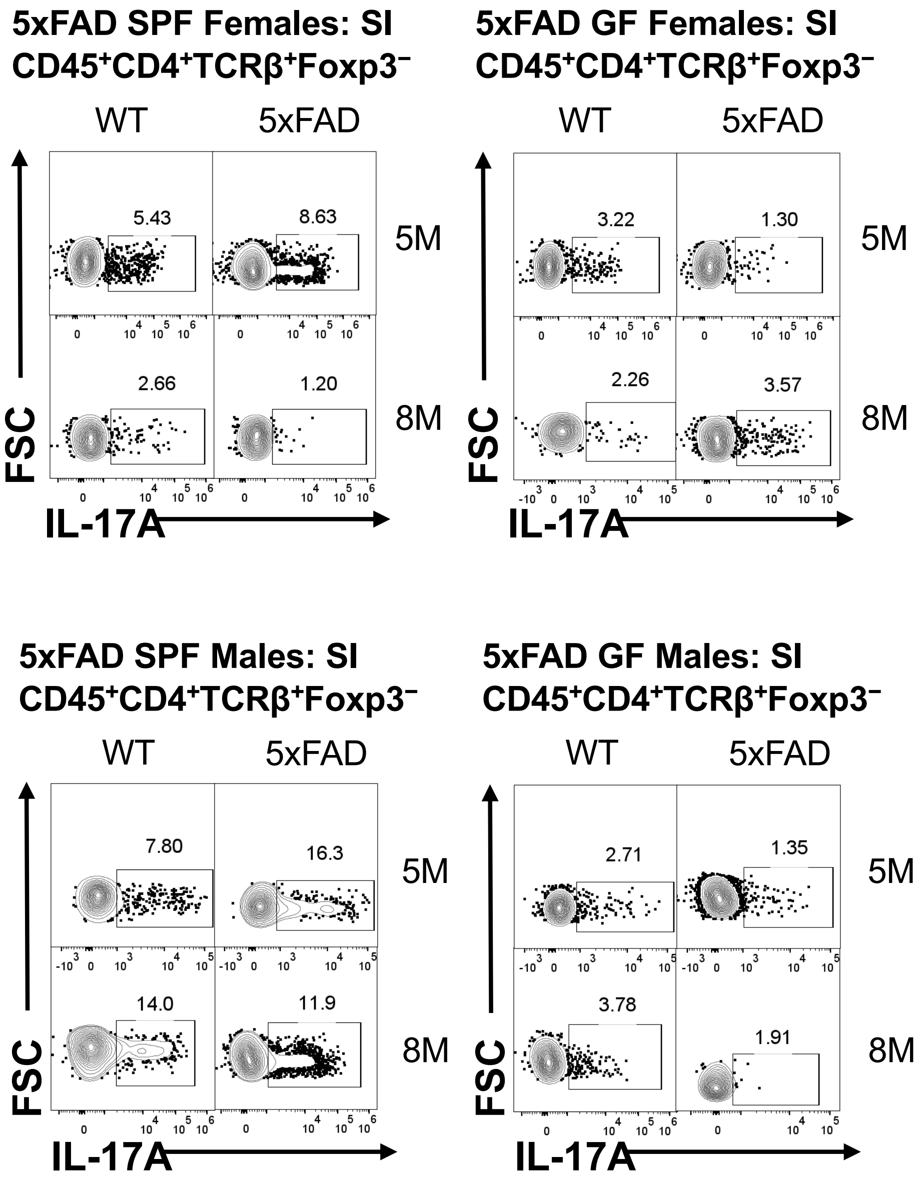

Representative Flow Cytometry Plots for Fig. S3 and S14: T and B cells [5xFAD 5M]

5xFAD 5M Females: SP
CD45+

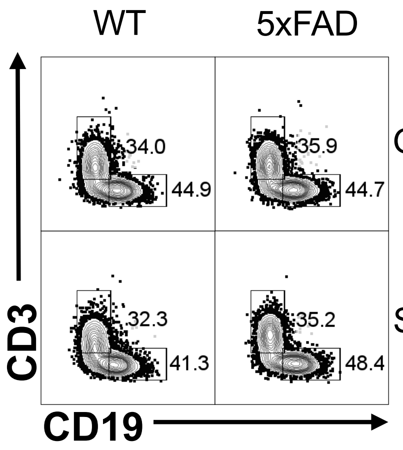

5xFAD 5M Females: MLN
CD45+

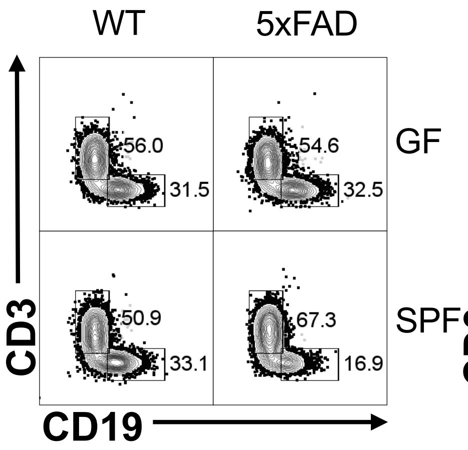

5xFAD 5M Females: SC
CD45+

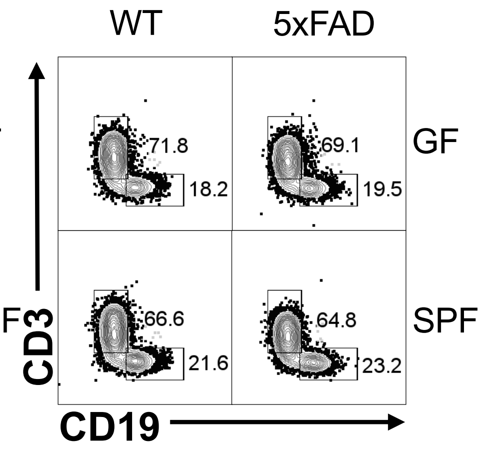

5xFAD 5M Females: DC
CD45+

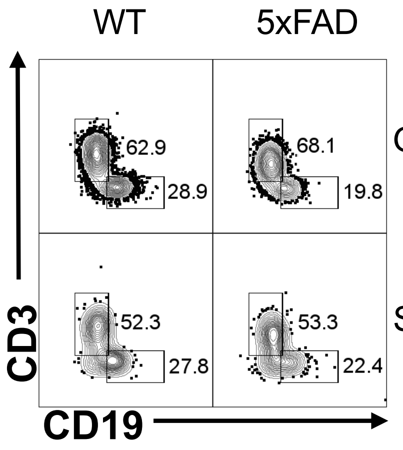

5xFAD 5M Males: SP
CD45+

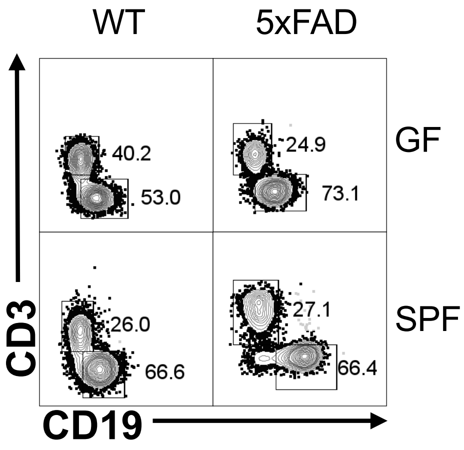

5xFAD 5M Males: MLN
CD45+

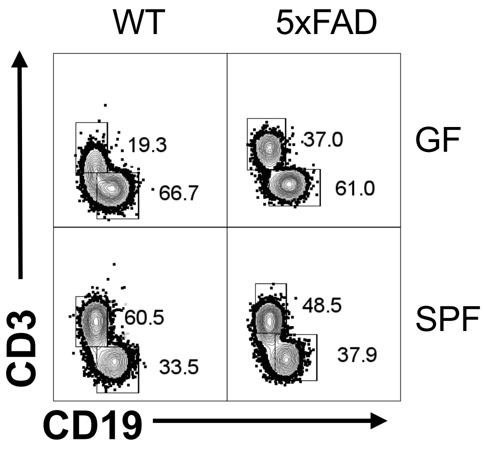

5xFAD 5M Males: SC
CD45+

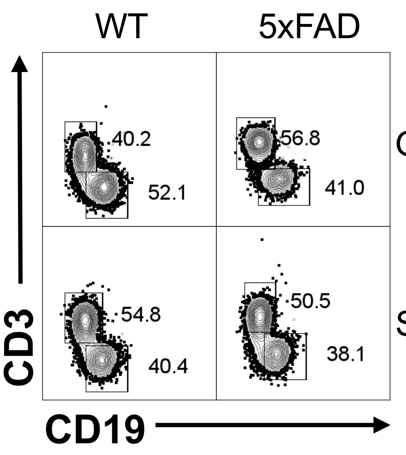

5xFAD 5M Males: DC
CD45+

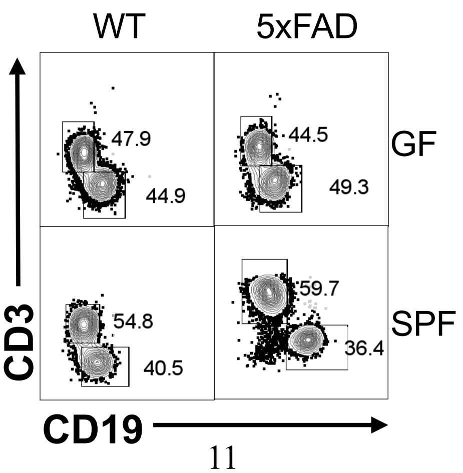

Representative Flow Cytometry Plots for Fig. S3: T and B cells [5xFAD 5M]

5xFAD 5M Females: SI
CD45+

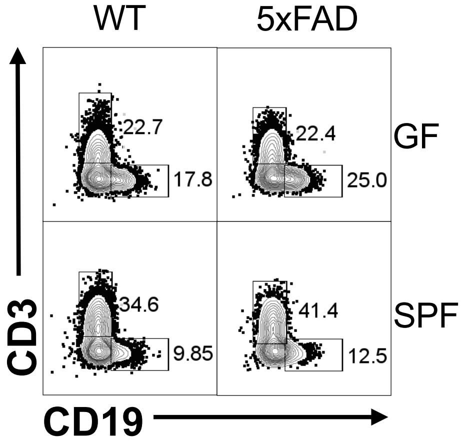

5xFAD 5M Females: LI
CD45+

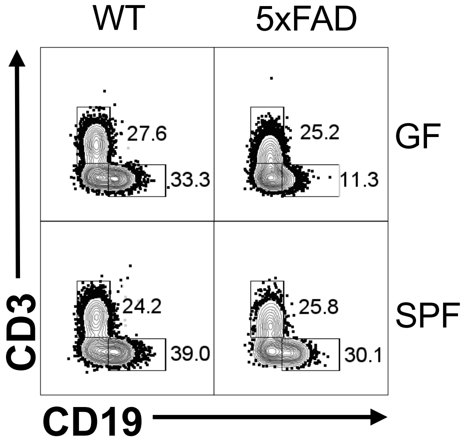

5xFAD 5M Males: SI
CD45+

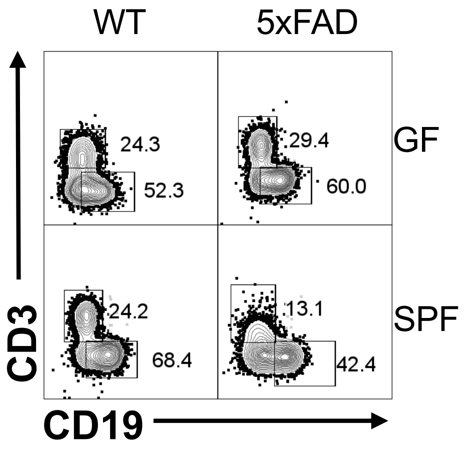

5xFAD 5M Males: LI
CD45+

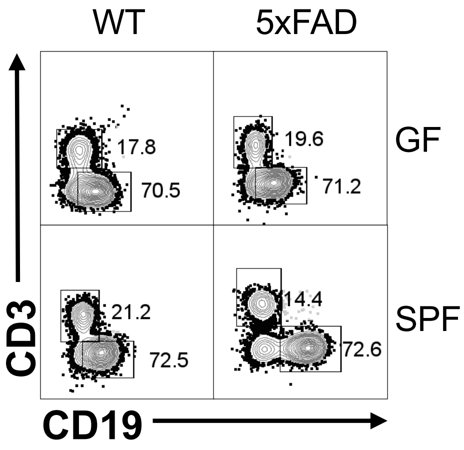

Representative Flow Cytometry Plots for Fig. S4, S12: $CD4^+$ and $CD8^+$ T cells [3xTg SPF Males]

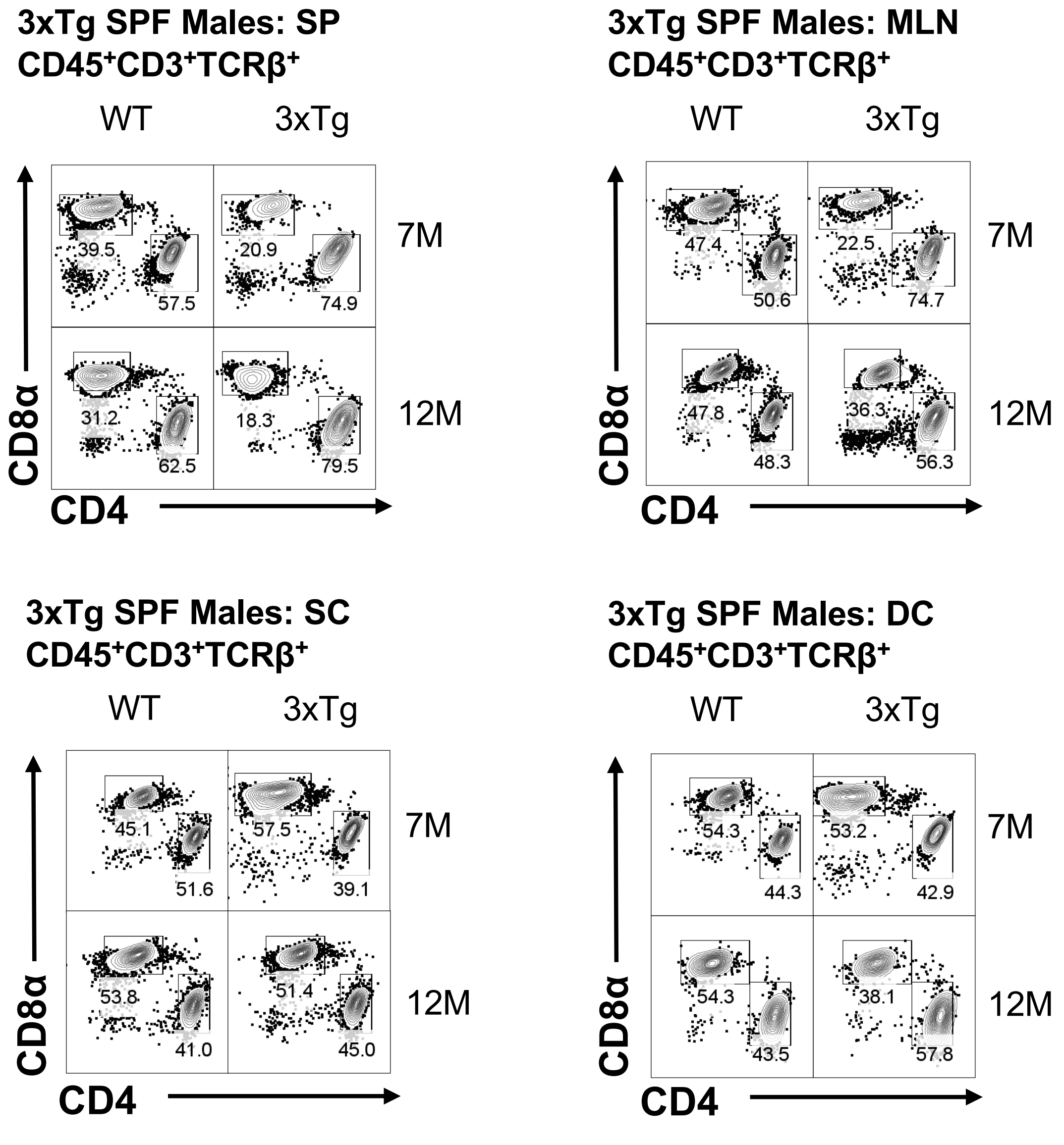

Representative Flow Cytometry Plots for Fig. S4, S12: CD4$^+$ and CD8$^+$ T cells [3xTg GF Females]

3xTg GF Females: SP
CD45$^+$CD3$^+$TCRβ$^+$

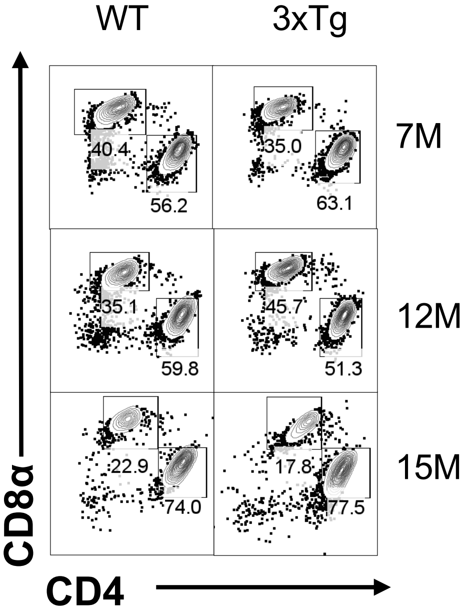

3xTg GF Females: MLN
CD45$^+$CD3$^+$TCRβ$^+$

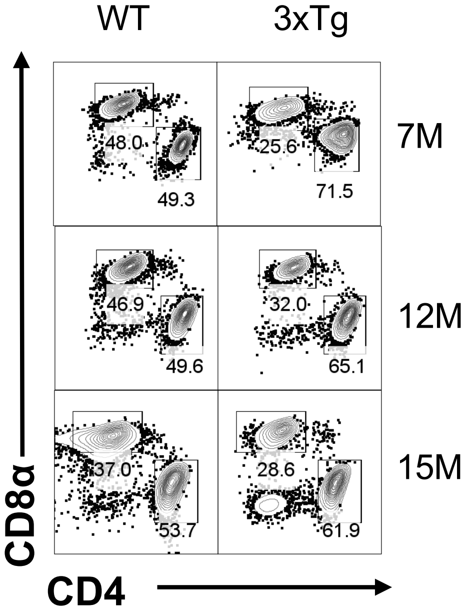

3xTg GF Females: SC
CD45$^+$CD3$^+$TCRβ$^+$

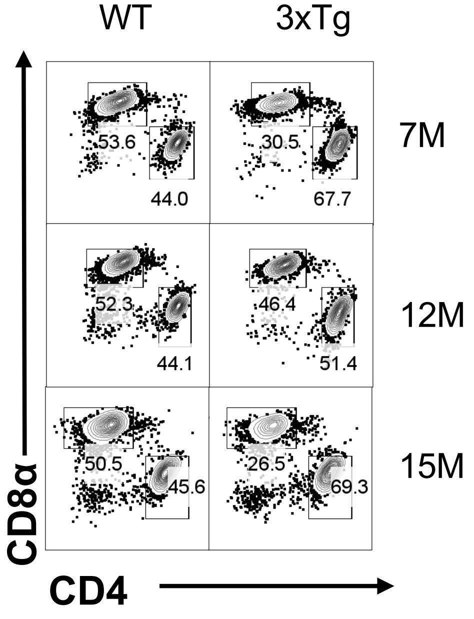

3xTg GF Females: DC
CD45$^+$CD3$^+$TCRβ$^+$

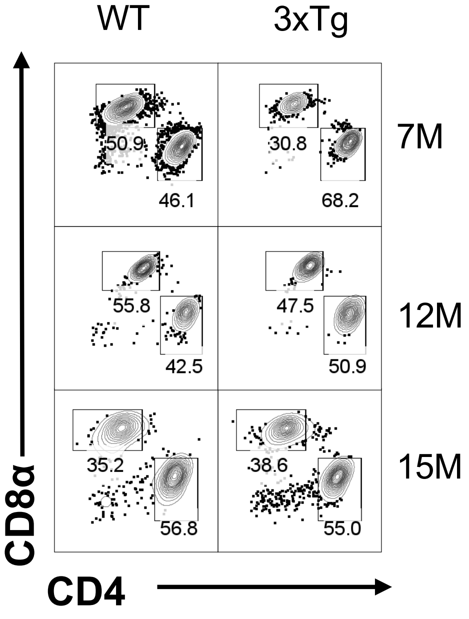

Representative Flow Cytometry Plots for
Fig. S4, S12: CD4$^+$ and CD8$^+$ T cells [3xTg GF Males]

3xTg GF Males: SP
CD45$^+$CD3$^+$TCRβ$^+$

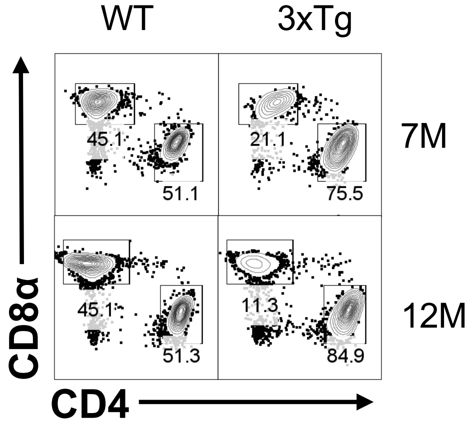

3xTg GF Males: MLN
CD45$^+$CD3$^+$TCRβ$^+$

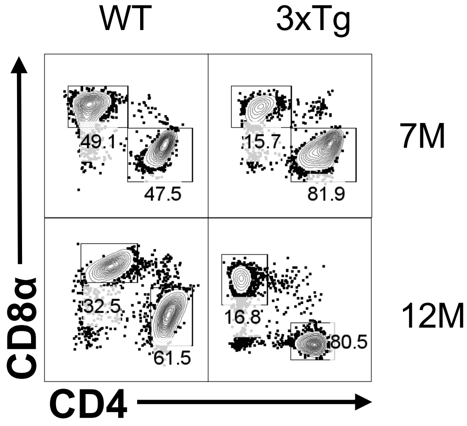

3xTg GF Males: SC
CD45$^+$CD3$^+$TCRβ$^+$

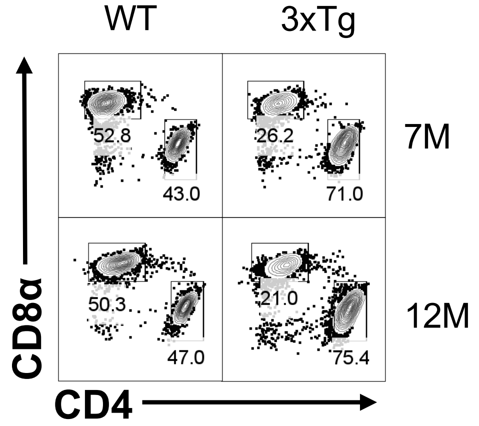

3xTg GF Males: DC
CD45$^+$CD3$^+$TCRβ$^+$

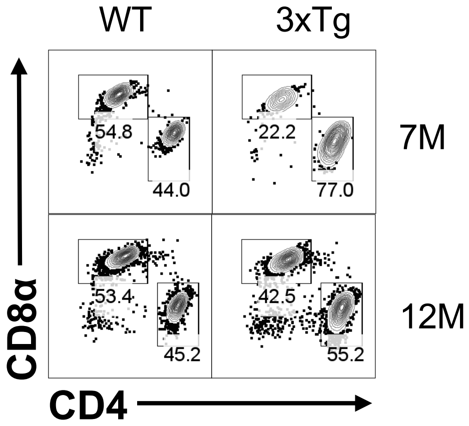

Representative Flow Cytometry Plots for Fig. S5: IFNγ [3xTg]

3xTg 12M Females: SP
$CD45^+CD4^+TCR\beta^+Foxp3^-$

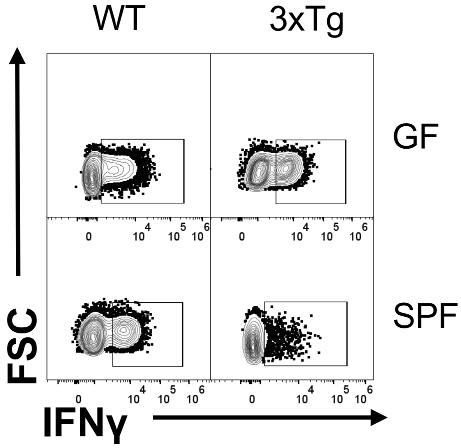

3xTg 12M Females: MLN
$CD45^+CD4^+TCR\beta^+Foxp3^-$

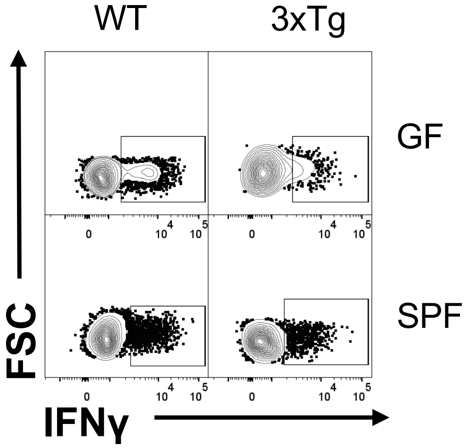

3xTg 12M Females: SC
$CD45^+CD4^+TCR\beta^+Foxp3^-$

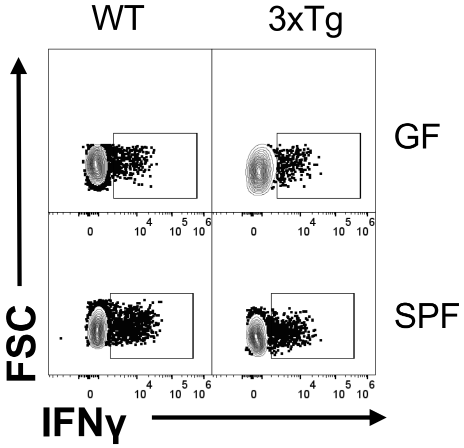

3xTg 12M Females: DC
$CD45^+CD4^+TCR\beta^+Foxp3^-$

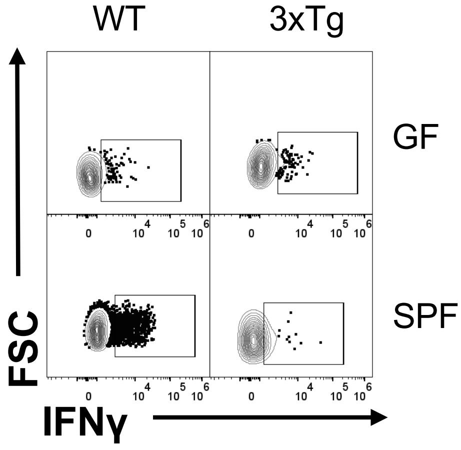

3xTg 12M Males: SP
$CD45^+CD4^+TCR\beta^+Foxp3^-$

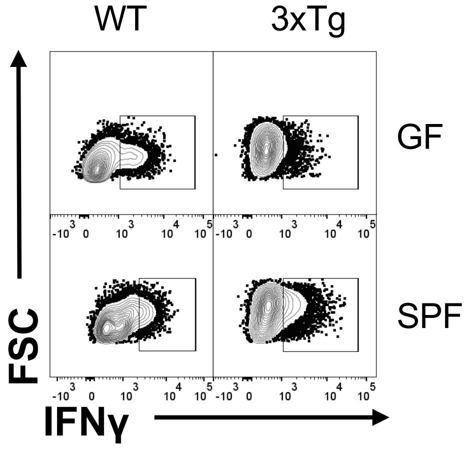

3xTg 12M Males: MLN
$CD45^+CD4^+TCR\beta^+Foxp3^-$

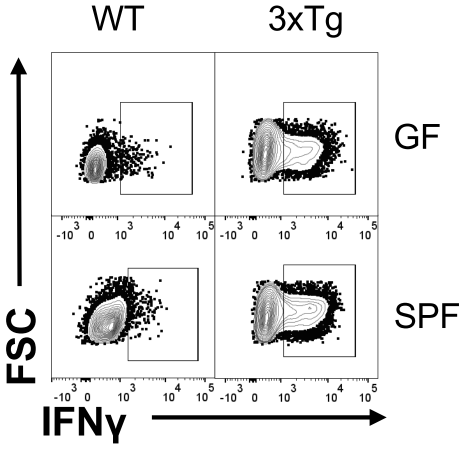

3xTg 12M Males: SC
$CD45^+CD4^+TCR\beta^+Foxp3^-$

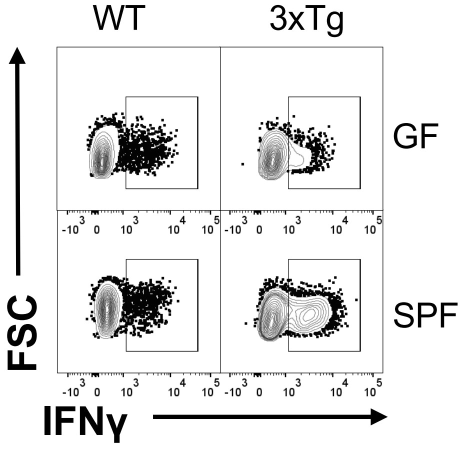

3xTg 12M Males: DC
$CD45^+CD4^+TCR\beta^+Foxp3^-$

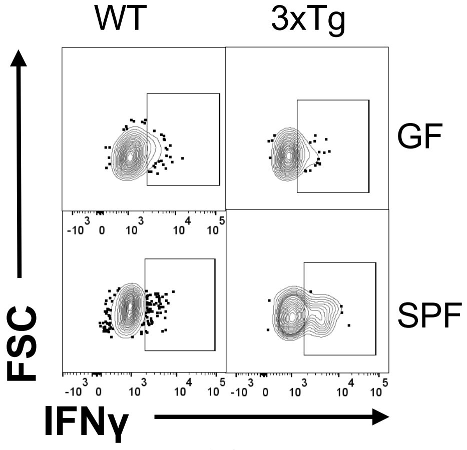

Representative Flow Cytometry Plots for Fig. S6: Foxp3$^+$ T cells [3xTg]

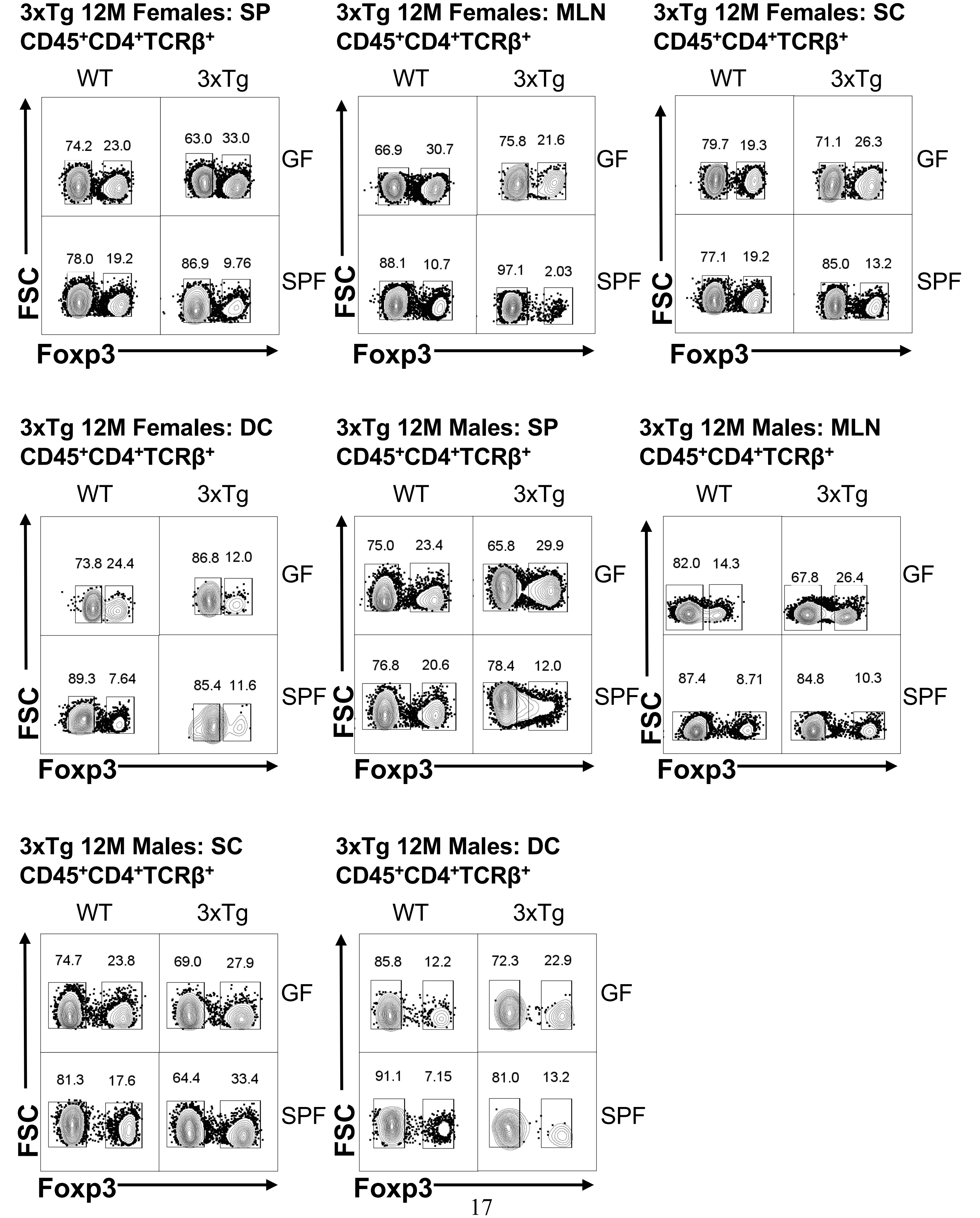

Representative Flow Cytometry Plots for Fig. S8, S9: IFNγ$^+$/GM-CSF$^+$ T cells [5xFAD Females]

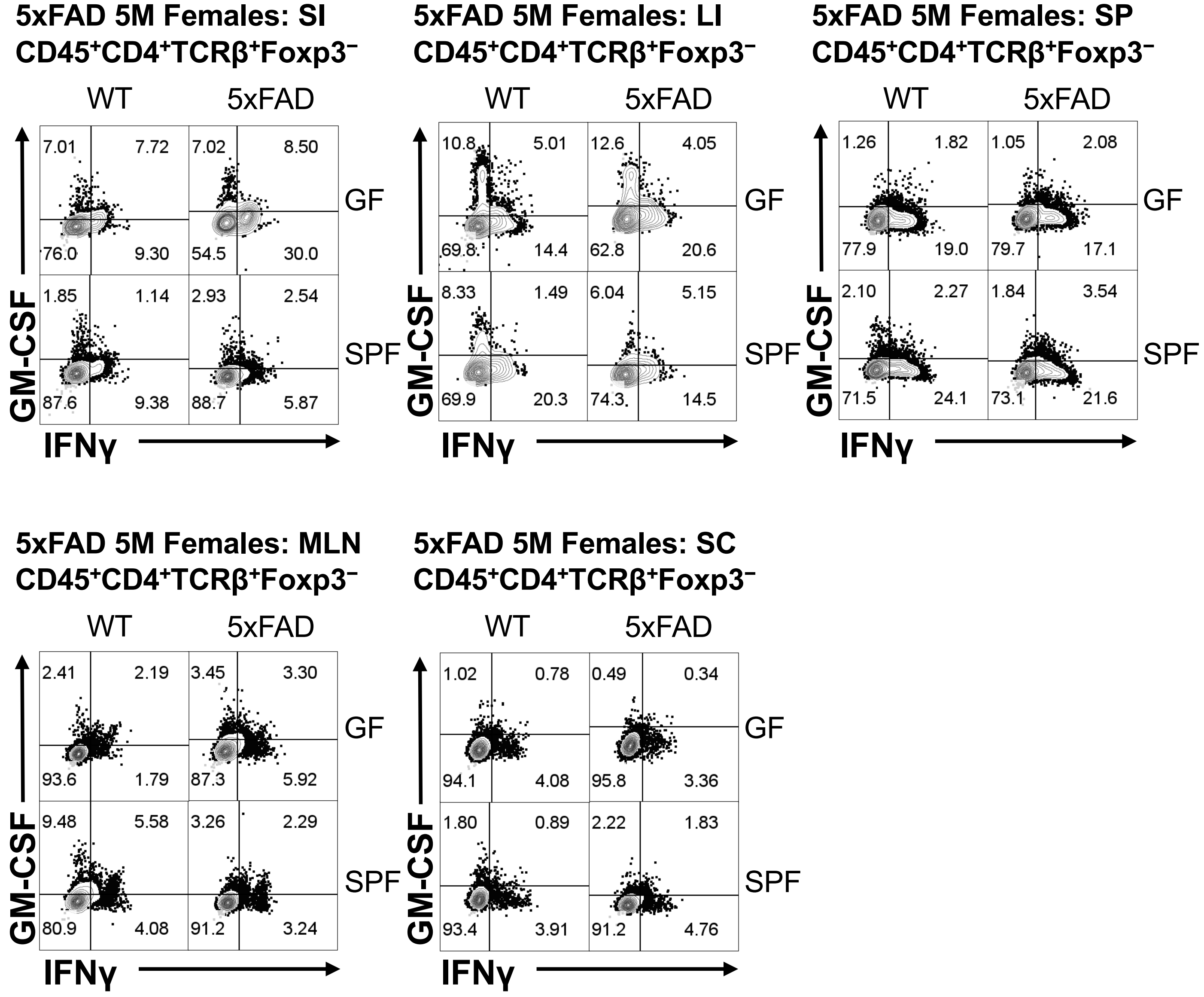

Representative Flow Cytometry Plots for Fig. S8, S9: IFNγ$^+$/GM-CSF$^+$ T cells [5xFAD Males]

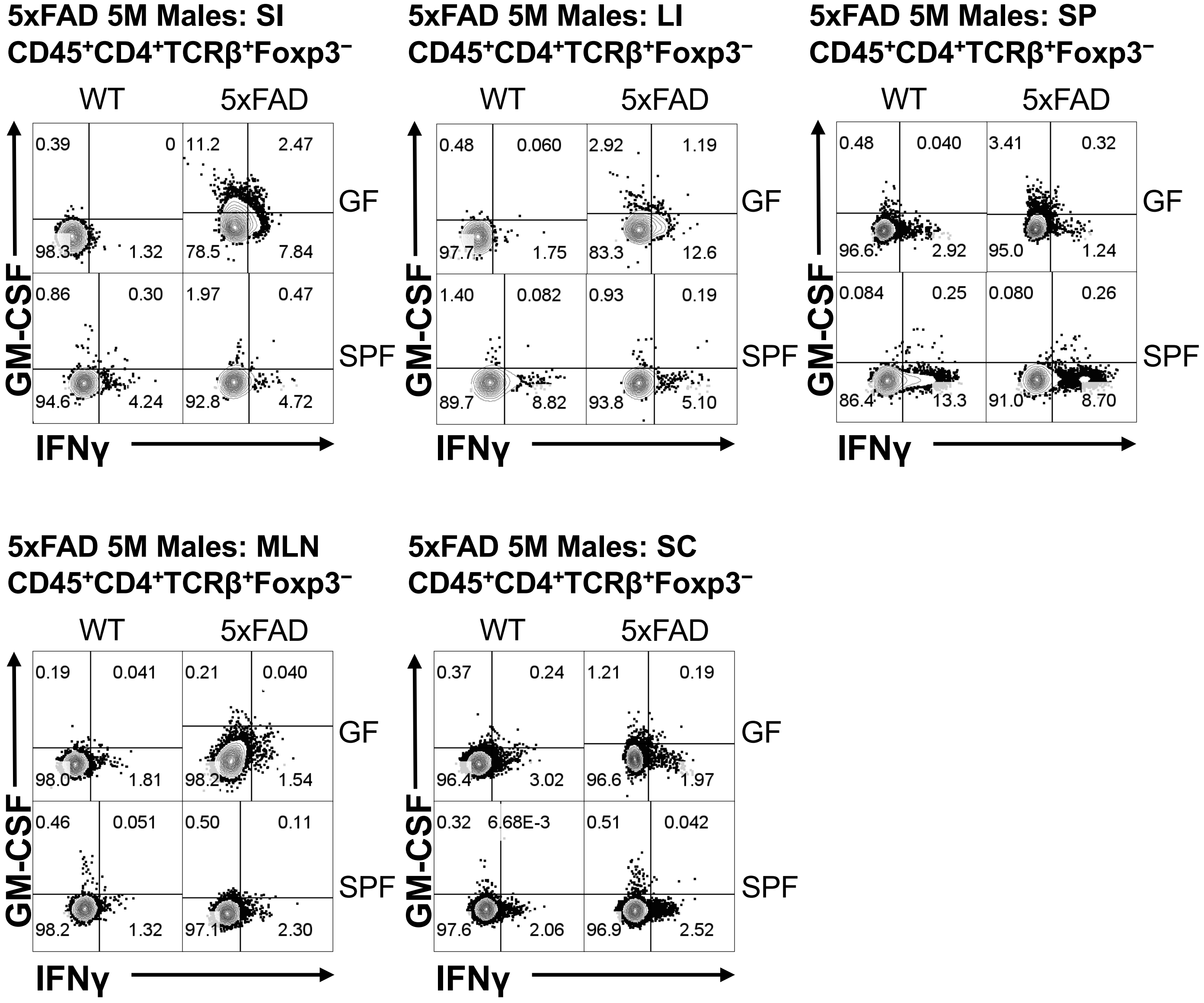

Representative Flow Cytometry Plots for Fig. S8: IFNγ$^+$ MFI [5xFAD Females]

5xFAD 5M Females: SI
CD45$^+$CD4$^+$TCRβ$^+$Foxp3$^-$

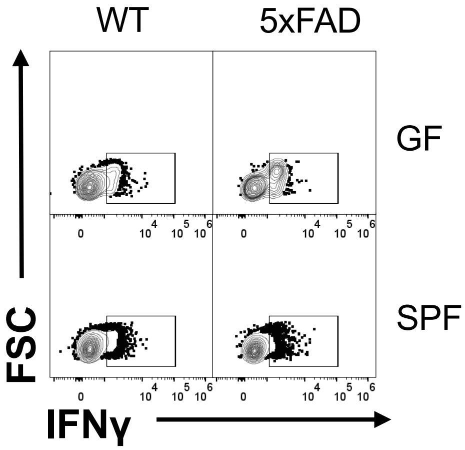

5xFAD 5M Females: LI
CD45$^+$CD4$^+$TCRβ$^+$Foxp3$^-$

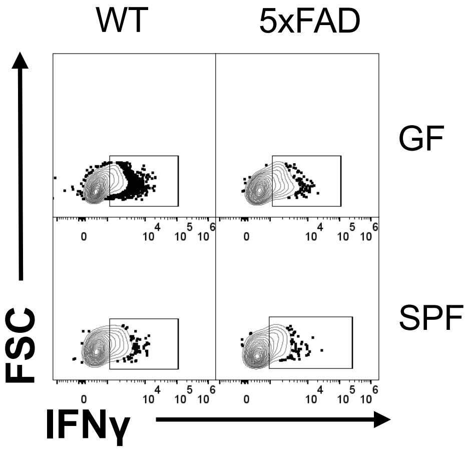

5xFAD 5M Females: SP
CD45$^+$CD4$^+$TCRβ$^+$Foxp3$^-$

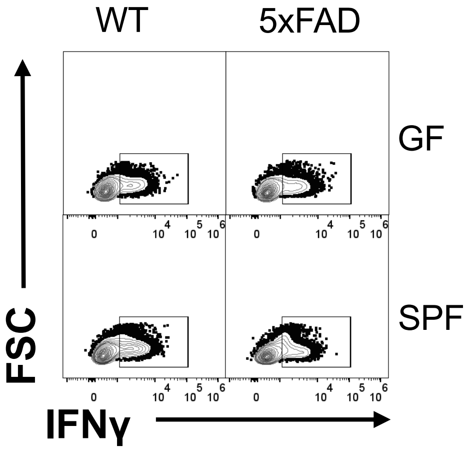

5xFAD 5M Females: MLN
CD45$^+$CD4$^+$TCRβ$^+$Foxp3$^-$

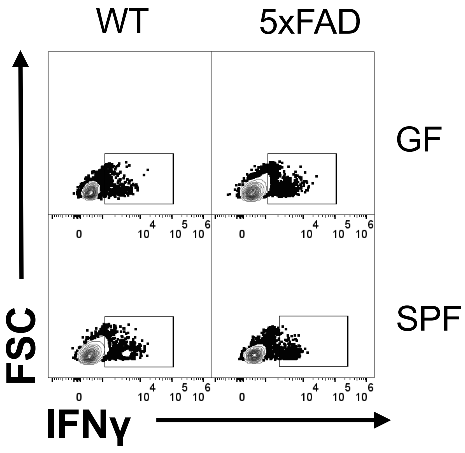

5xFAD 5M Females: SC
CD45$^+$CD4$^+$TCRβ$^+$Foxp3$^-$

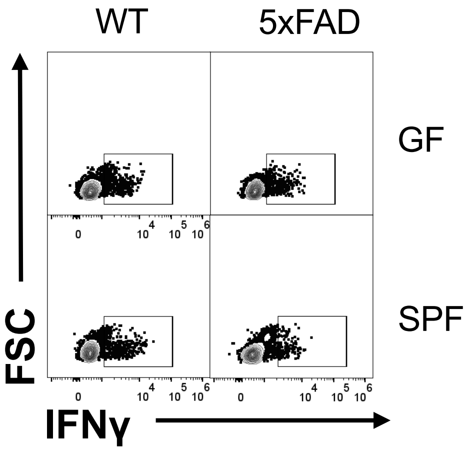

Representative Flow Cytometry Plots for Fig. S8: IFNγ^{+} MFI [5xFAD Males]

5xFAD 5M Males: SI
CD45^{+}CD4^{+}TCRβ^{+}Foxp3^{-}

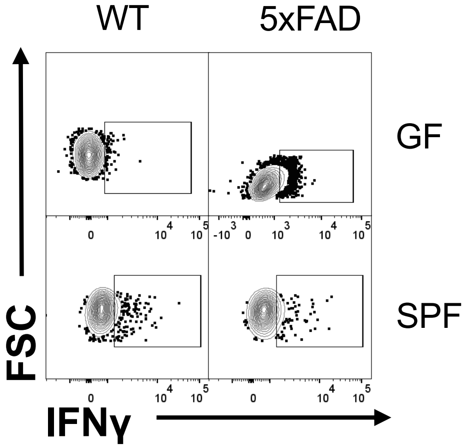

5xFAD 5M Males: LI
CD45^{+}CD4^{+}TCRβ^{+}Foxp3^{-}

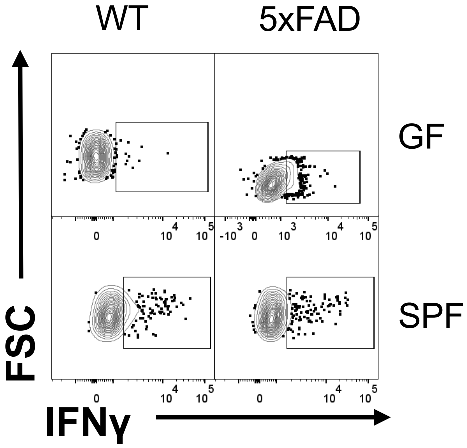

5xFAD 5M Males: SP
CD45^{+}CD4^{+}TCRβ^{+}Foxp3^{-}

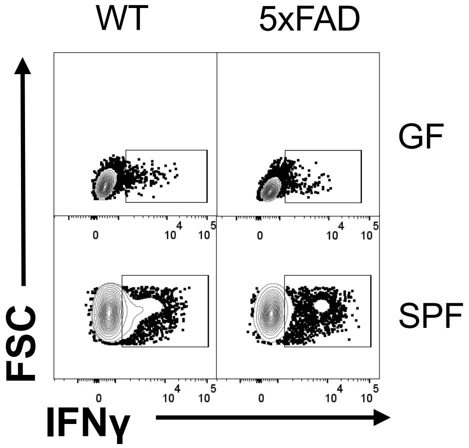

5xFAD 5M Males: MLN
CD45^{+}CD4^{+}TCRβ^{+}Foxp3^{-}

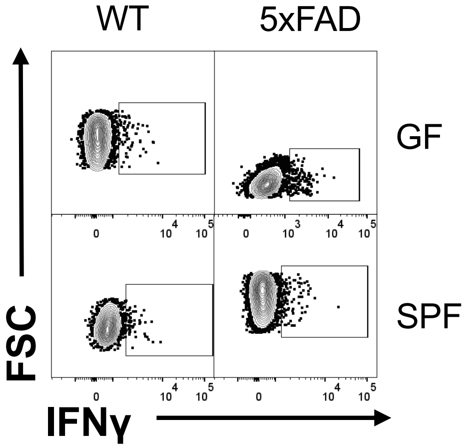

5xFAD 5M Males: SC
CD45^{+}CD4^{+}TCRβ^{+}Foxp3^{-}

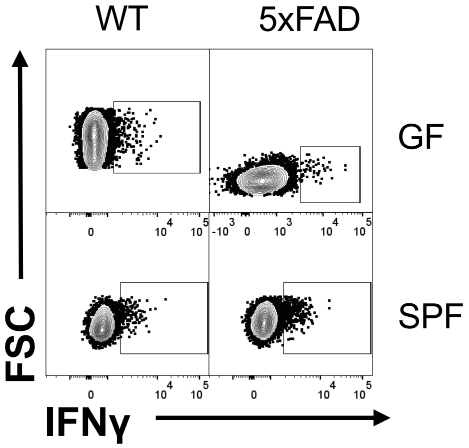

Representative Flow Cytometry Plots for Fig. S9: GM-CSF$^+$ MFI [5xFAD Females]

5xFAD 5M Females: SI
CD45$^+$CD4$^+$TCRβ$^+$Foxp3$^-$

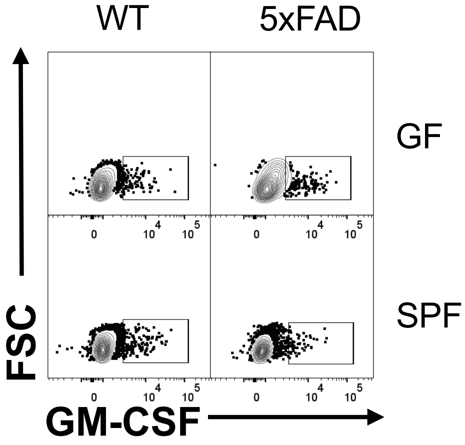

5xFAD 5M Females: LI
CD45$^+$CD4$^+$TCRβ$^+$Foxp3$^-$

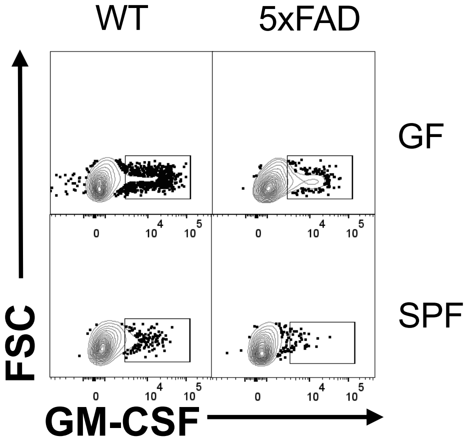

5xFAD 5M Females: SP
CD45$^+$CD4$^+$TCRβ$^+$Foxp3$^-$

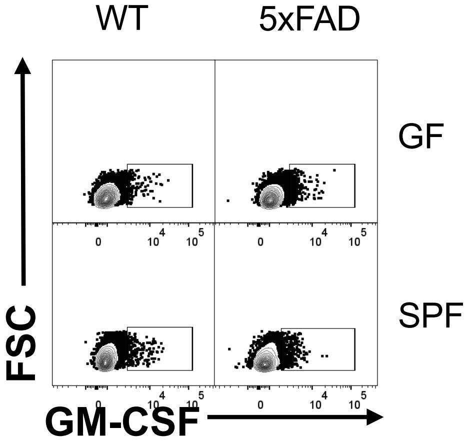

5xFAD 5M Females: MLN
CD45$^+$CD4$^+$TCRβ$^+$Foxp3$^-$

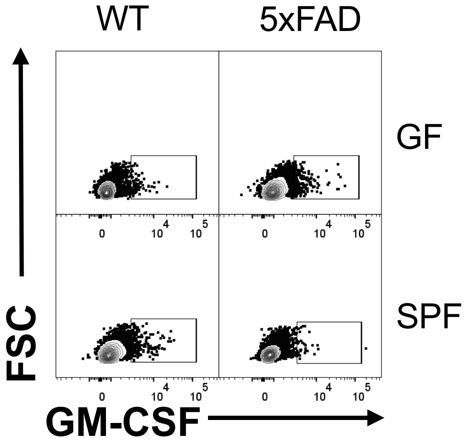

5xFAD 5M Females: SC
CD45$^+$CD4$^+$TCRβ$^+$Foxp3$^-$

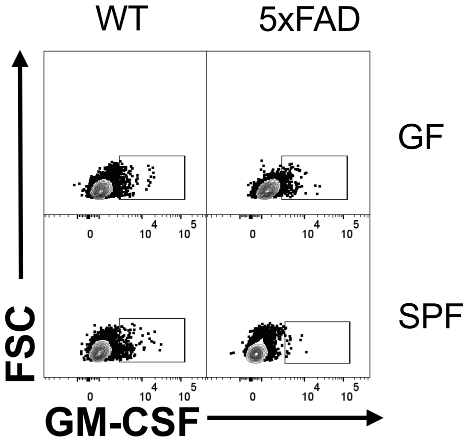

Representative Flow Cytometry Plots for Fig. S9: GM-CSF^{+} MFI [5xFAD Males]

5xFAD 5M Males: SI
CD45^{+}CD4^{+}TCRβ^{+}Foxp3^{-}

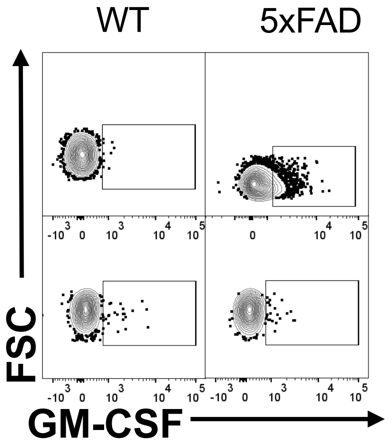

5xFAD 5M Males: LI
CD45^{+}CD4^{+}TCRβ^{+}Foxp3^{-}

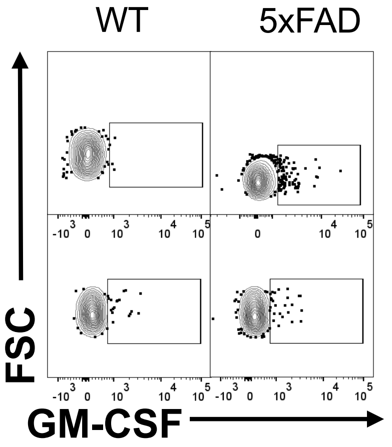

5xFAD 5M Males: SP
CD45^{+}CD4^{+}TCRβ^{+}Foxp3^{-}

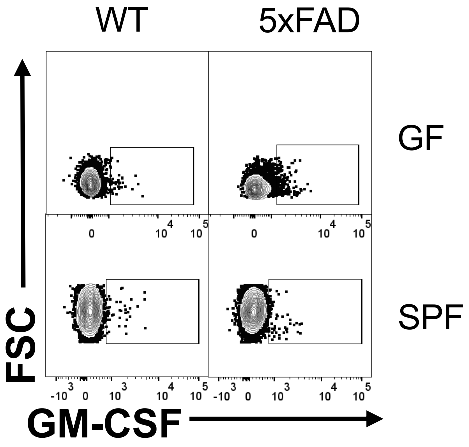

5xFAD 5M Males: MLN
CD45^{+}CD4^{+}TCRβ^{+}Foxp3^{-}

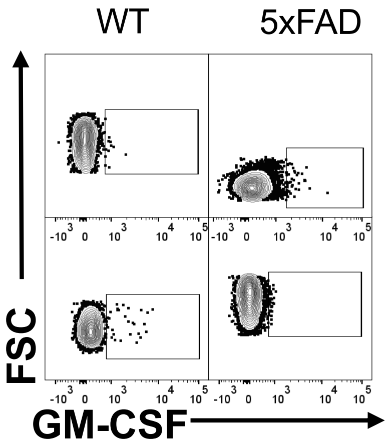

5xFAD 5M Males: SC
CD45^{+}CD4^{+}TCRβ^{+}Foxp3^{-}

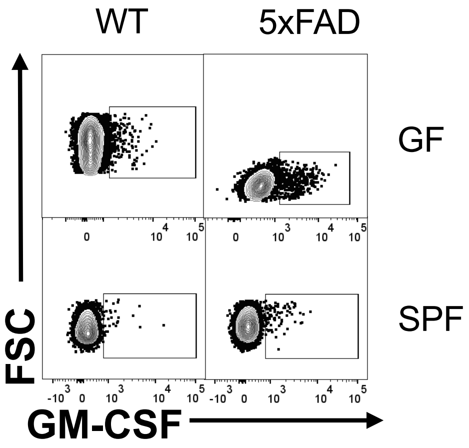

Representative Flow Cytometry Plots for Fig. S10: IFNγ⁺/IL-4⁺ T cells [5xFAD Females]

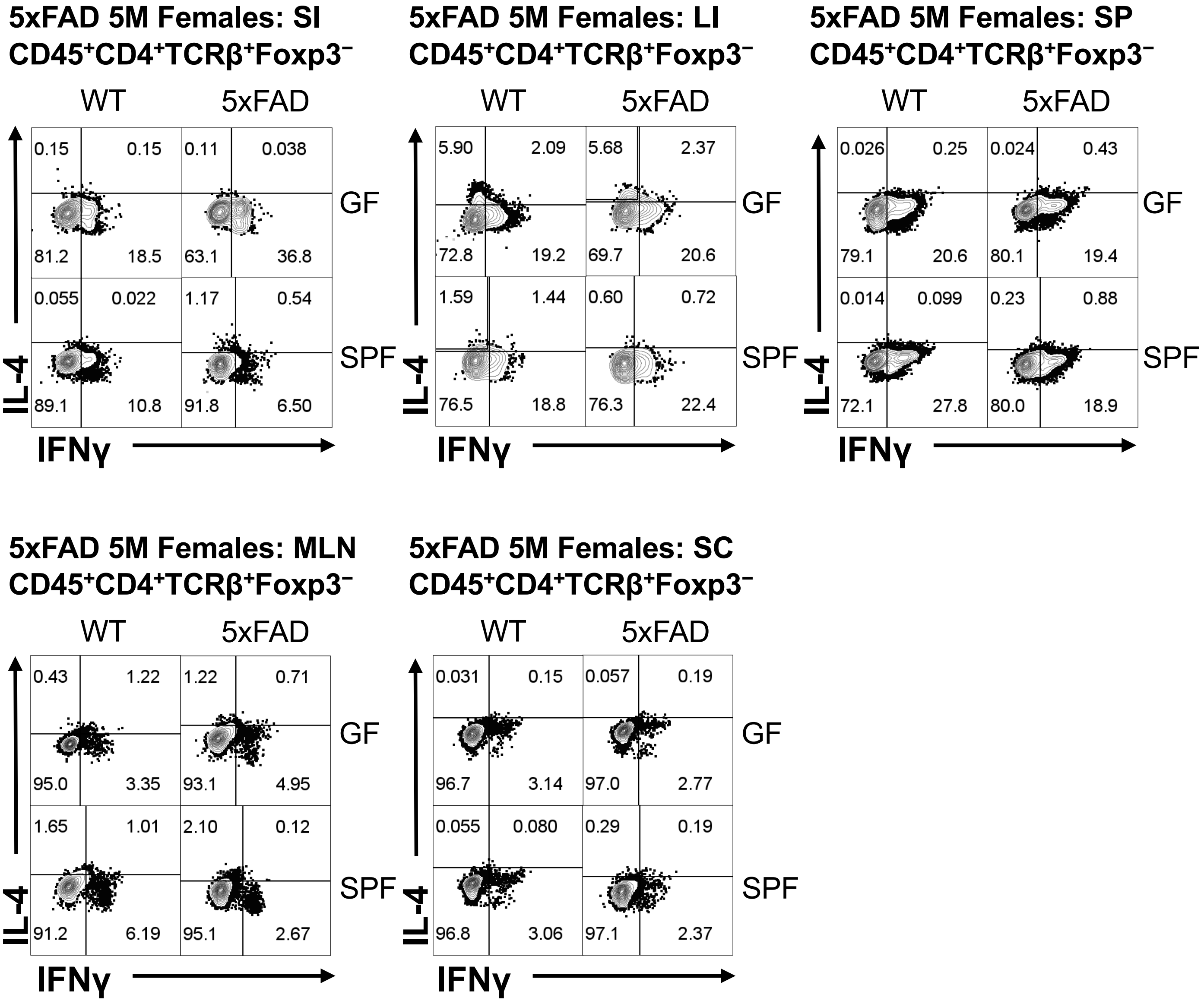

Representative Flow Cytometry Plots for Fig. S10: IFNγ⁺/IL-4⁺ T cells [5xFAD Males]

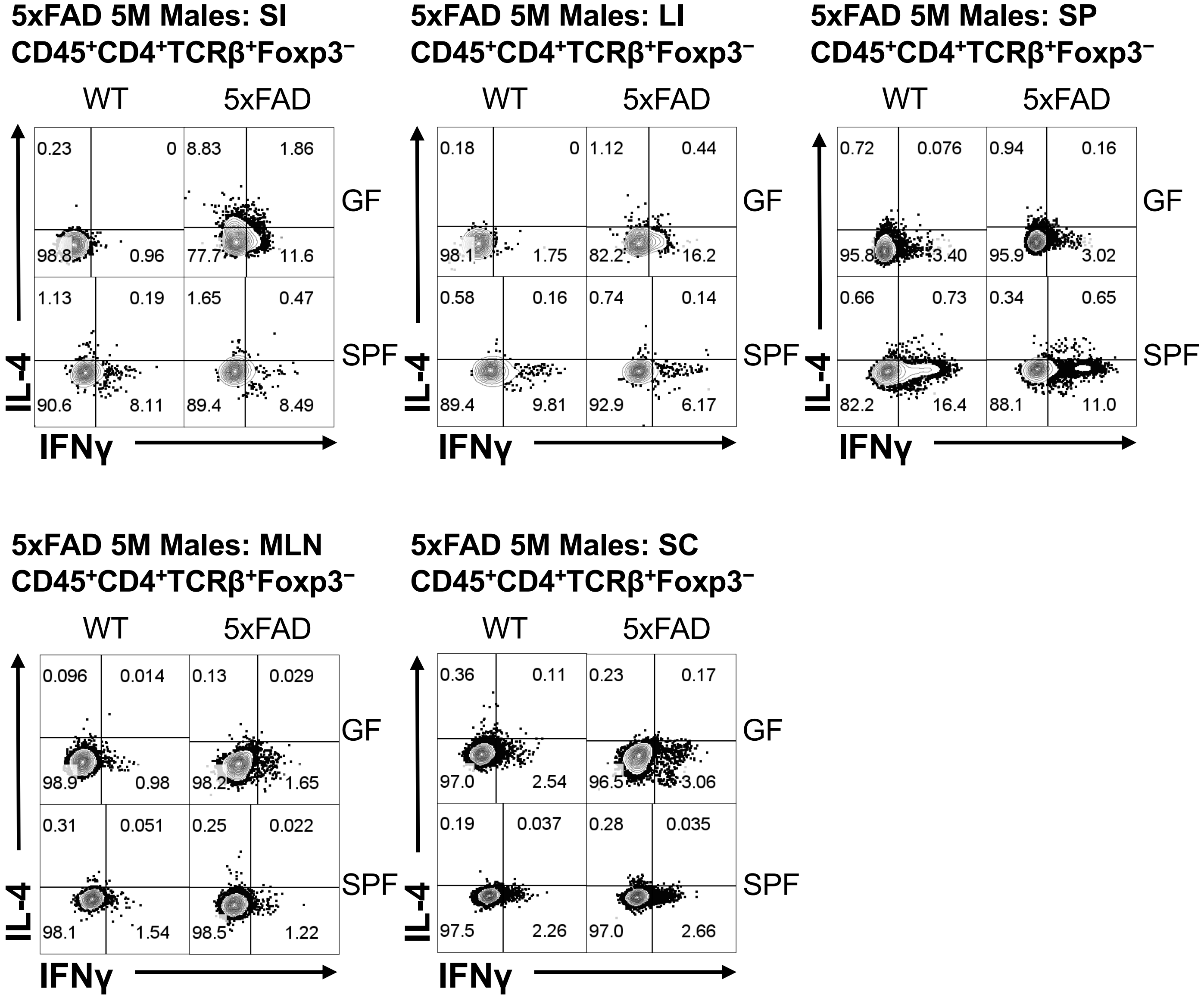

Representative Flow Cytometry Plots for Fig. S10: IL-4^{+} MFI [5xFAD Females]

5xFAD 5M Females: SI
CD45^{+}CD4^{+}TCRβ^{+}Foxp3^{-}

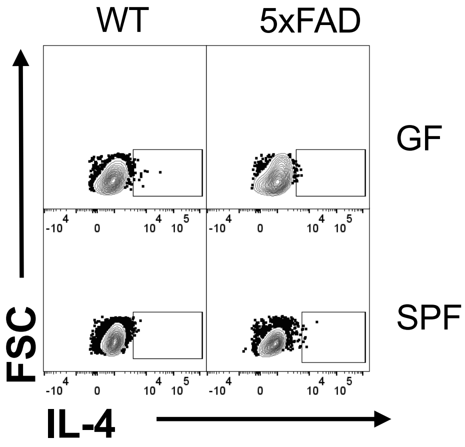

5xFAD 5M Females: LI
CD45^{+}CD4^{+}TCRβ^{+}Foxp3^{-}

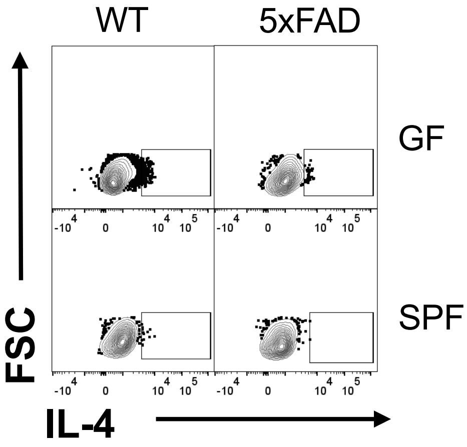

5xFAD 5M Females: SP
CD45^{+}CD4^{+}TCRβ^{+}Foxp3^{-}

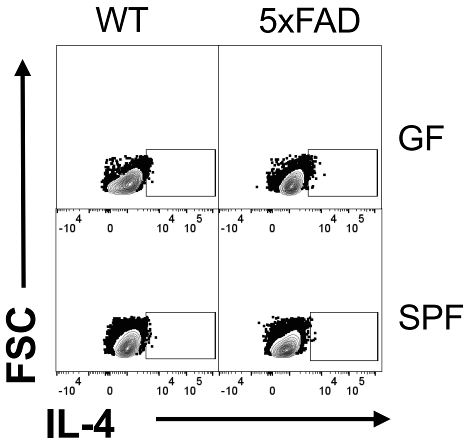

5xFAD 5M Females: MLN
CD45^{+}CD4^{+}TCRβ^{+}Foxp3^{-}

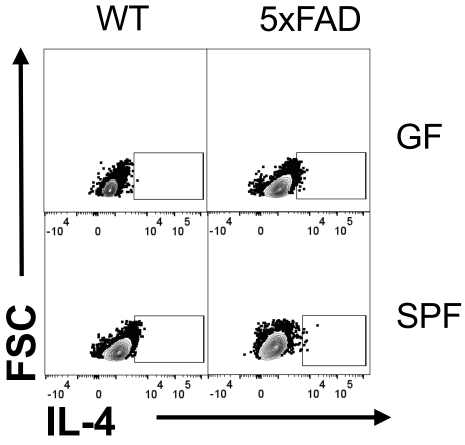

5xFAD 5M Females: SC
CD45^{+}CD4^{+}TCRβ^{+}Foxp3^{-}

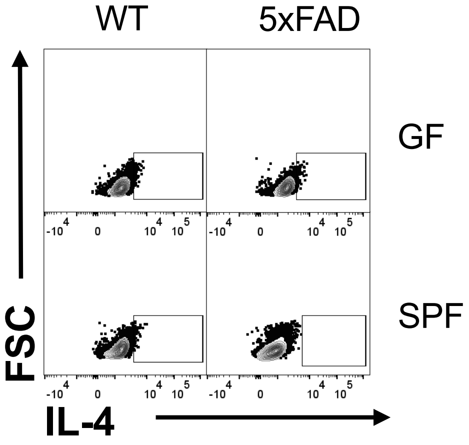

Representative Flow Cytometry Plots for Fig. S10: IL-4$^+$ MFI [5xFAD Males]

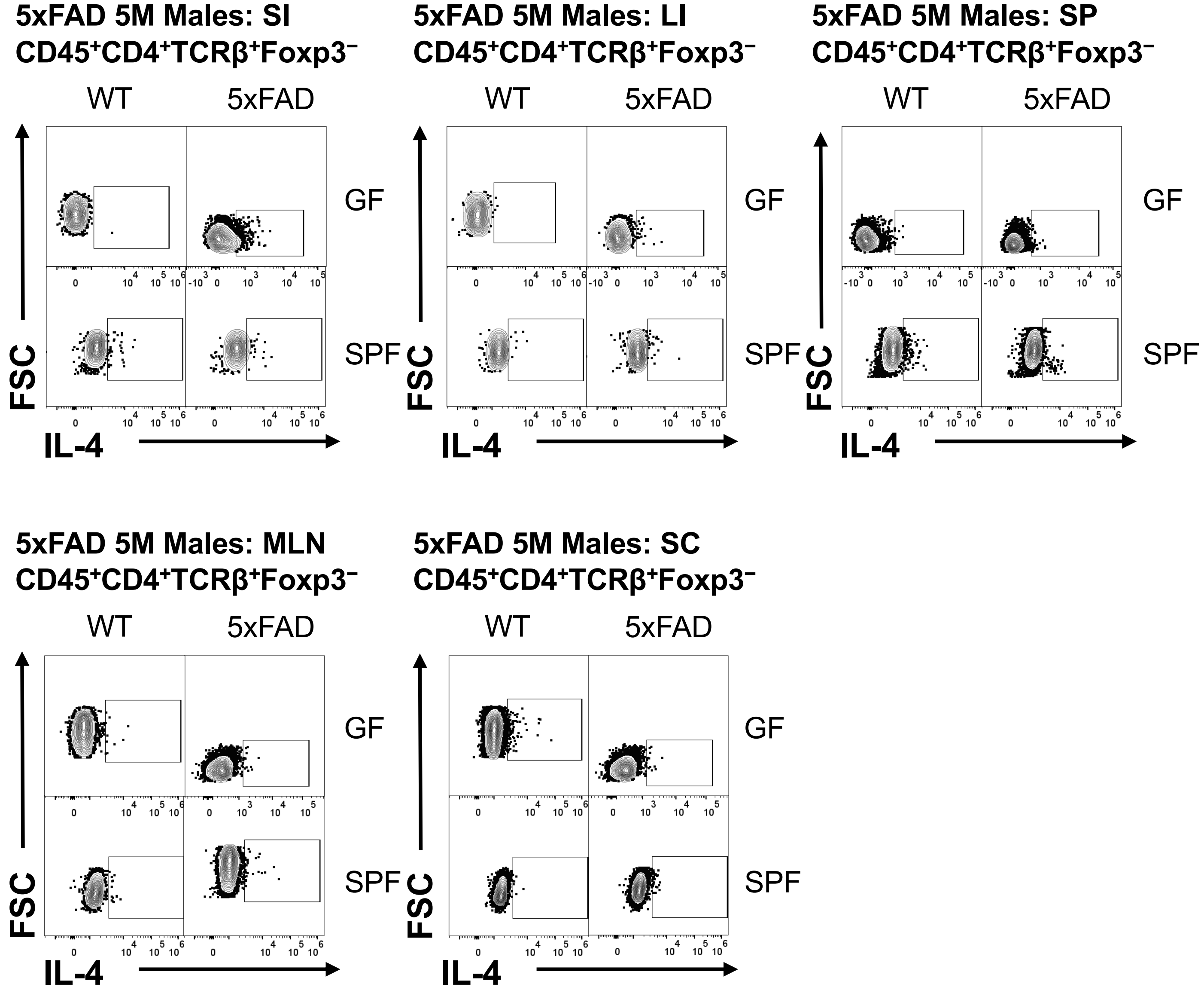

Representative Flow Cytometry Plots for Fig. S11: Foxp3$^+$ T cells [5xFAD Females]

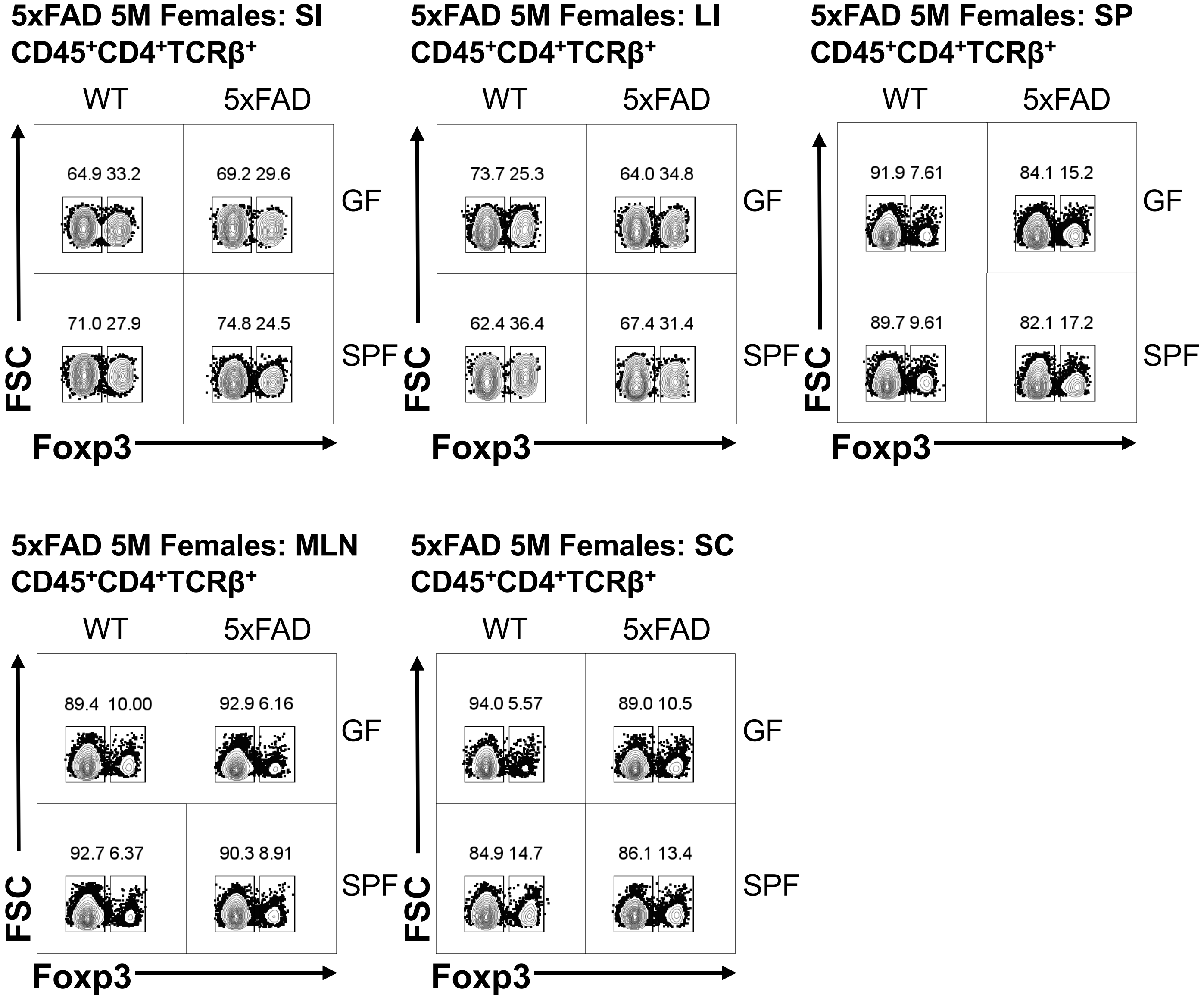

Representative Flow Cytometry Plots for Fig. S11: Foxp3$^+$ T cells [5xFAD Males]

5xFAD 5M Males: SI
CD45$^+$CD4$^+$TCRβ$^+$

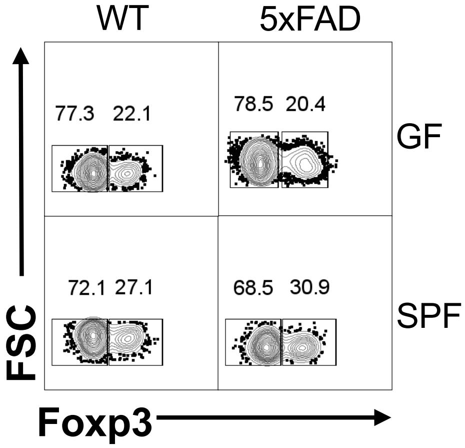

5xFAD 5M Males: LI
CD45$^+$CD4$^+$TCRβ^{+-}

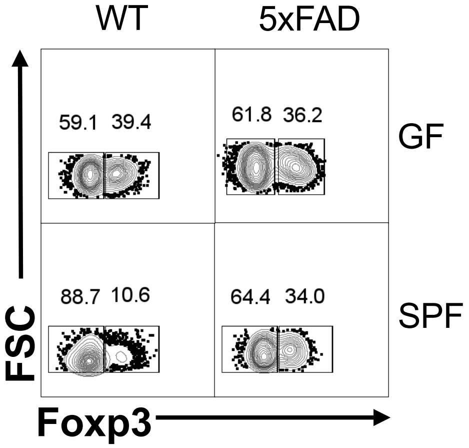

5xFAD 5M Males: SP
CD45$^+$CD4$^+$TCRβ$^+$

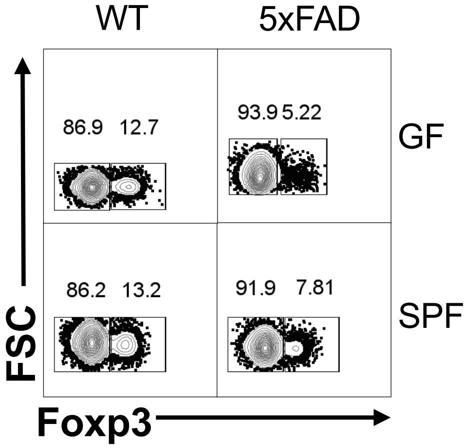

5xFAD 5M Males: MLN
CD45$^+$CD4$^+$TCRβ$^+$

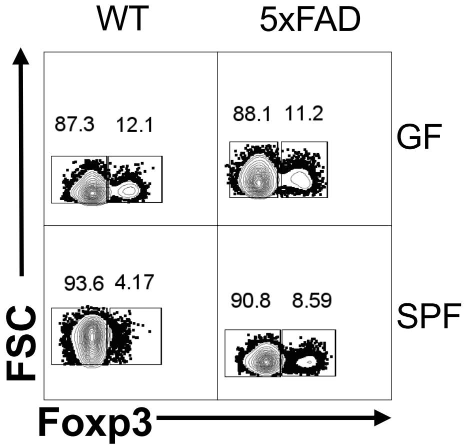

5xFAD 5M Males: SC
CD45$^+$CD4$^+$TCRβ$^+$

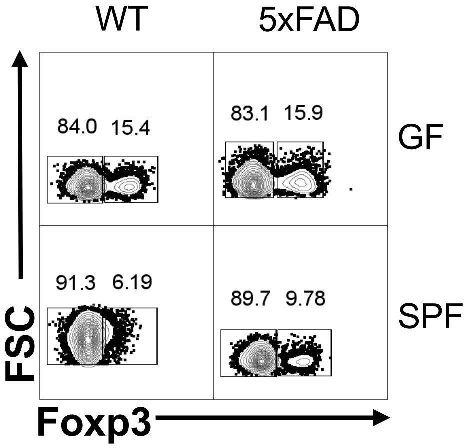

Representative Flow Cytometry Plots for Fig. S13: T and B cells [3xTg SPF Males]

3xTg SPF Males: SP
CD45⁺

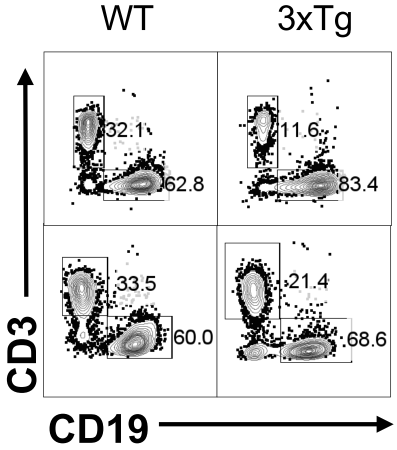

3xTg SPF Males: MLN
CD45⁺

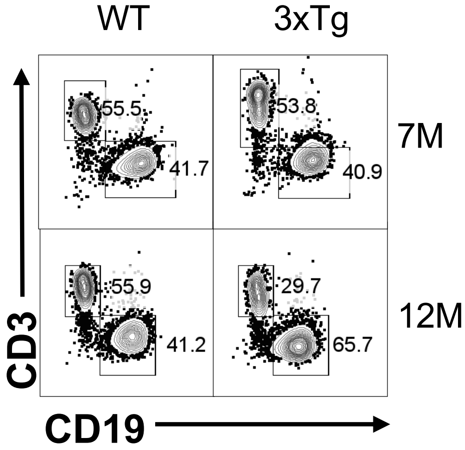

3xTg SPF Males: SC
CD45⁺

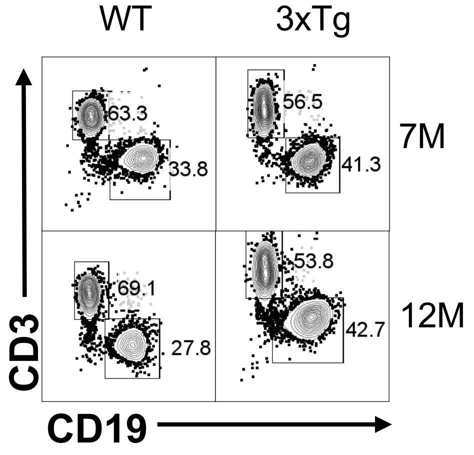

3xTg SPF Males: DC
CD45⁺

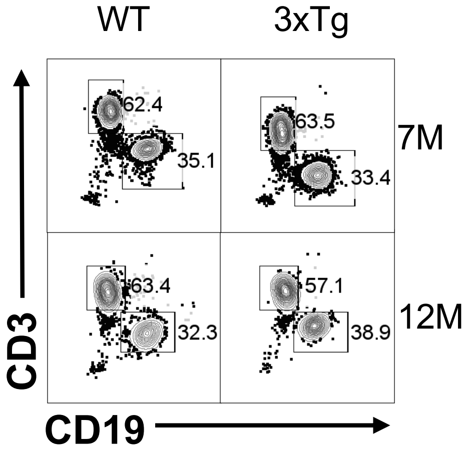

Representative Flow Cytometry Plots for Fig. S13: T and B cells [3xTg GF Females]

3xTg GF Females: SP
CD45⁺

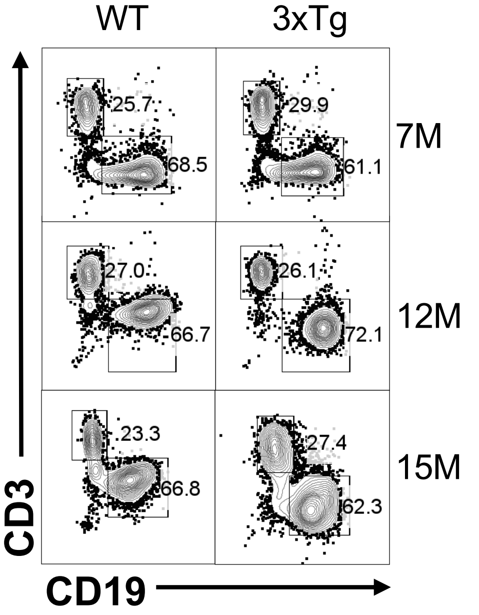

3xTg GF Females: MLN
CD45⁺

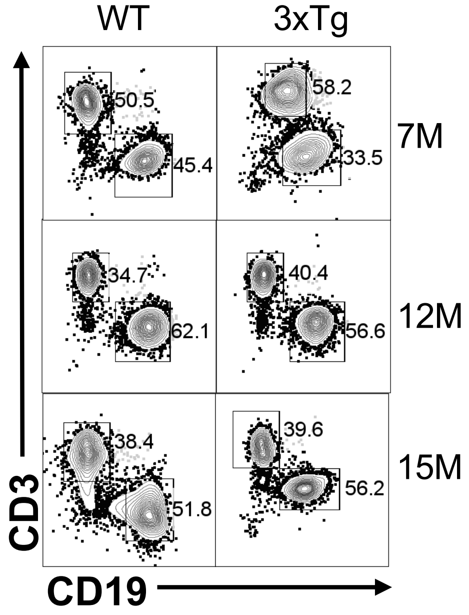

3xTg GF Females: SC
CD45⁺

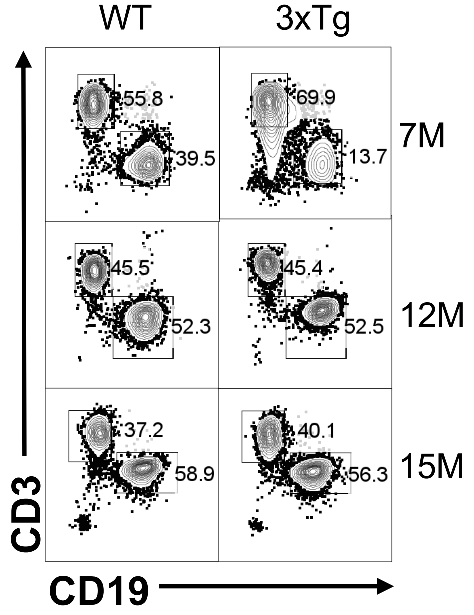

3xTg GF Females: DC
CD45⁺

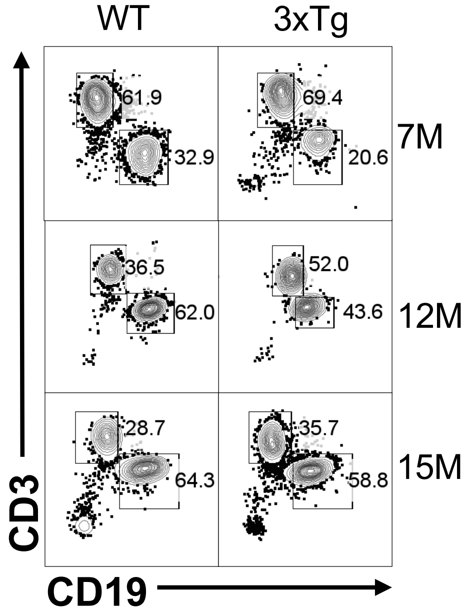

Representative Flow Cytometry Plots for Fig. S13: T and B cells [3xTg GF Males]

3xTg GF Males: SP
CD45⁺

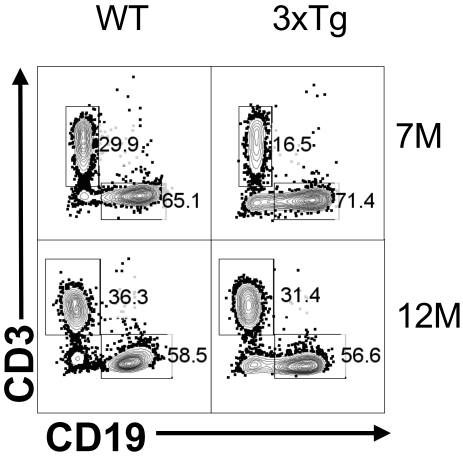

3xTg GF Males: MLN
CD45⁺

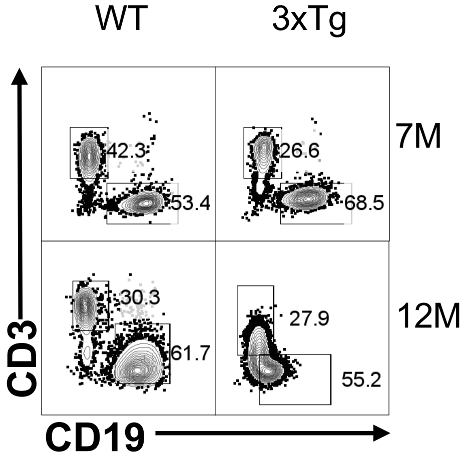

3xTg GF Males: SC
CD45⁺

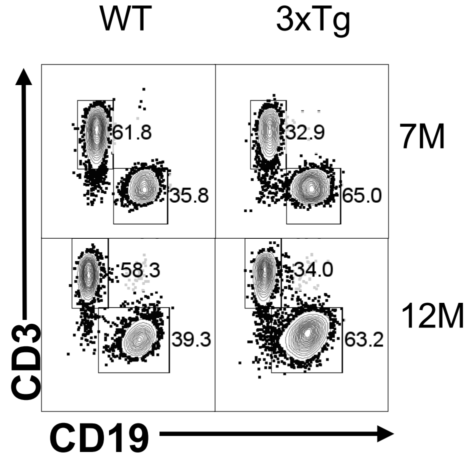

3xTg GF Males: DC
CD45⁺

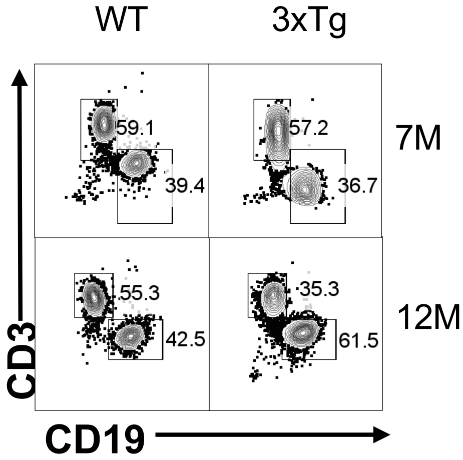

Representative Flow Cytometry Plots for Fig. S14: T and B cells [5xFAD 8M]

5xFAD 8M Females: SP
CD45+

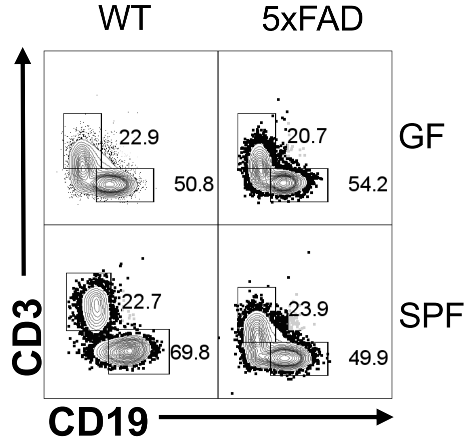

5xFAD 8M Females: MLN
CD45+

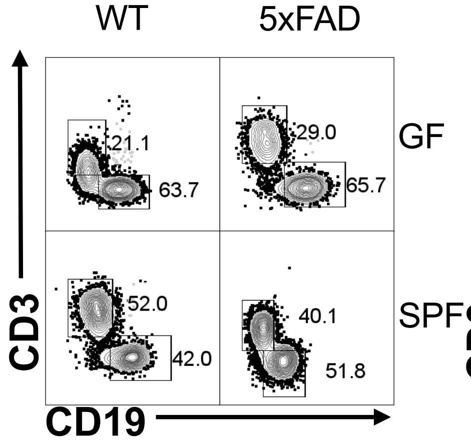

5xFAD 8M Females: SC
CD45+

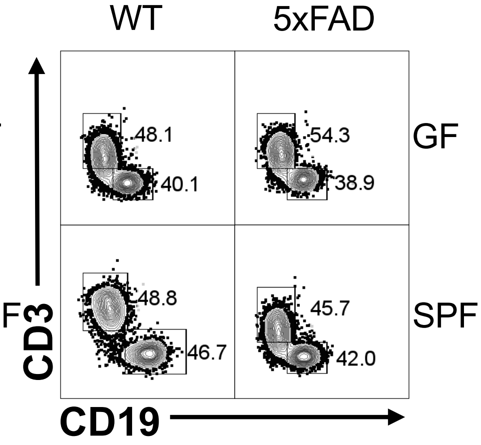

5xFAD 8M Females: DC
CD45+

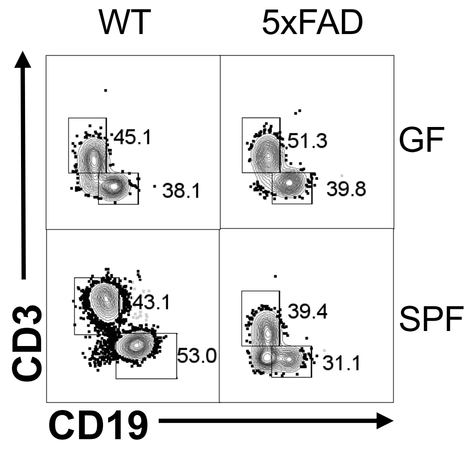

5xFAD 8M Males: SP
CD45+

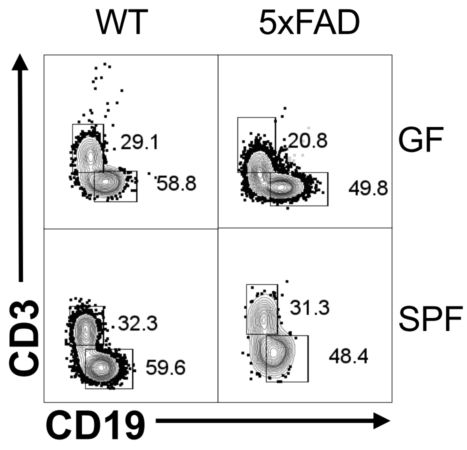

5xFAD 8M Males: MLN
CD45+

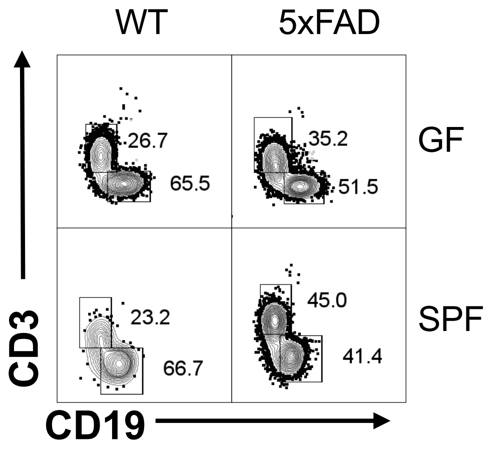

5xFAD 8M Males: SC
CD45+

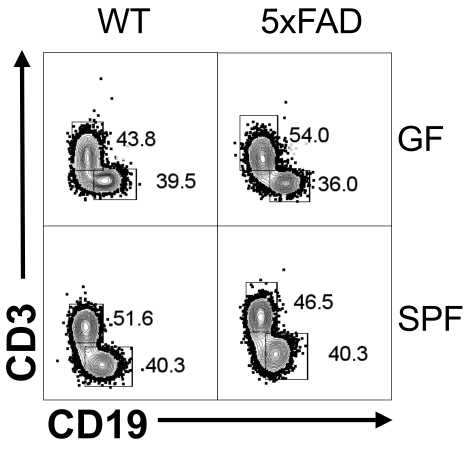

5xFAD 8M Males: DC
CD45+

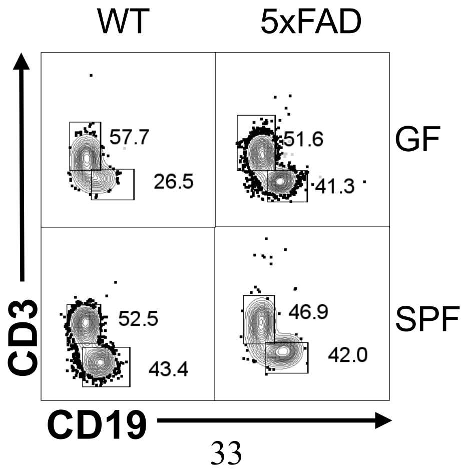

Supplement: MMC2 [file NIHMS2101848-supplement-MMC2.pdf]
