# Supplementary material for: Genotype and microbiome shape immunity in a sex-specific manner in mouse models of Alzheimer’s disease
Source: Brain Behav Immun. Author manuscript; Available in PMC 2025 Oct 2. (PMC12490345; doi:10.1016/j.bbi.2025.07.028)
Supplement: Supp.figures [file NIHMS2101848-supplement-Supp_figures.pdf]

Supplementary Figures

Genotype and microbiome shape immunity in a sex-specific manner in mouse models of Alzheimer's disease

John W. Bostick, T. Jaymie Connerly, Taren Thron, Brittany D. Needham, Matheus de Castro Fonseca, Rima Kaddurah-Daouk, Rob Knight, and Sarkis K. Mazmanian

Supplementary Figure 1.

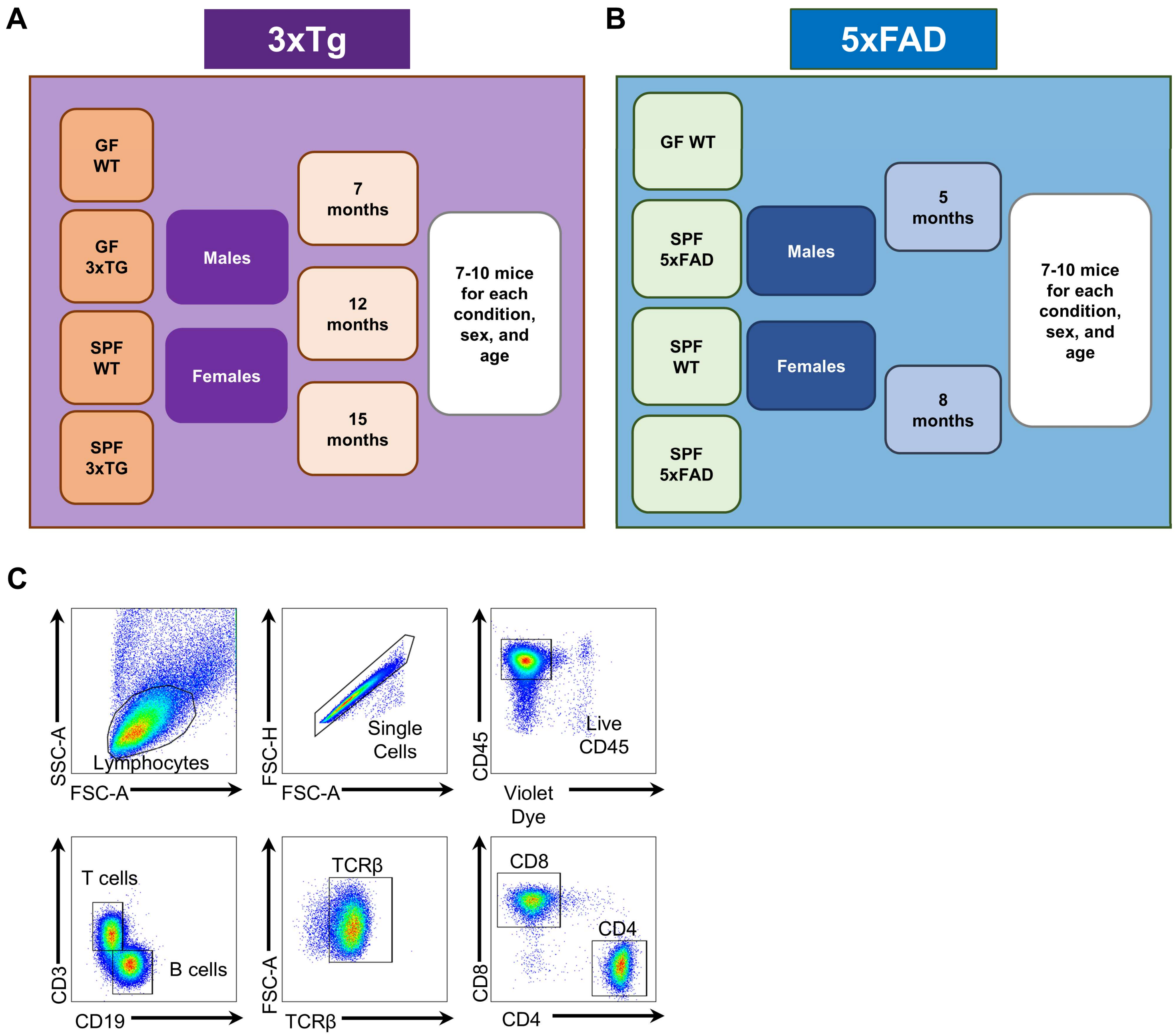

Supplementary Figure 1. Experimental design and flow cytometry gating.
(A) Illustration of experimental design for genotypes, housing conditions, ages, and sexes for 3xTg and (B) 5xFAD mice. (C) Flow cytometry gating strategy for T and B cell analysis.

Supplementary Figure 2.

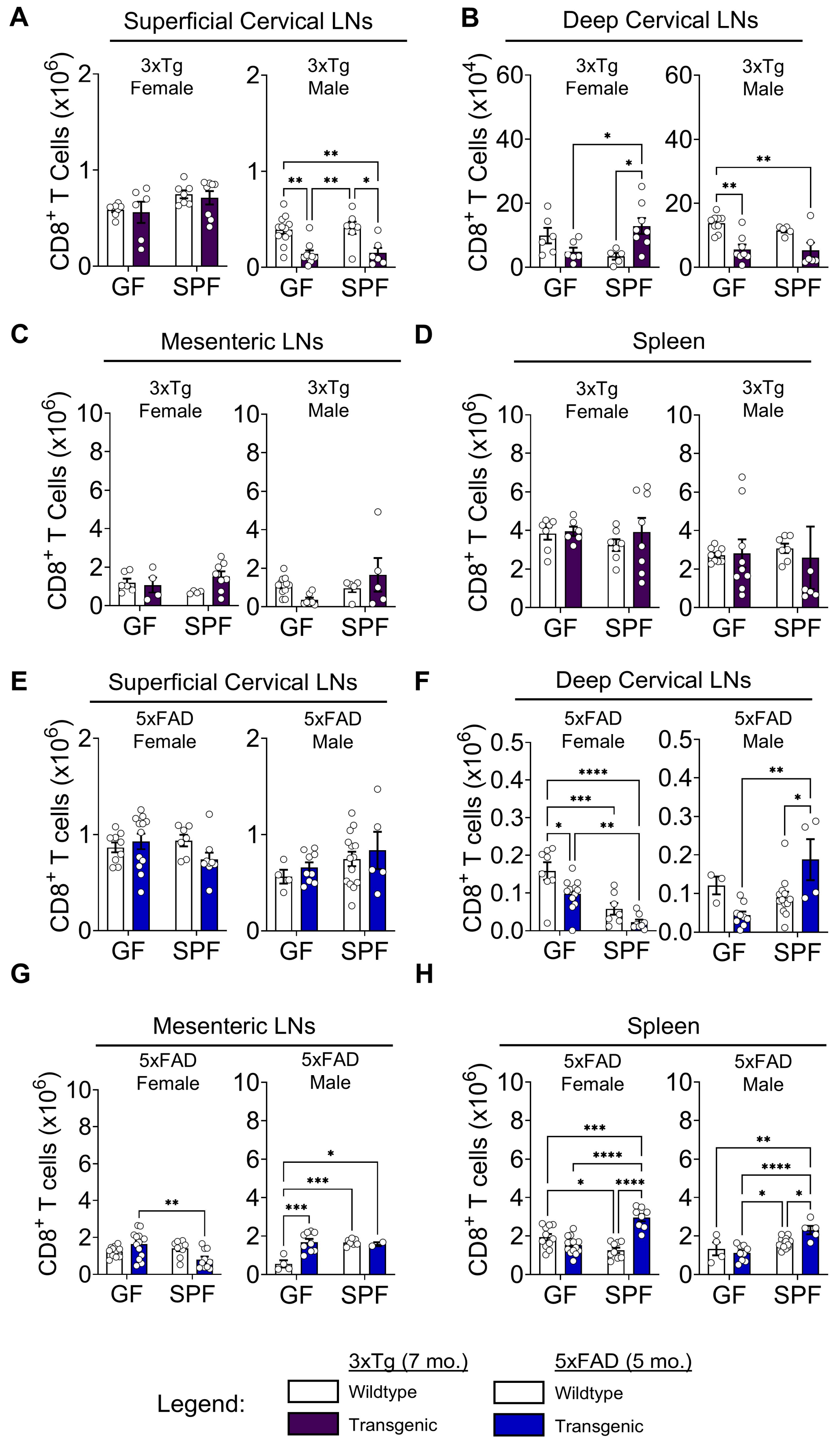

Supplementary Figure 2. T and B cell immune responses in the 3xTg and 5xFAD models.
(A) Quantification of $CD8^+$ T cell counts in the superficial cervical lymph nodes of female (Left) and male (Right) 3xTg mice compared to wildtype controls in germ-free (GF) and specific pathogen-free (SPF) conditions at 7 months of age. (B) Deep cervical lymph nodes. (C) Mesenteric lymph nodes. (D) Spleen. Data are pooled from 2 independent experiments (n = 4 to 12 per group); two-way ANOVA. (E) Quantification of $CD8^+$ T cell counts in the superficial cervical lymph nodes of female (Left) and male (Right) 5xFAD mice at 5 months of age. (F) Deep cervical lymph nodes. (G) Mesenteric lymph nodes. (H) Spleen. Data are pooled from 2 independent experiments (n = 2 to 14 per group); two-way ANOVA. Error bars indicate SEM. *P < 0.05, **P < 0.01, ***P < 0.001, ****P < 0.0001.

Supplementary Figure 3.

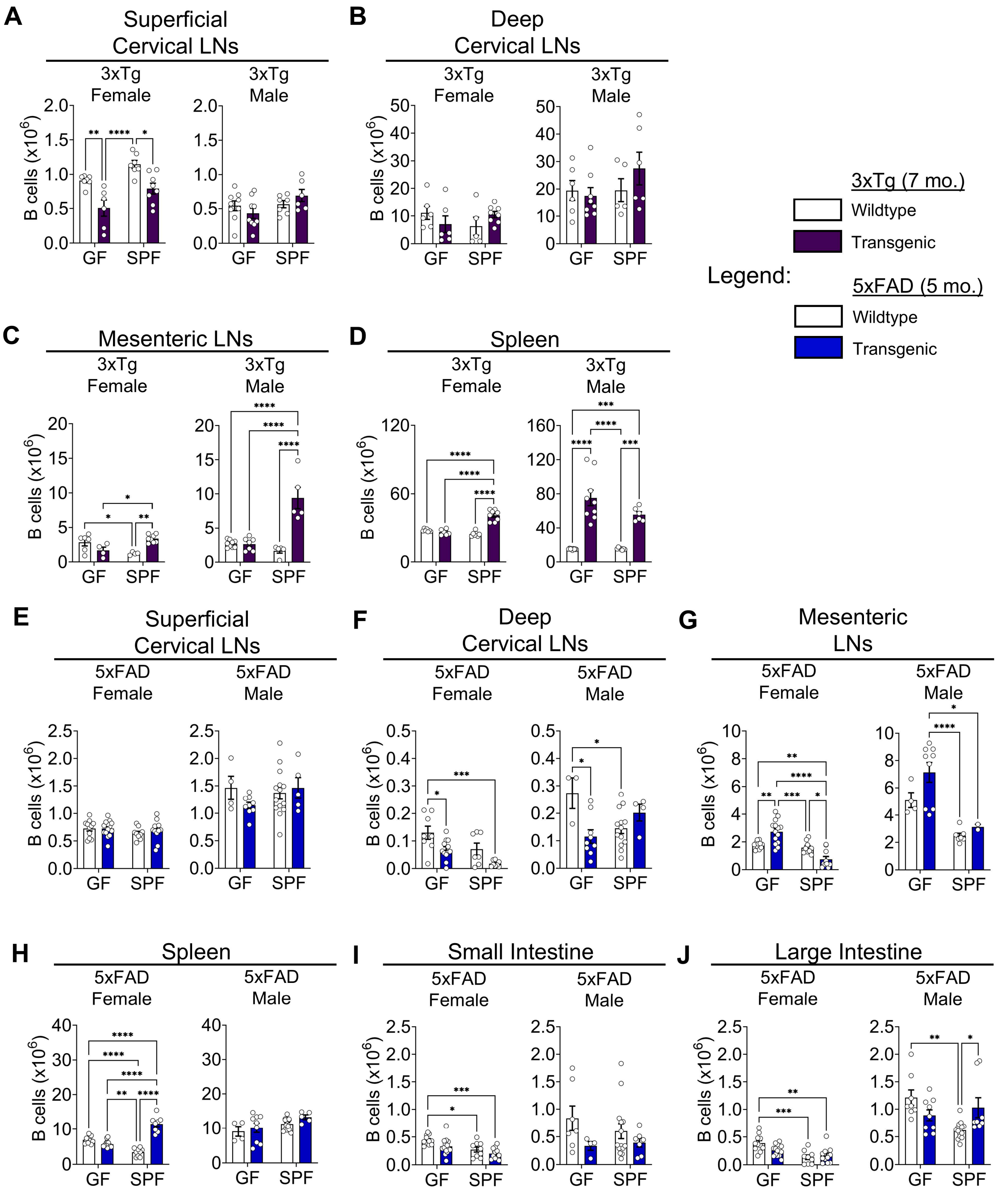

Supplementary Figure 3. B cell counts in 7-month-old 3xTg and 5-month-old 5xFAD mice.

(A) Quantification of B cell counts in the superficial cervical lymph nodes of female (Left) and male (Right) 3xTg mice compared to wildtype controls in germ-free (GF) and specific pathogen-free (SPF) conditions at 7 months of age. (B) Deep cervical lymph nodes. (C) Mesenteric lymph nodes. (D) Spleen. Data are pooled from 2 independent experiments (n = 4 to 14 per group); two-way ANOVA. (E) Quantification of B cell counts in the superficial cervical lymph nodes of female (Left) and male (Right) 5xFAD mice compared to wildtype controls in germ-free (GF) and specific pathogen-free (SPF) conditions at 5 months of age. (F) Deep cervical lymph nodes. (G) Mesenteric lymph nodes. (H) Spleen. (I) Small intestine. (J) Large intestine. Data are pooled from 2 independent experiments (n = 2 to 15 per group); two-way ANOVA. Error bars indicate SEM. *$P < 0.05$, **$P < 0.01$, ***$P < 0.001$, ****$P < 0.0001$.

Supplementary Figure 4.

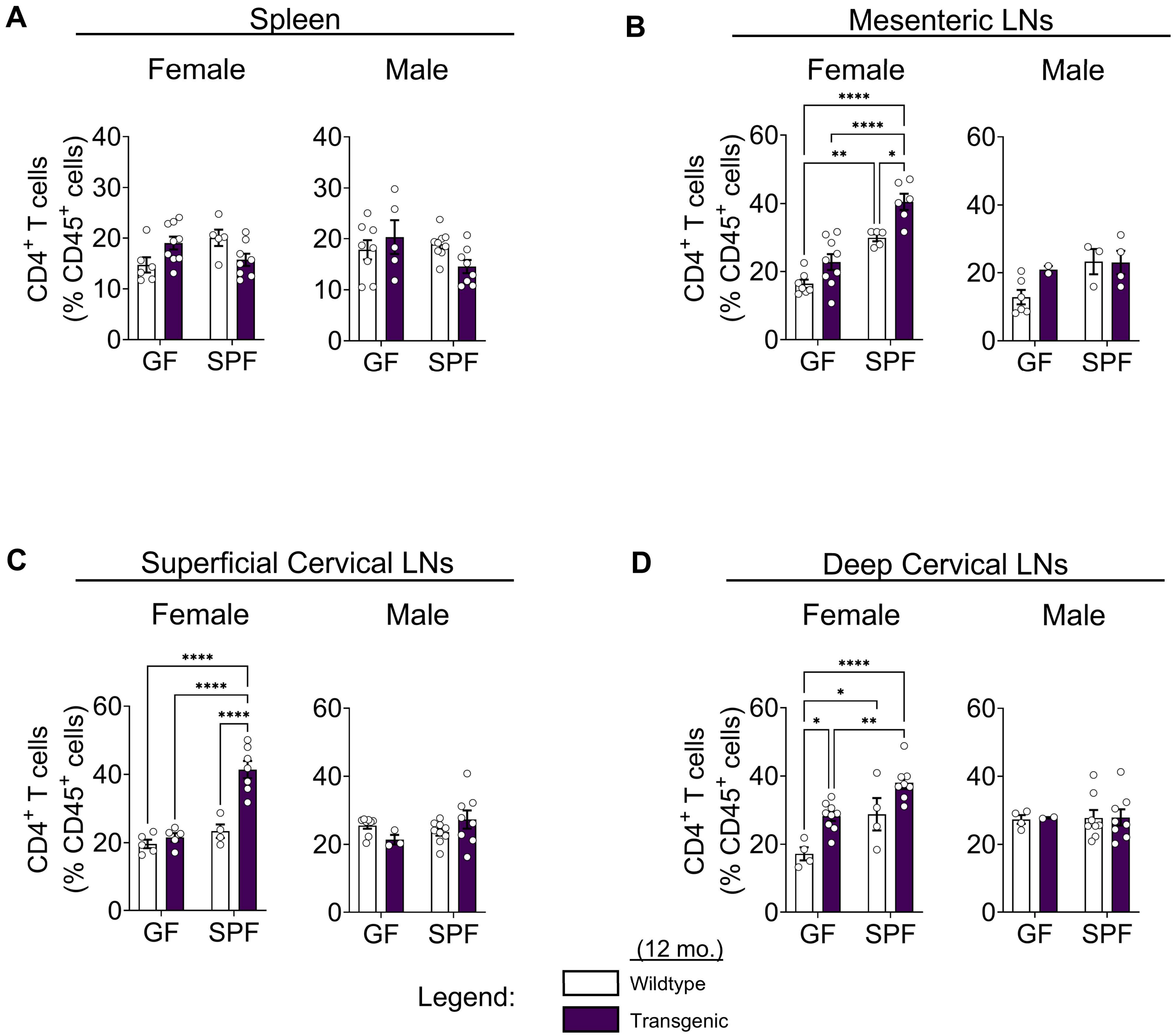

Supplementary Figure 4. Quantification of CD4$^+$ T cell frequencies in 12-month-old 3xTg mice.
(A) Quantification of CD4$^+$ T cell frequencies in the spleens of female (Left) and male (Right) 3xTg mice compared to wildtype controls in germ-free (GF) and specific pathogen-free (SPF) conditions at 12 months of age. (B) Mesenteric lymph nodes. (C) Superficial cervical lymph nodes. (D) Deep cervical lymph nodes. Data are pooled from 2 independent experiments (n = 2 to 9 per group); two-way ANOVA. Error bars indicate SEM. *P < 0.05, **P < 0.01, ***P < 0.001, ****P < 0.0001.

Supplementary Figure 5.

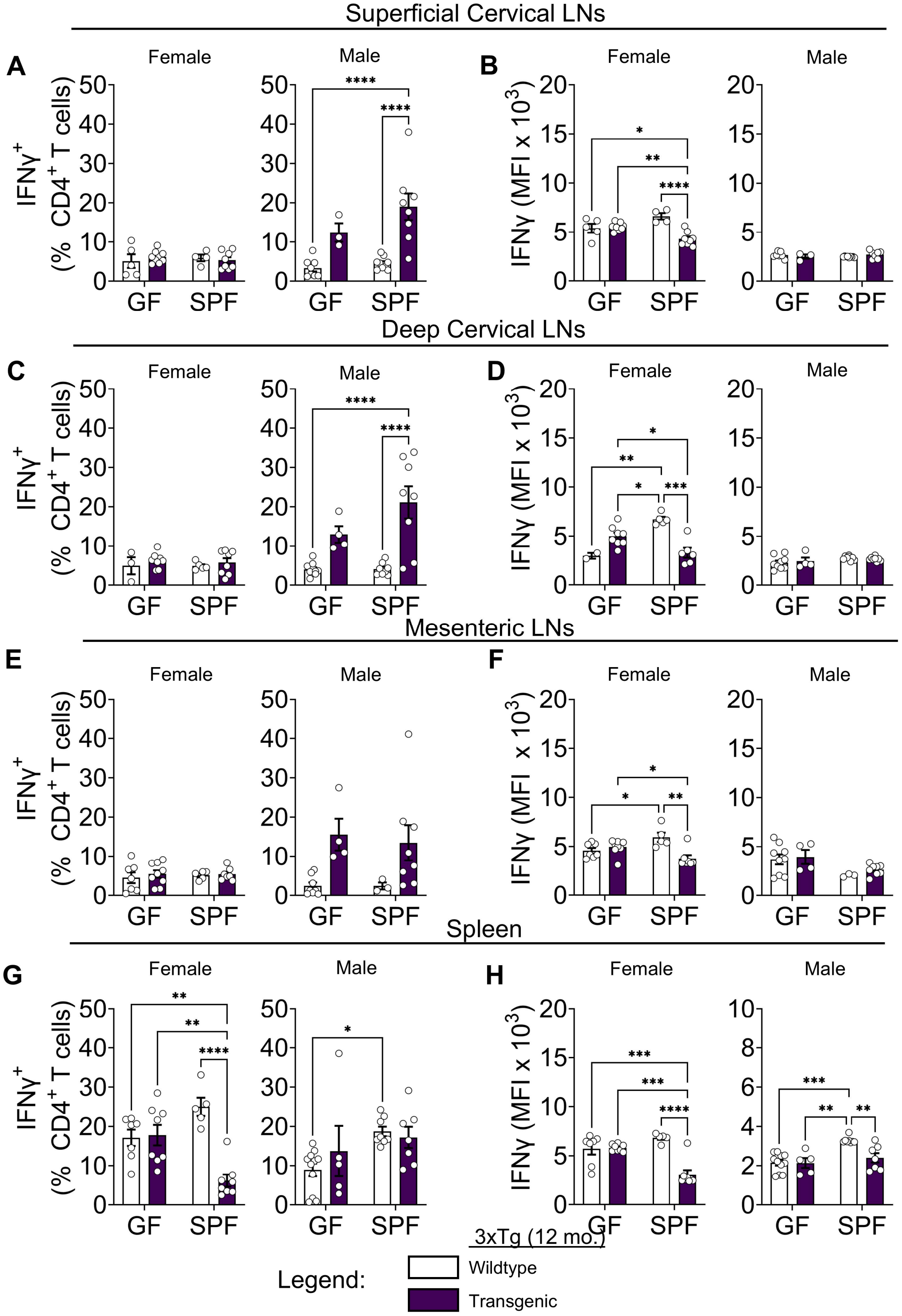

Supplementary Figure 5. Quantification of IFNγ$^+$CD4$^+$ T cells in 12-month-old 3xTg mice.
(A) Quantification of IFNγ$^+$ T cell frequencies and (B) IFNγ mean fluorescence intensity (MFI) in the superficial cervical lymph nodes of female (Left) and male (Right) 3xTg mice compared to wildtype controls in germ-free (GF) and specific pathogen-free (SPF) conditions at 12 months of age. (C) Frequencies and (D) MFI for deep cervical lymph nodes. (E) Frequencies and (F) MFI for mesenteric lymph nodes. (G) Frequencies and (H) MFI for spleen. Data are pooled from 2 independent experiments (n = 3 to 10 per group); two-way ANOVA. Cells were stimulated using phorbol 12-myristate 13-acetate (PMA) and ionomycin in the presence of Brefeldin A prior to intracellular cytokine staining. Error bars indicate SEM. *P < 0.05, **P < 0.01, ***P < 0.001, ****P < 0.0001.

Supplementary Figure 6.

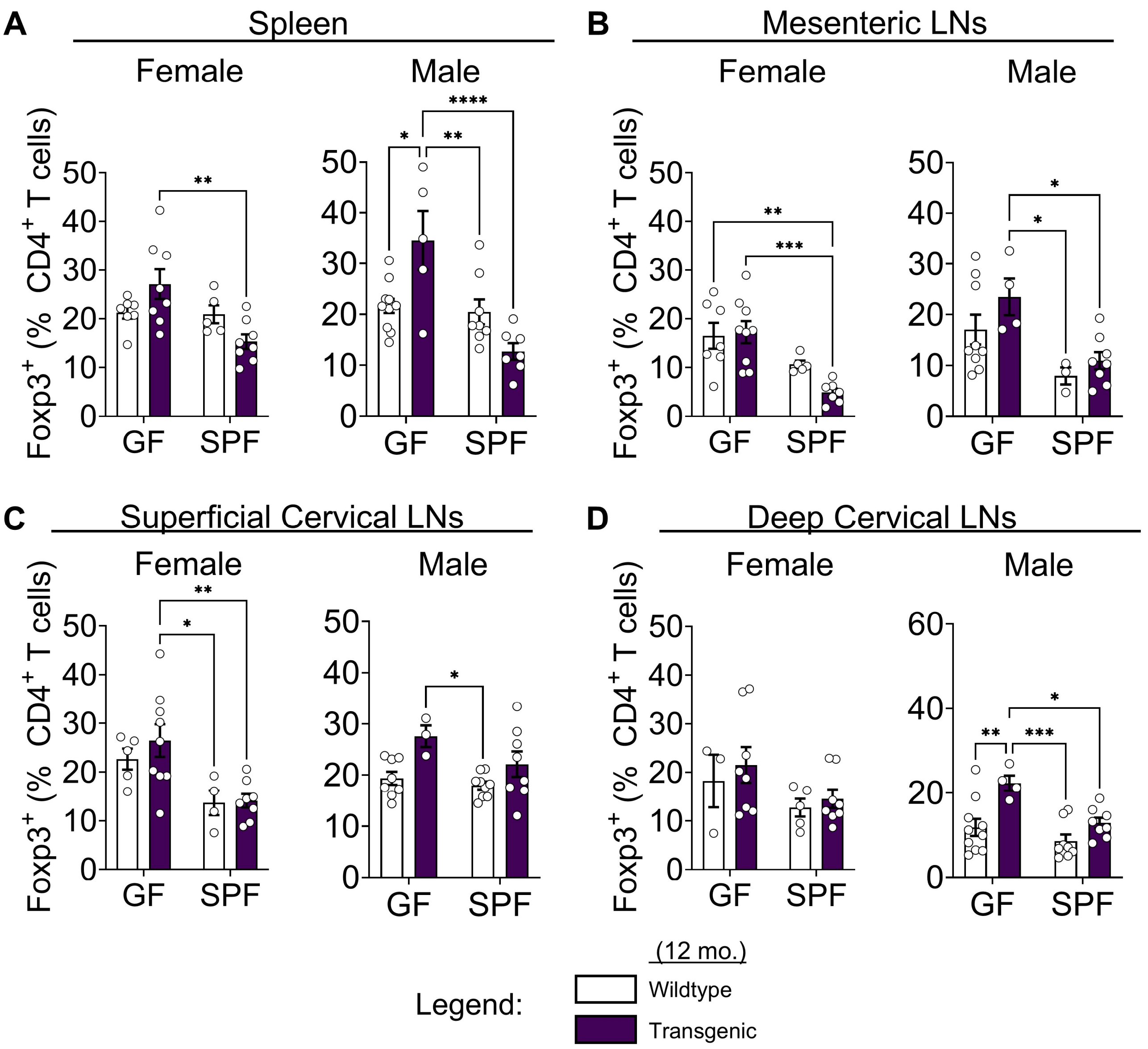

Supplementary Figure 6. Quantification of Foxp3$^+$ T cell frequencies in 12-month-old 3xTg mice. (A) Quantification of Foxp3$^+$ T cell frequencies in the spleens of female (Left) and male (Right) 3xTg mice compared to wildtype controls in germ-free (GF) and specific pathogen-free (SPF) conditions at 12 months of age. (B) Mesenteric lymph nodes. (C) Superficial cervical lymph nodes. (D) Deep cervical lymph nodes. Data are pooled from 2 independent experiments (n = 2 to 11 per group); two-way ANOVA. Error bars indicate SEM. *P < 0.05, **P < 0.01, ***P < 0.001, ****P < 0.0001.

Supplementary Figure 7.

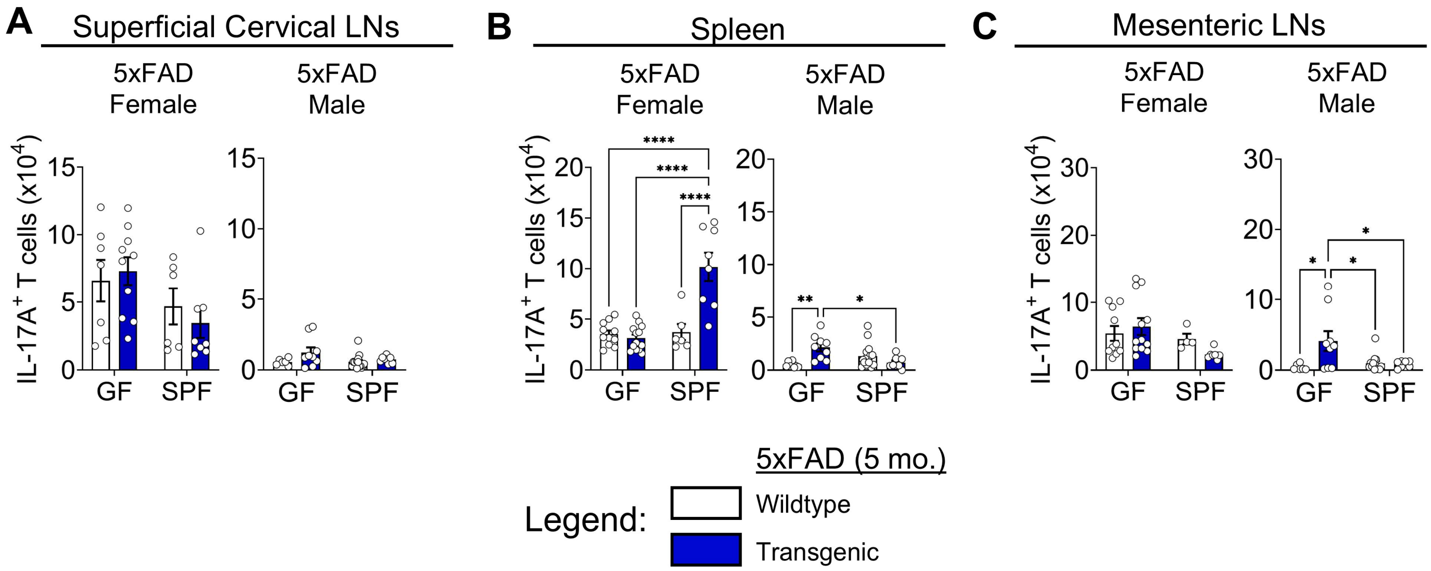

Supplementary Figure 7. IL-17A$^+$ T cell counts in 5xFAD mice.
(A) Quantification of IL-17A$^+$ T cell counts in the superficial cervical lymph nodes of female (Left) and male (Right) 5xFAD mice compared to wildtype controls in germ-free (GF) and specific pathogen-free (SPF) conditions at 5 months of age. (B) Spleen. (C) Mesenteric lymph nodes. Data are pooled from 2 independent experiments (n = 4 to 15 per group); two-way ANOVA. Cells were stimulated using PMA and ionomycin in the presence of Brefeldin A prior to intracellular cytokine staining. Error bars indicate SEM. *P < 0.05, **P < 0.01, ***P < 0.001, ****P < 0.0001.

Supplementary Figure 8.

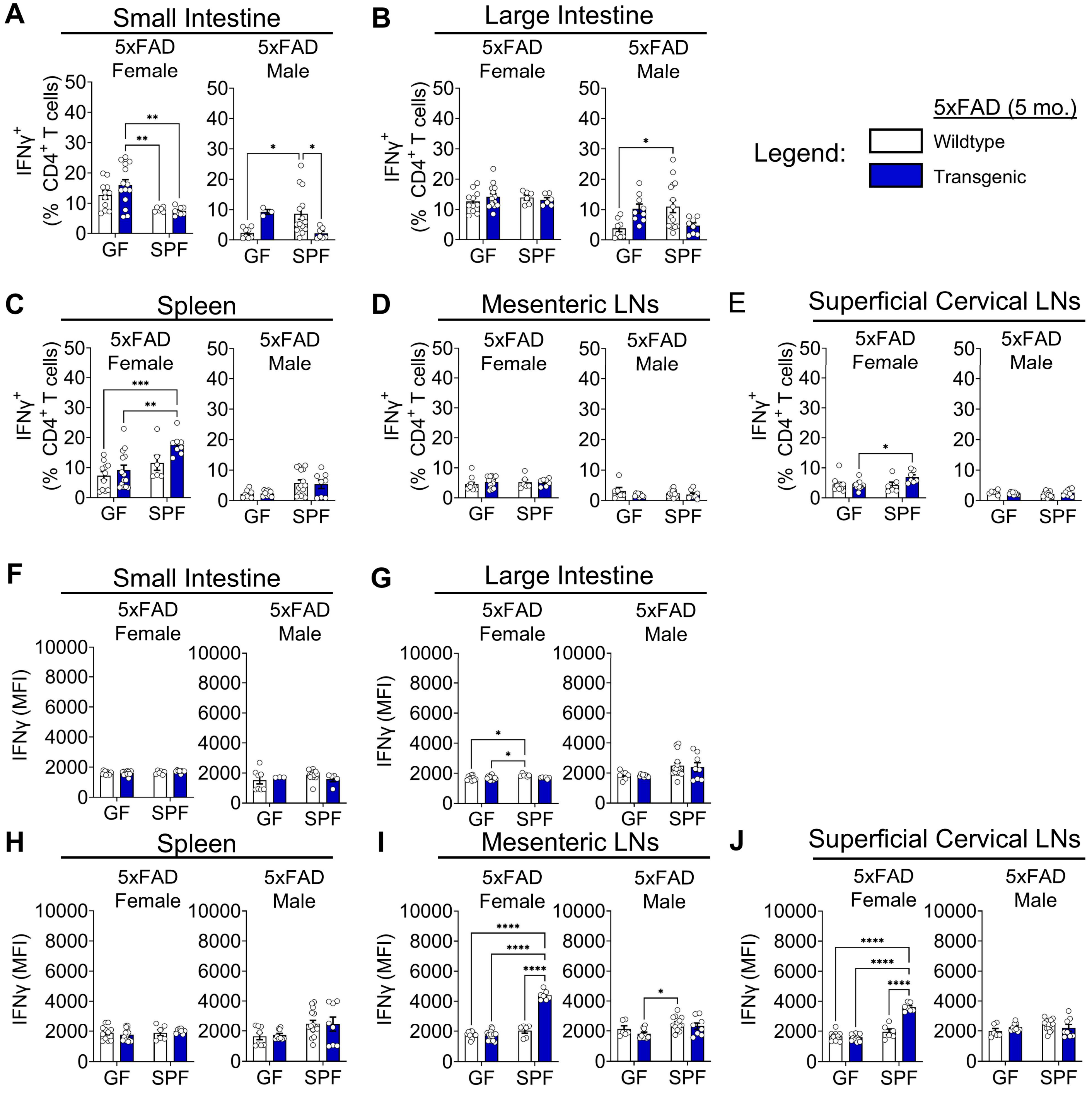

Supplementary Figure 8. Quantification of IFNγ$^+$CD4$^+$ T cells in 5-month-old 5xFAD mice.
(A) Quantification of IFNγ$^+$ T cell frequencies in the small intestines of female (Left) and male (Right) 5xFAD mice compared to wildtype controls in germ-free (GF) and specific pathogen-free (SPF) conditions at 5 months of age. (B) Large intestine. (C) Spleen. (D) Mesenteric lymph nodes. (E) Superficial cervical lymph nodes. (F) Quantification of IFNγ mean fluorescence intensity (MFI) expressed by CD4$^+$ T cells in the small intestine. (G) Large intestine. (H) Spleen. (I) Mesenteric lymph nodes. (J) Superficial cervical lymph nodes. Data are pooled from 2 independent experiments (n = 4 to 14 per group); two-way ANOVA. Cells were stimulated using PMA and ionomycin in the presence of Brefeldin A prior to intracellular cytokine staining. Error bars indicate SEM. *$P < 0.05$, **$P < 0.01$, ***$P < 0.001$, ****$P < 0.0001$.

Supplementary Figure 9.

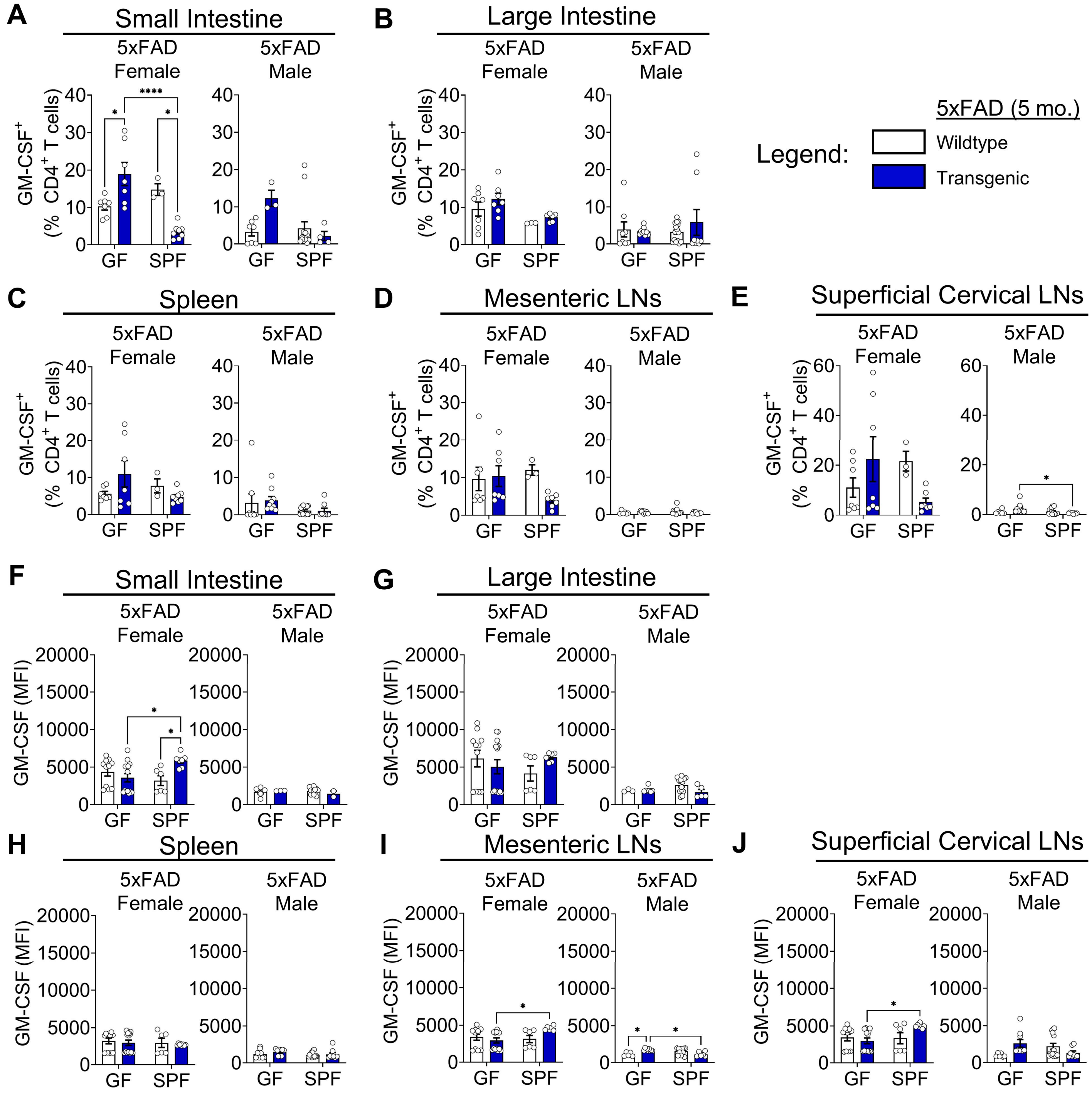

Supplementary Figure 9. Quantification of GM-CSF$^+$CD4$^+$ T cells in 5-month-old 5xFAD mice. (A) Quantification of GM-CSF$^+$ T cell frequencies in the small intestines of female (Left) and male (Right) 5xFAD mice compared to wildtype controls in germ-free (GF) and specific pathogen-free (SPF) conditions at 5 months of age. (B) Large intestine. (C) Spleen. (D) Mesenteric lymph nodes. (E) Superficial cervical lymph nodes. (F) Quantification of GM-CSF mean fluorescence intensity (MFI) expressed by CD4$^+$ T cells in the small intestine. (G) Large intestine. (H) Spleen. (I) Mesenteric lymph nodes. (J) Superficial cervical lymph nodes. Data are pooled from 2 independent experiments (n = 4 to 14 per group); two-way ANOVA. Cells were stimulated using PMA and ionomycin in the presence of Brefeldin A prior to intracellular cytokine staining. Error bars indicate SEM. *P < 0.05, **P < 0.01, ***P < 0.001, ****P < 0.0001.

Supplementary Figure 10.

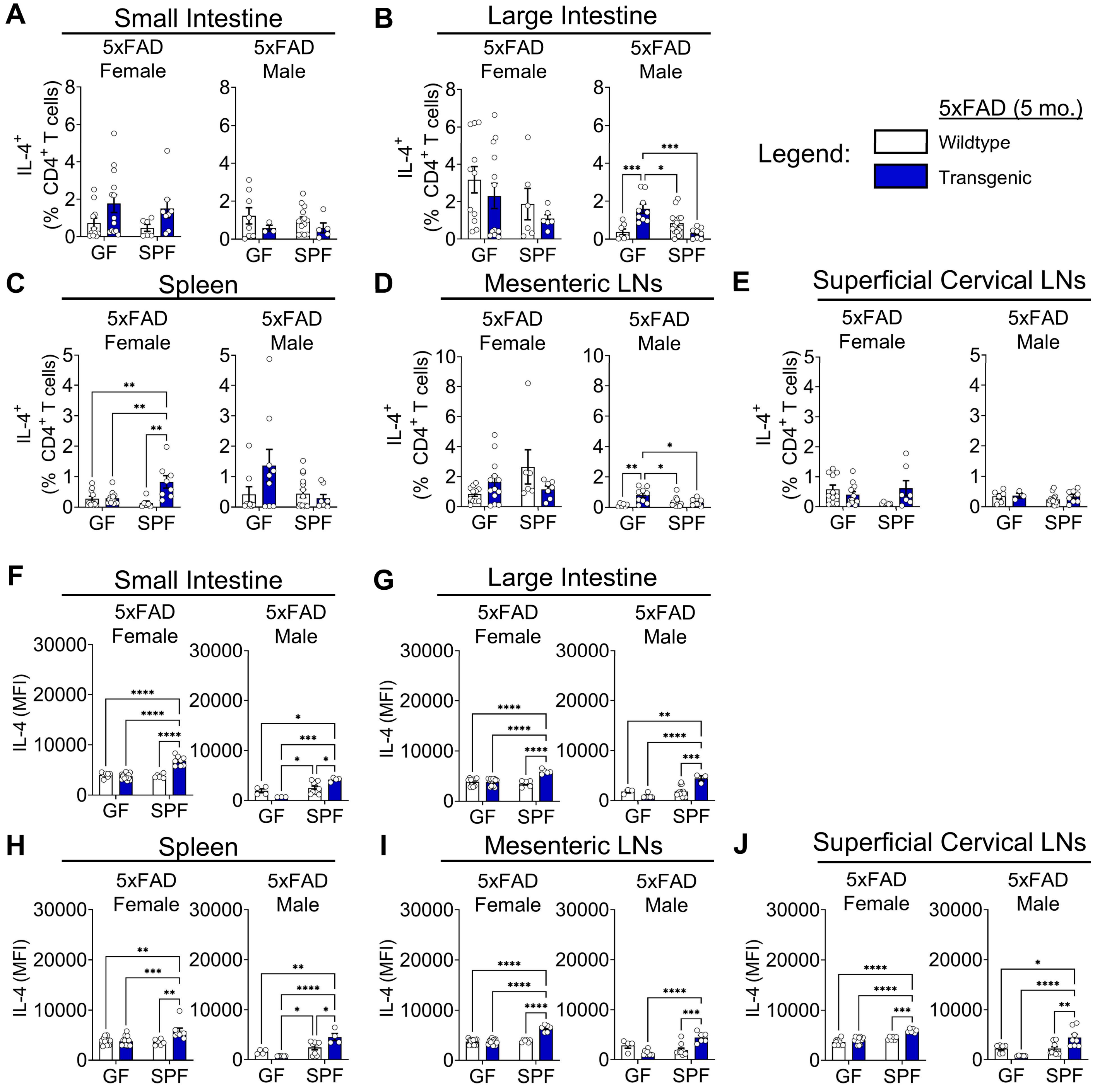

Supplementary Figure 10. Quantification of IL-4$^+$CD4$^+$ T cells in 5-month-old 5xFAD mice.
(A) Quantification of IL-4$^+$ T cell frequencies in the small intestines of female (Left) and male (Right) 5xFAD mice compared to wildtype controls in germ-free (GF) and specific pathogen-free (SPF) conditions at 5 months of age. (B) Large intestine. (C) Spleen. (D) Mesenteric lymph nodes. (E) Superficial cervical lymph nodes. (F) Quantification of IL-4 mean fluorescence intensity (MFI) expressed by CD4$^+$ T cells in the small intestine. (G) Large intestine. (H) Spleen. (I) Mesenteric lymph nodes. (J) Superficial cervical lymph nodes. Data are pooled from 2 independent experiments (n = 4 to 14 per group); two-way ANOVA. Cells were stimulated using PMA and ionomycin in the presence of Brefeldin A prior to intracellular cytokine staining. Error bars indicate SEM. *$P < 0.05$, **$P < 0.01$, ***$P < 0.001$, ****$P < 0.0001$.

Supplementary Figure 11.

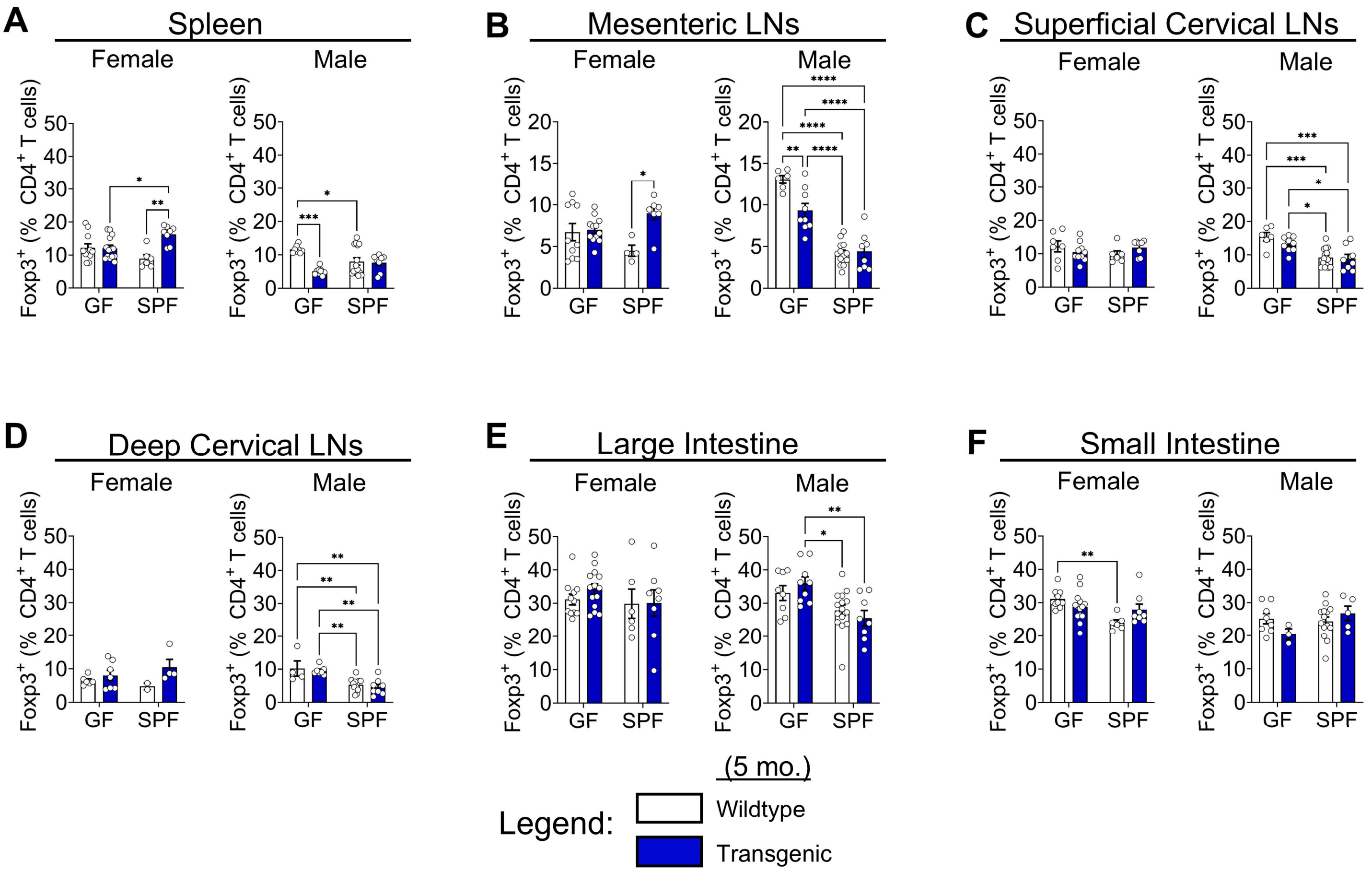

Supplementary Figure 11. Quantification of Foxp3$^+$ T cell frequencies in 5-month-old 5xFAD mice. (A) Quantification of Foxp3$^+$ T cell frequencies in the spleens of female (Left) and male (Right) 5xFAD mice compared to wildtype controls in germ-free (GF) and specific pathogen-free (SPF) conditions at 5 months of age. (B) Mesenteric lymph nodes. (C) Superficial cervical lymph nodes. (D) Deep cervical lymph nodes. (E) Large intestine. (F) Small intestine. Data are pooled from 2 independent experiments (n = 2 to 15 per group); two-way ANOVA. Error bars indicate SEM. *P < 0.05, **P < 0.01, ***P < 0.001, ****P < 0.0001.

Supplementary Figure 12.

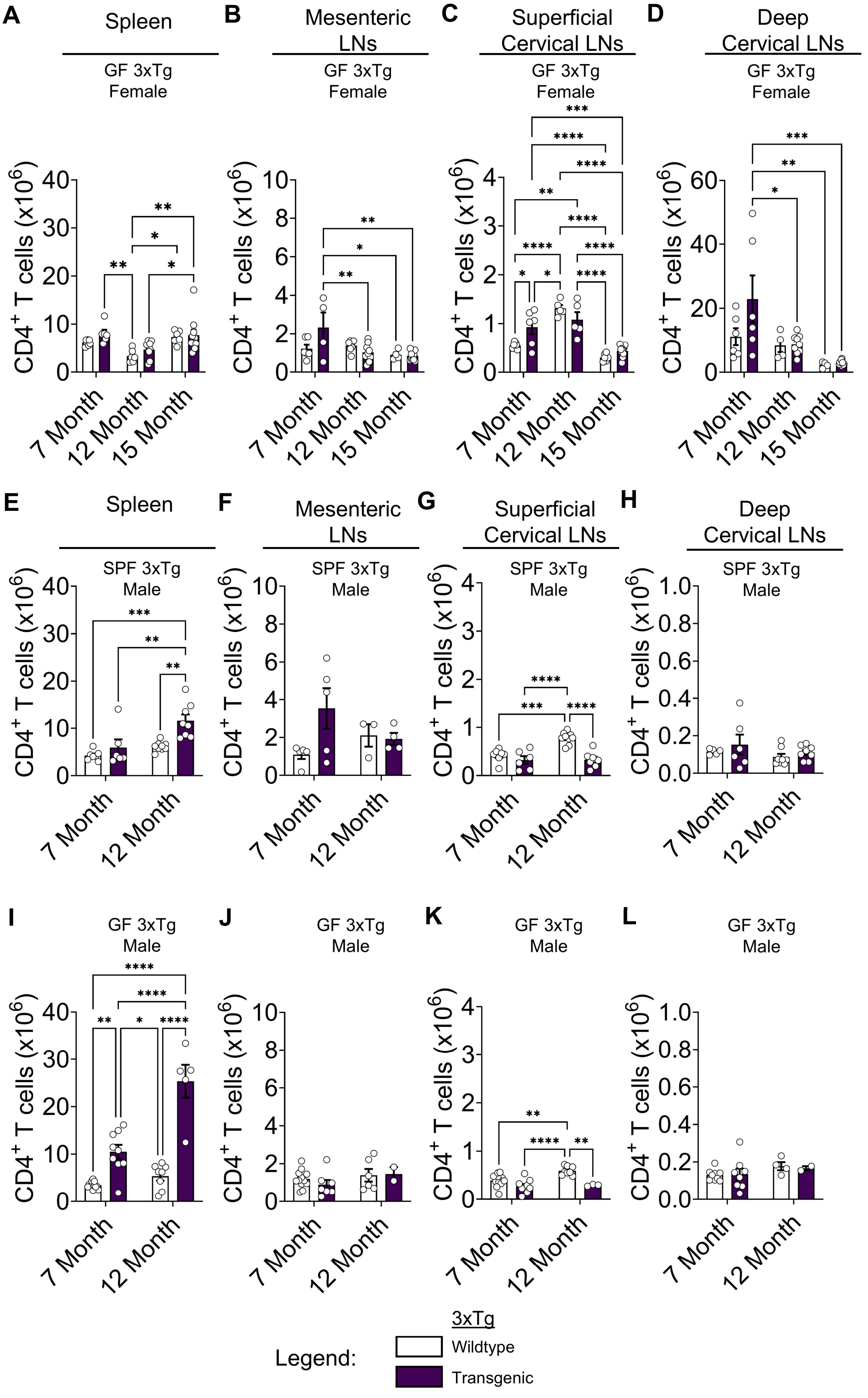

Supplementary Figure 12. Longitudinal $CD4^+$ T cell responses in 3xTg mice.
(A) Quantification of $CD4^+$ T cell counts in the spleens of female 3xTg mice compared to wildtype controls in germ free (GF) conditions at 7-, 12-, and 15-month ages. (B) Mesenteric lymph nodes. (C) Superficial cervical lymph nodes. (D) Deep cervical lymph nodes. Data are pooled from 5 independent experiments (n = 4 to 10 per group); two-way ANOVA. (E) Quantification of $CD4^+$ T cell counts in the spleens of specific pathogen-free (SPF) male 3xTg mice at 7- and 12-month ages. (F) Mesenteric lymph nodes. (G) Superficial cervical lymph nodes. (H) Deep cervical lymph nodes. Data are pooled from 5 independent experiments (n = 3 to 9 per group); two-way ANOVA. (I) Quantification of $CD4^+$ T cell counts in the spleens of GF male 3xTg mice at 7- and 12-month ages. (J) Mesenteric lymph nodes. (K) Superficial cervical lymph nodes. (L) Deep cervical lymph nodes. Data are pooled from 5 independent experiments (n = 2 to 9 per group); two-way ANOVA. Error bars indicate SEM. *P < 0.05, **P < 0.01, ***P < 0.001, ****P < 0.0001.

Supplementary Figure 13.

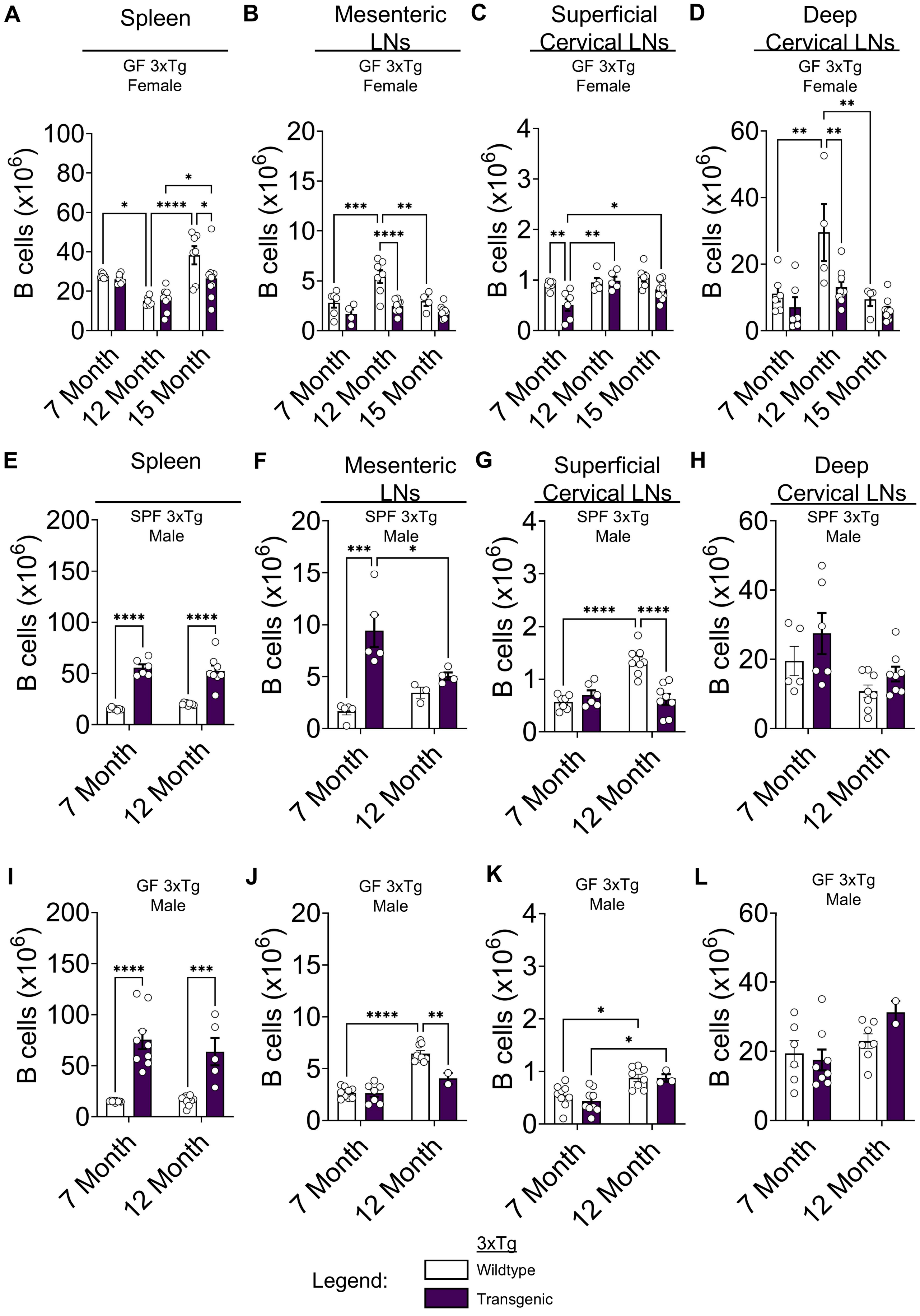

Supplementary Figure 13. Longitudinal B cell responses in 3xTg mice.
(A) Quantification of B cell counts in the spleens of female 3xTg mice compared to wildtype controls in germ free (GF) conditions at 7-, 12-, and 15-month ages. (B) Mesenteric lymph nodes. (C) Superficial cervical lymph nodes. (D) Deep cervical lymph nodes. Data are pooled from 5 independent experiments (n = 4 to 10 per group); two-way ANOVA. (E) Quantification of B cell counts in the spleens of specific pathogen-free (SPF) male 3xTg mice at 7- and 12-month ages. (F) Mesenteric lymph nodes. (G) Superficial cervical lymph nodes. (H) Deep cervical lymph nodes. Data are pooled from 5 independent experiments (n = 3 to 9 per group); two-way ANOVA. (I) Quantification of B cell counts in the spleens of GF male 3xTg mice at 7- and 12-month ages. (J) Mesenteric lymph nodes. (K) Superficial cervical lymph nodes. (L) Deep cervical lymph nodes. Data are pooled from 5 independent experiments (n = 2 to 11 per group); two-way ANOVA. For clarity, only significant comparisons of the same mouse group between ages or between transgenic and control mice at the same age are shown. Error bars indicate SEM. *P < 0.05, **P < 0.01, ***P < 0.001, ****P < 0.0001.

Supplementary Figure 14.

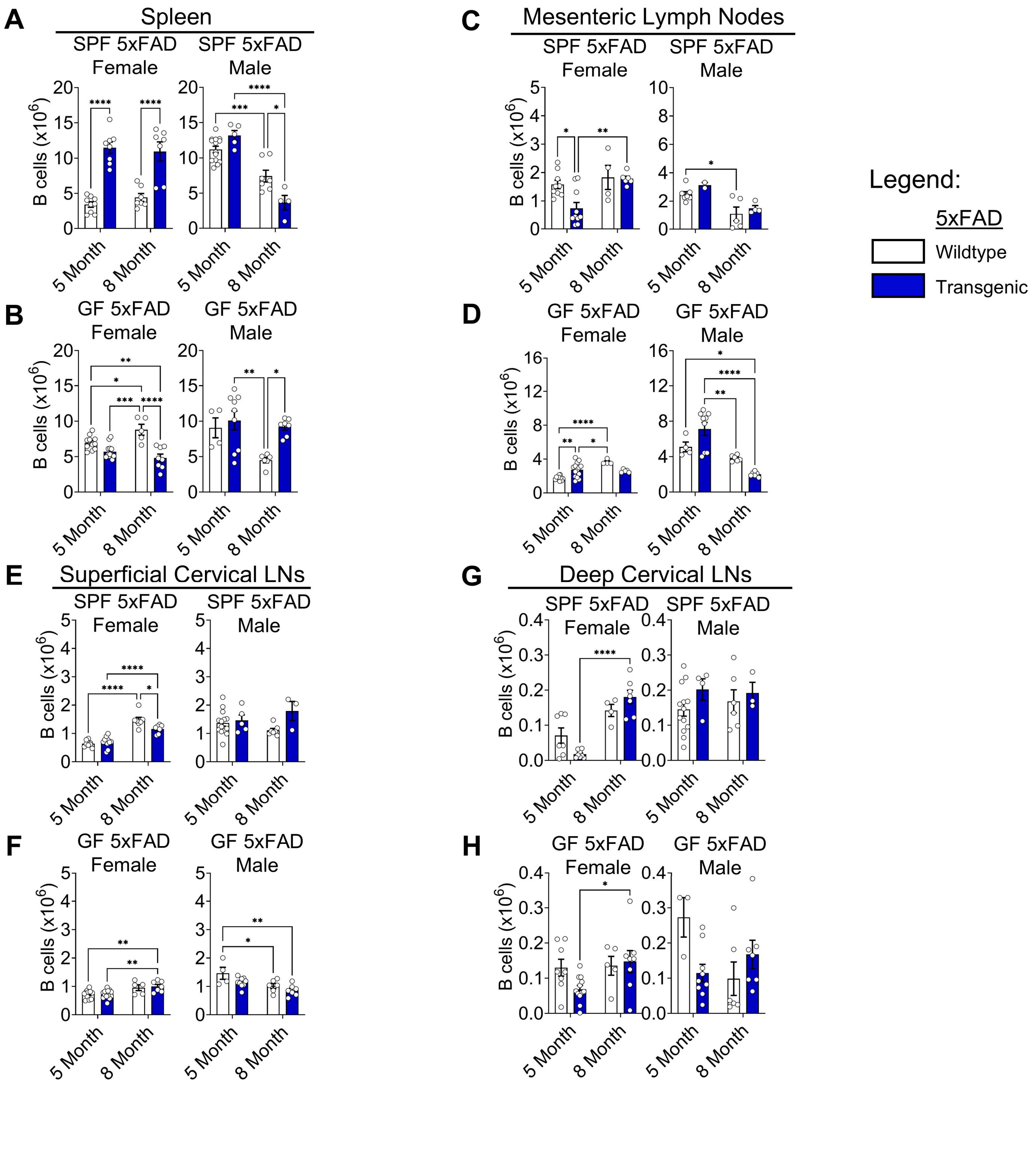

Supplementary Figure 14. Longitudinal B cell responses in 5xFAD mice.
(A) Quantification of B cell counts in the spleens of female (Left) and male (Right) 5xFAD mice compared to wildtype controls in specific pathogen-free (SPF) and (B) germ-free (GF) conditions at 5 and 8 months of age. (C) Mesenteric lymph nodes in SPF and (D) GF conditions. (E) Superficial cervical lymph nodes in SPF and (F) GF conditions. (G) Deep cervical lymph nodes in SPF and (H) GF conditions. Data are pooled from 2 independent experiments (n = 2 to 15 per group); two-way ANOVA. For clarity, only significant comparisons of the same mouse group between ages or between transgenic and control mice at the same age are shown. Error bars indicate SEM. *P < 0.05, **P < 0.01, ***P < 0.001, ****P < 0.0001.

Supplementary Figure 15.

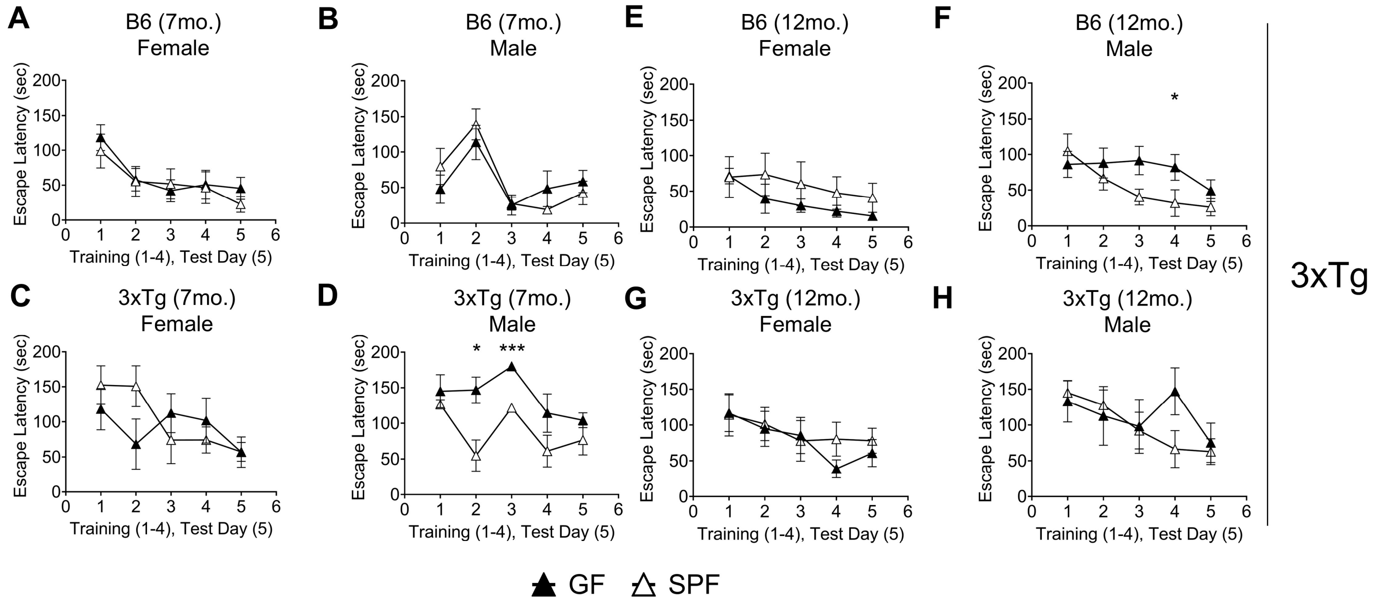

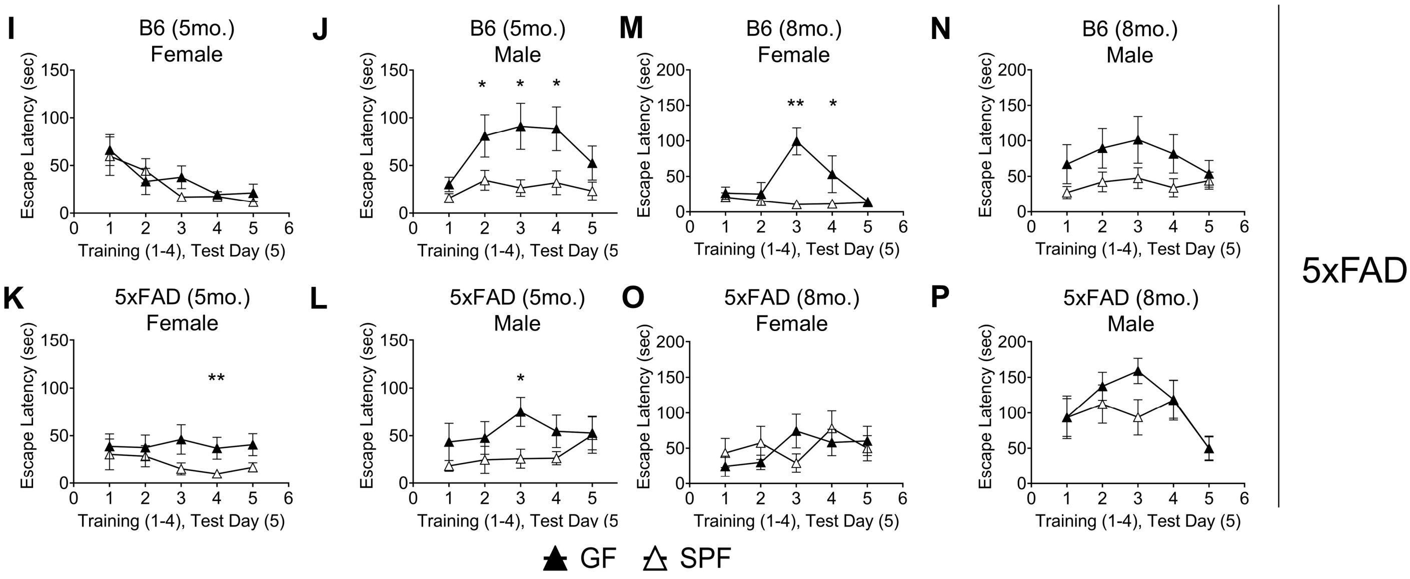

Supplementary Figure 15. Cognitive deficits in Barnes Maze testing of female and male 7-, 12- and 15-month-old 3xTg and 5- and 8-month-old 5xFAD mice [GF vs. SPF comparison].
(A) Training (1-4) and test (5) day mean escape latency in Barnes Maze for 7-month-old female wildtype (B6) mice in specific pathogen-free (SPF) compared to germ-free (GF) conditions. (B) Male B6 mice. (C) Female 3xTg mice. (D) Male 3xTg mice. Data are pooled from 2 independent experiments (n = 6 to 11 per group); Mann-Whitney U test comparing SPF to GF condition. (E) 12-month-old female B6 mice in SPF compared to GF conditions. (F) Male B6 mice. (G) Female 3xTg mice. (H) Male 3xTg mice. Data are pooled from 2 independent experiments (n = 5 to 11 per group). (I) 5-month-old female B6 mice in SPF compared to GF conditions. (J) Male B6 mice. (K) Female 5xFAD mice. (L) Male 5xFAD mice. Data are pooled from 2 independent experiments (n = 8 to 14 per group); Mann-Whitney U test comparing SPF to GF conditions. (M) 8-month-old female B6 mice in SPF compared to GF conditions. (N) Male B6 mice. (O) Female 5xFAD mice. (P) Male 5xFAD mice. Data are pooled from 2 independent experiments (n = 5 to 13 per group); Mann-Whitney U test comparing SPF to GF condition. Error bars indicate SEM. *P < 0.05, **P < 0.01, ***P < 0.001, ****P < 0.0001.

Supplementary Figure 16.

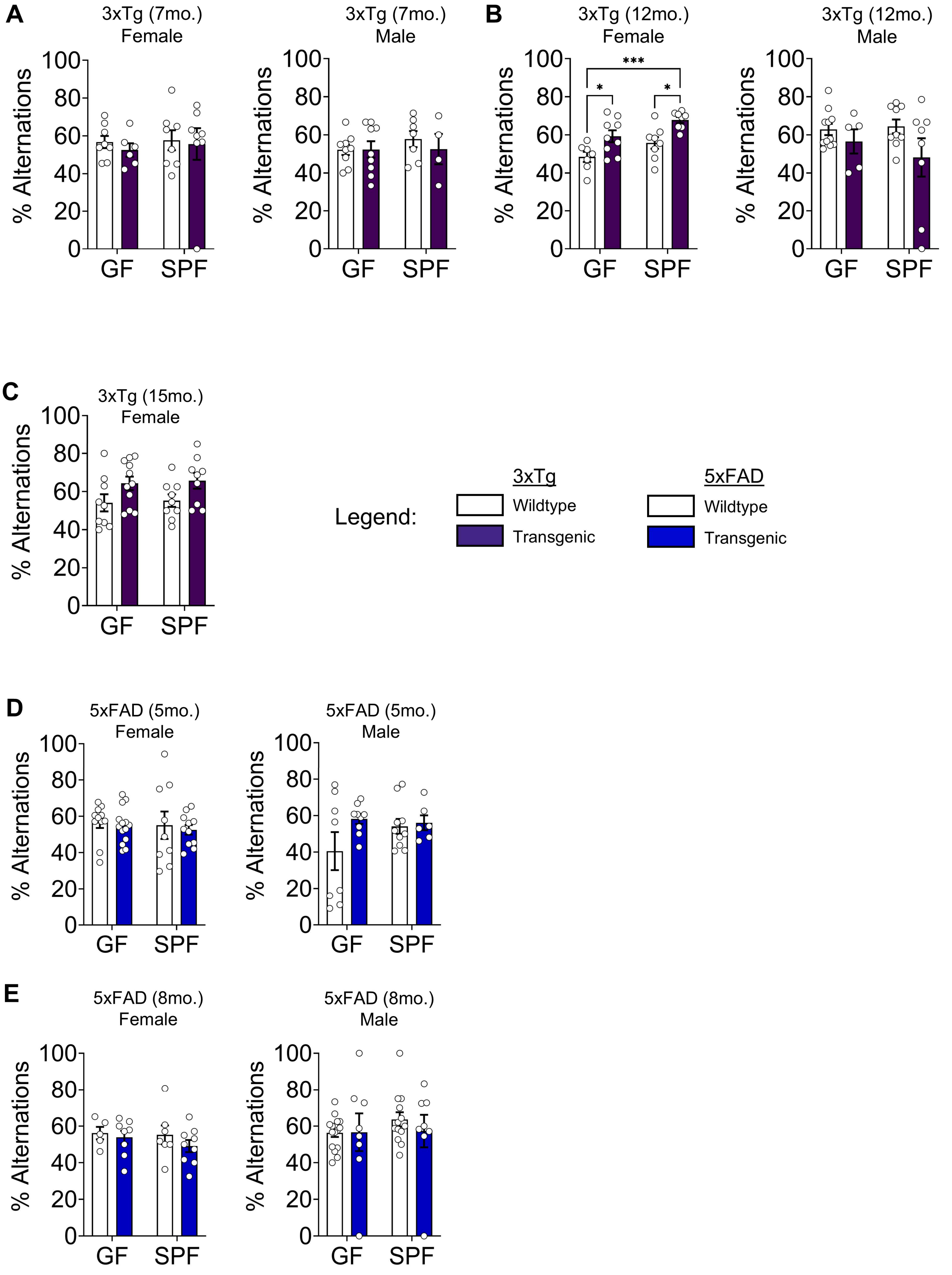

Supplementary Figure 16. Cognitive deficits in Y-maze testing of female and male 7-, 12- and 15-month-old 3xTg and 5- and 8-month-old 5xFAD mice.

(A) Alternations (%) in Y-maze testing of 7-month-old female (Left) and male (Right) 3xTg mice compared to wildtype controls in germ-free (GF) or specific pathogen-free (SPF) conditions. (B) 12-month-old female (Left) and male (Right) 3xTg mice. (C) 15-month-old female 3xTg mice. Data are pooled from 2 independent experiments (n = 2 to 14 per group); two-way ANOVA. (D) 5-month-old female (Left) and male (Right) 5xFAD mice. (E) 8-month-old female (Left) and male (Right) 5xFAD mice. Data are pooled from 2 independent experiments (n = 5 to 16 per group); two-way ANOVA. Error bars indicate SEM. *P < 0.05, **P < 0.01, ***P < 0.001, ****P < 0.0001.

Supplementary Figure 17.

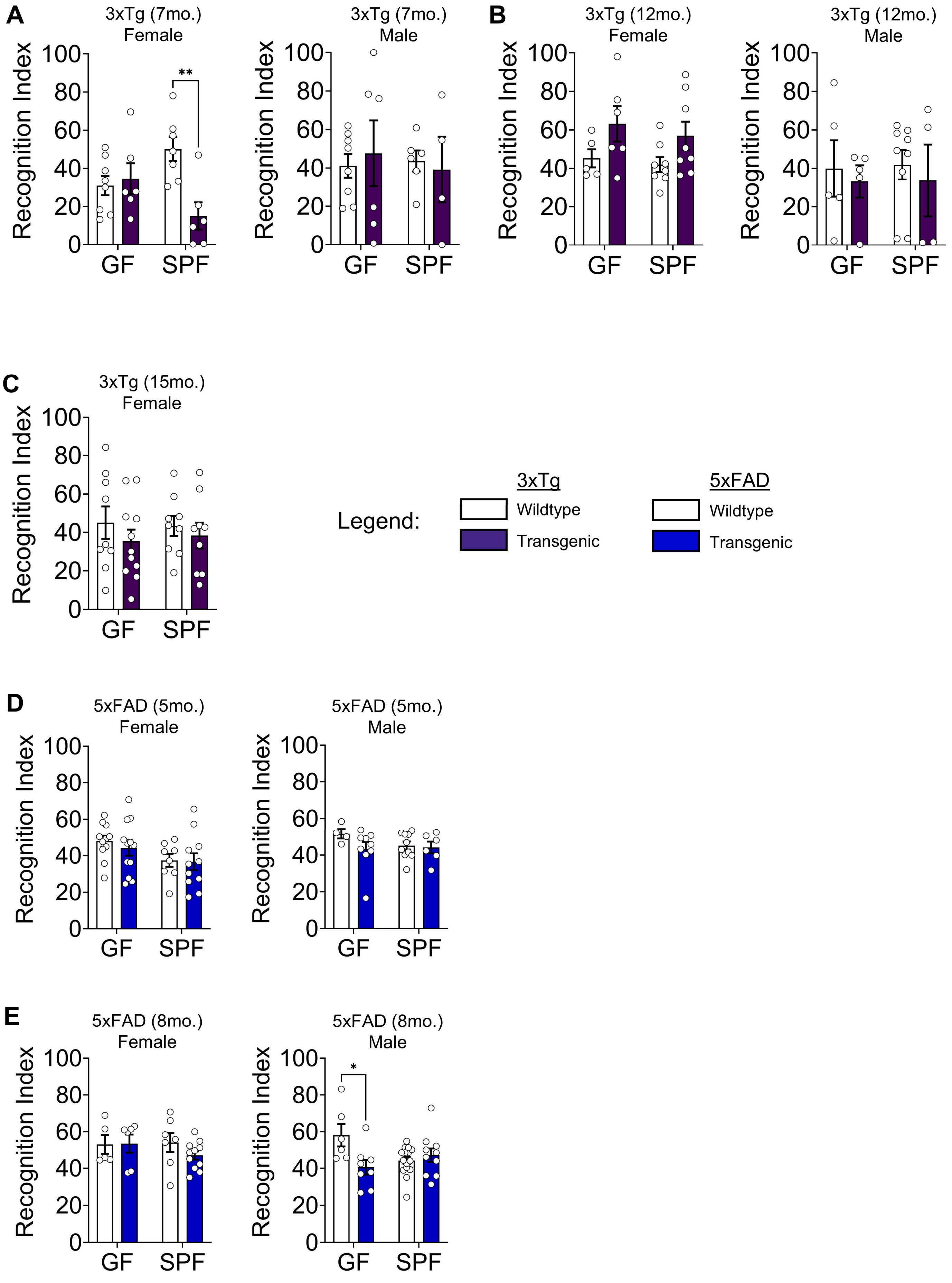

Supplementary Figure 17. Cognitive deficits in Novel Object Recognition testing of female and male 7-, 12- and 15-month-old 3xTg and 5- and 8-month-old 5xFAD mice.
(A) Recognition index in novel object recognition testing for 7-month-old female (Left) and male (Right) 3xTg mice compared to wildtype controls in germ-free (GF) or specific pathogen-free (SPF) conditions. (B) 12-month-old female (Left) and male (Right) 3xTg mice. (C) 15-month-old female 3xTg mice. Data are pooled from 2 independent experiments ($n = 4$ to 14 per group); two-way ANOVA. (D) 5-month-old female (Left) and male (Right) 5xFAD mice. (E) 8-month-old female (Left) and male (Right) 5xFAD mice. Data are pooled from 2 independent experiments ($n = 5$ to 16 per group); two-way ANOVA. Error bars indicate SEM. $^{*}P < 0.05$, $^{**}P < 0.01$, $^{***}P < 0.001$, $^{****}P < 0.0001$.
